# Transcriptome Analysis of Rice Seedling Roots in Response to Potassium Deficiency

**DOI:** 10.1038/s41598-017-05887-9

**Published:** 2017-07-17

**Authors:** Xiaoqin Zhang, Hua Jiang, Hua Wang, Jun Cui, Jiahui Wang, Jiang Hu, Longbiao Guo, Qian Qian, Dawei Xue

**Affiliations:** 1 College of Life and Environmental Sciences, Hangzhou Normal University, Hangzhou, China; 2 State Key Laboratory Breeding Base for Zhejiang Sustainable Pest and Disease Control, Zhejiang Academy of Agricultural Science, Hangzhou, China; 3 State Key Laboratory of Rice Biology, China National Rice Research Institute, Hangzhou, China; 4 Institute of Crop Germplasm and Biotechnology, Jiangsu Academy of Agricultural Sciences, Nanjing, 210014 China

## Abstract

Rice is one of the most important food crops in the world, and its growth, development, yield, and grain quality are susceptible to a deficiency of the macronutrient potassium (K^+^). The molecular mechanism for K^+^ deficiency tolerance remains poorly understood. In this study, K^+^ deficient conditions were employed to investigate the resulting changes in the transcriptome of rice seedling roots. Using ribonucleic acid sequencing (RNA-Seq) and analysis, a total of 805 differentially expressed genes were obtained, of which 536 genes were upregulated and 269 were downregulated. Gene functional classification showed that the expression of genes involved in nutrient transport, protein kinases, transcription processes, and plant hormones were particularly altered in the roots. Although these changes were significant, the expression of most genes remained constant even in K^+^-deficient conditions. Interestingly, when our RNA-Seq results were compared to public microarray data, we found that most of the genes that were differentially expressed in low K^+^ conditions also exhibited changes in expression in other environmental stress conditions.

## Introduction

Normal plant growth and development, maturation of essential tissues, and organ functions require the mineral nutrient element potassium^1^. The physiological functions of potassium include promoting chlorophyll synthesis, activating enzymes, promoting carbohydrate metabolism, and enhancing membrane permeability to ions, as well as conferring resistance to drought, frost, and pests, and enhancing antilodging activity^2, 3^.

In agricultural crops, the available potassium increases crop yield and quality. Although potassium content is abundant in the lithosphere and soil, almost 10 times more so than total nitrogen and total phosphorus, an extremely high proportion (90–98%) of potassium exists in the soil in forms that cannot be directly absorbed or used by plants^4, 5^. In particular, with the breeding and propagation of high yield and super-high yield crop varieties, the absorption of potassium ions (K^+^) from the soil increases and K^+^ deficiency becomes a serious problem for plant development over time. During K^+^ deficiency in plants, growth rate slows and can even stop, leaves become darker in color, lodging is more likely, pathogen susceptibility increases, fruit yield and quality decreases, and old leaves develop a burnt appearance^6^. Due to the high mobility of K^+^, symptoms of deficiency always appear in old leaves first; if K^+^ deficiency continues, young leaves will also exhibit symptoms of K^+^ deficiency^7^. Over the course of evolution, plants have developed strategies for responding to low K^+^ stress, and K^+^ absorption efficiency can vary greatly between different plant varieties^4, 5^.

Rice is an important grain crop and is the staple food for about half of the world’s population. Rice has a very large demand for potassium; every 1,000 kg of rice produced in a paddy requires 15–25 kg of pure potassium, which is 1.2 times the amount of nitrogen and seven times the amount of phosphorus needed for production^8^. There are widespread differences in the efficiency of potassium utilization among different rice genotypes. Some studies have reported morphological, physiological, and biochemical differences, as well as differences in K^+^ use efficiency under low K^+^ stress. These studies found there is a significant difference in K^+^ deficiency tolerance between rice genotypes^9–11^, further indicating that the potassium nutritional activities of rice are genetically regulated through gene expression that increases its efficient use or tolerance to low potassium. These results provide a theoretical foundation for the selection and cultivation of rice cultivars with low potassium tolerance. With the development of molecular techniques, more research on the molecular genetic mechanisms of potassium nutritional activities in plants is currently being conducted. Many genes for potassium transporters, potassium ion channels, and related regulatory proteins have been cloned to study K^+^ flux and protein localization. In addition, forward and reverse genetic analyses have become important molecular tools for observing the regulation of potassium nutritional activities (absorption and transport) in plants^1, 12^. Although genes related to efficient nutritional activity have already been cloned for functional and regulatory analyses and have applications in the improvement of potassium nutrition in crops, investigations focused on the functional genomics and molecular genetic regulatory networks for high nutritional efficiency in plants are still ongoing.

Transcriptomics has already been broadly adopted in functional genomics research. With the rapid development of sequencing technology, the application of RNA-Seq has become an important new method for gene expression and transcriptome analysis^13^. When compared to gene microarray technology, RNA-Seq has an even greater sensitivity and resolution for gene expression research and transcriptional profiling^14, 15^. In addition, it has advantages for identifying new transcripts, single nucleotide polymorphisms, gene structures, and splice variants^16, 17^. Currently, this technology is already broadly utilized in the study of differential gene expression during plant responses to various biological and nonbiological stressors^18–23^.

We previously screened a group of rice germplasm accessions that are tolerant of low potassium during the seedling phase^24^. Additional experiments have confirmed that multiple genes regulate low potassium tolerance in rice and a partial quantitative trait locus for low potassium stress was identified^25^. However, there are few data on the transcriptomics of the response to low potassium stress in rice. The present study used Nipponbare rice as source material and Illumina RNA-Seq technology combined with real-time fluorescent quantitative PCR (QPCR) to analyze and compare the rice root transcriptome under low and normal potassium conditions, and to identify signal transduction pathways and regulatory networks related to low potassium tolerance.

## Results

### Transcriptome sequencing in rice roots identified differentially expressed genes sensitive to K^+^ deficiency in rice

An Illumina HiSeq™ 2500 was used to conduct high-throughput transcriptome analysis of control and mixed low potassium stress-treated rice root samples at three time points; 11,432,307 and 15,120,257 clean reads were obtained, respectively, and accounted for over 98% of the total sequences (Table S1). FASTQC was used to assess sequence quality in both control and treatment samples (Figure S2).

**Figure 1:**
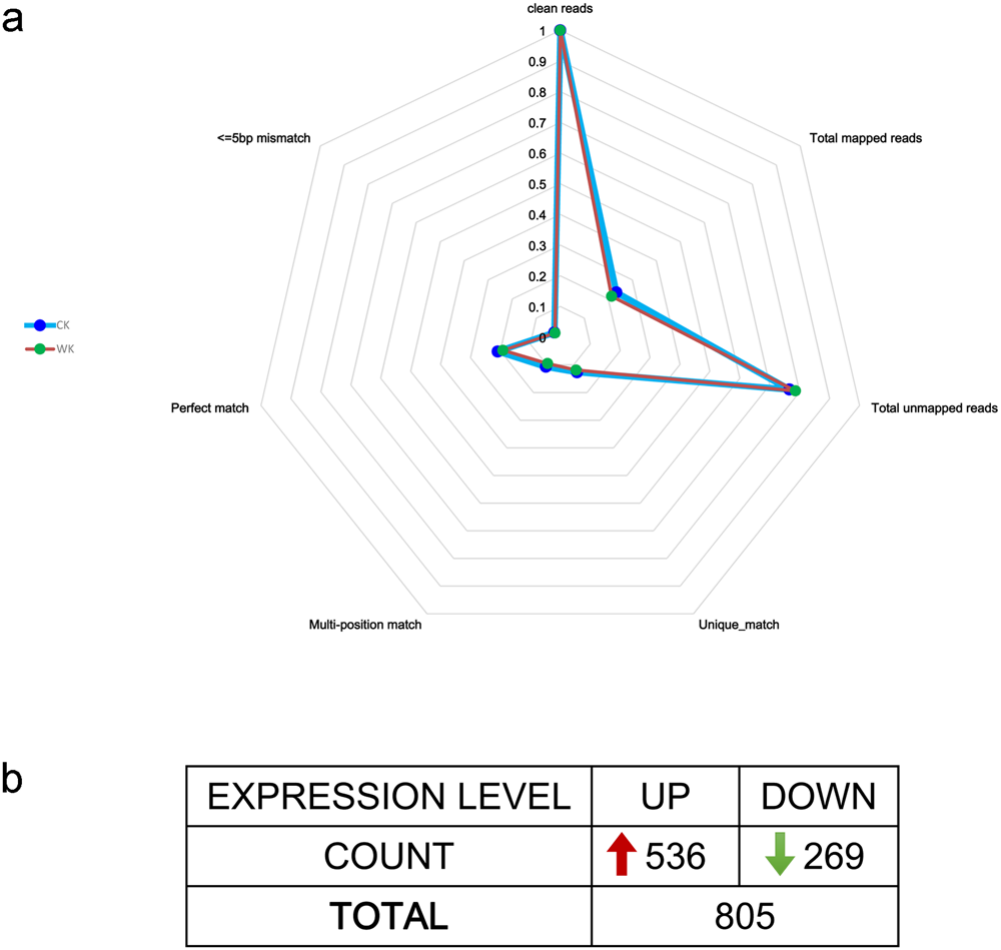
The results of transcriptome sequencing analysis under K^+^ stress conditions. (**a**) The comparing results from the mapping procedure of transcriptome sequencing between ck and K^+^ stressed sample; (**b**) The summary of differentially expressed rice genes detected in the transcriptome sequencing.

Transcriptome analysis was conducted using the complete genomic sequence of Nipponbare rice as a reference. The length of the longest gene obtained was over 4,000 bp; the lengths of most genes were distributed between 1,000 and 2,500 bp (Table S3). Most gene sequences were well matched with the reference genome (Table S4). The majority of the matched genes had a coverage of 60% or more (Figure S5 and Fig. 1a).

The reads per kilobase of transcript per million mapped reads calculation method was employed to compare transcriptome differences between the control and the treated group. This study yielded a total of 805 differentially expressed genes, of which 536 genes were upregulated and 269 genes were downregulated (Table S6 and Fig. 1b). A threshold of two-fold or above was used as a standard for evaluating differential gene expression. Among these, the expression level of the most upregulated gene was 52-fold that of the control, and the expression level of the most downregulated gene was 79-fold below that of the control (Table S6). There were 98 downregulated genes with a three-fold or more decrease in expression level, and there were 219 upregulated genes with a three-fold or more expression level increase (Table S6). Among genes with a five-fold or higher difference in expression, 58 genes were upregulated and 47 genes were downregulated. Among genes with a 10-fold or higher difference in expression, 19 genes were downregulated and 14 genes were upregulated (Table S6).

### Functional classification of differentially expressed genes identified in low K^+^ conditions

**Figure 2:**
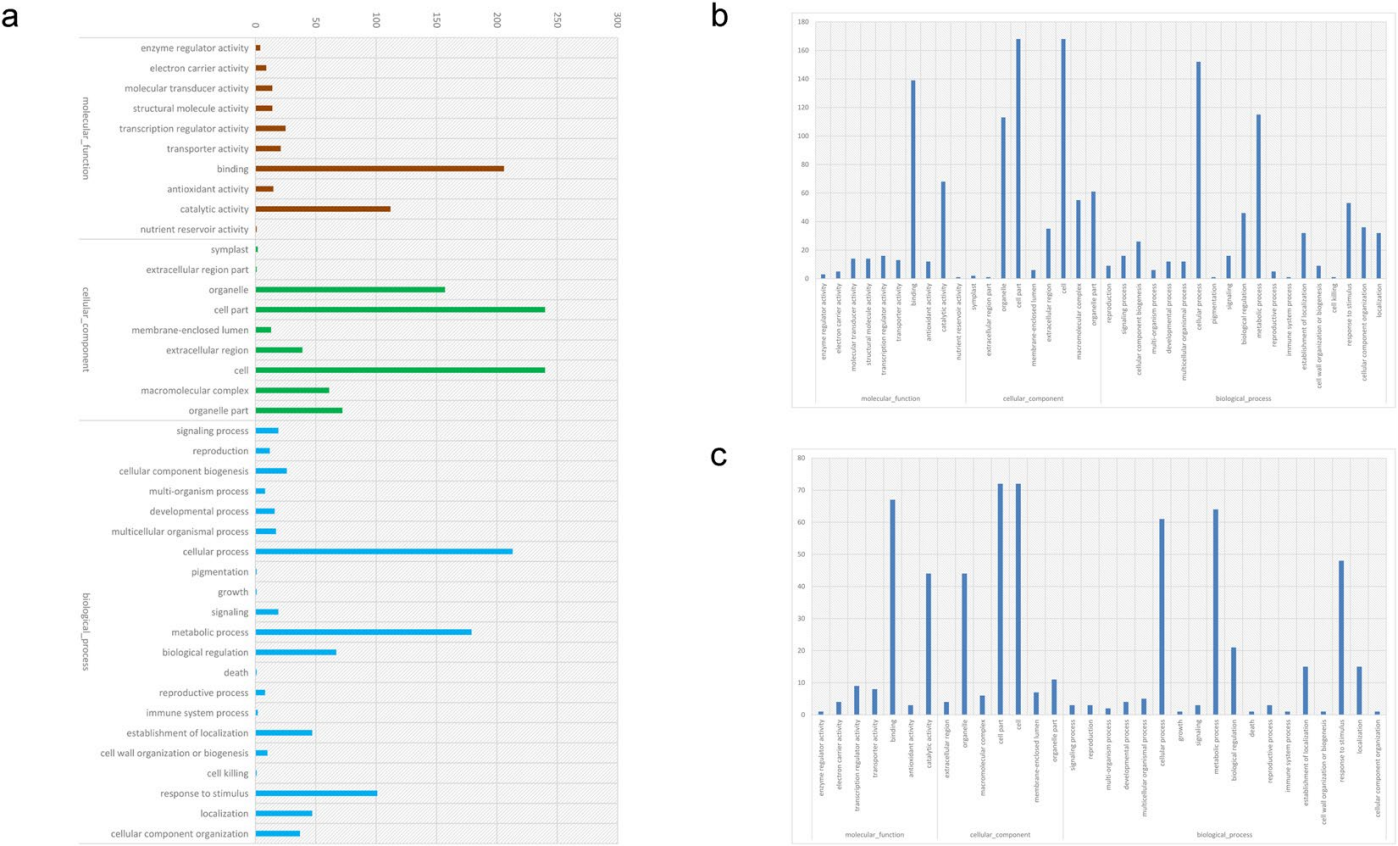
GO classification of differentially expressed genes in the transcriptome sequencing experiment. (**a**) The GO classification of all 805 differentially expressed genes detected in this study; (**b**) GO classification of the upregulated differentially expressed genes; (**c**) GO classification of the downregulated differentially expressed genes.

To better understand the function of the genes affected by low K^+^, we used GoPipe to conduct GO classification on the differentially expressed genes. A total of 12,414 predicted proteins were matched with 81,604 GO terms. Among the molecular functions, genes that encoded proteins with binding and catalytic activity accounted for a large portion. Additionally, genes encoding proteins localized in organelles also accounted for a large number of those that were differentially expressed under K^+^ deficiency. Many of these genes participate in cell growth and metabolism, and their expression was stimulated by adverse environmental conditions (Fig. 2a). GO classification analysis of the differentially expressed genes revealed that these genes involved in a very large portion of the whole life process (Fig. 2b,c).

**Figure 3:**
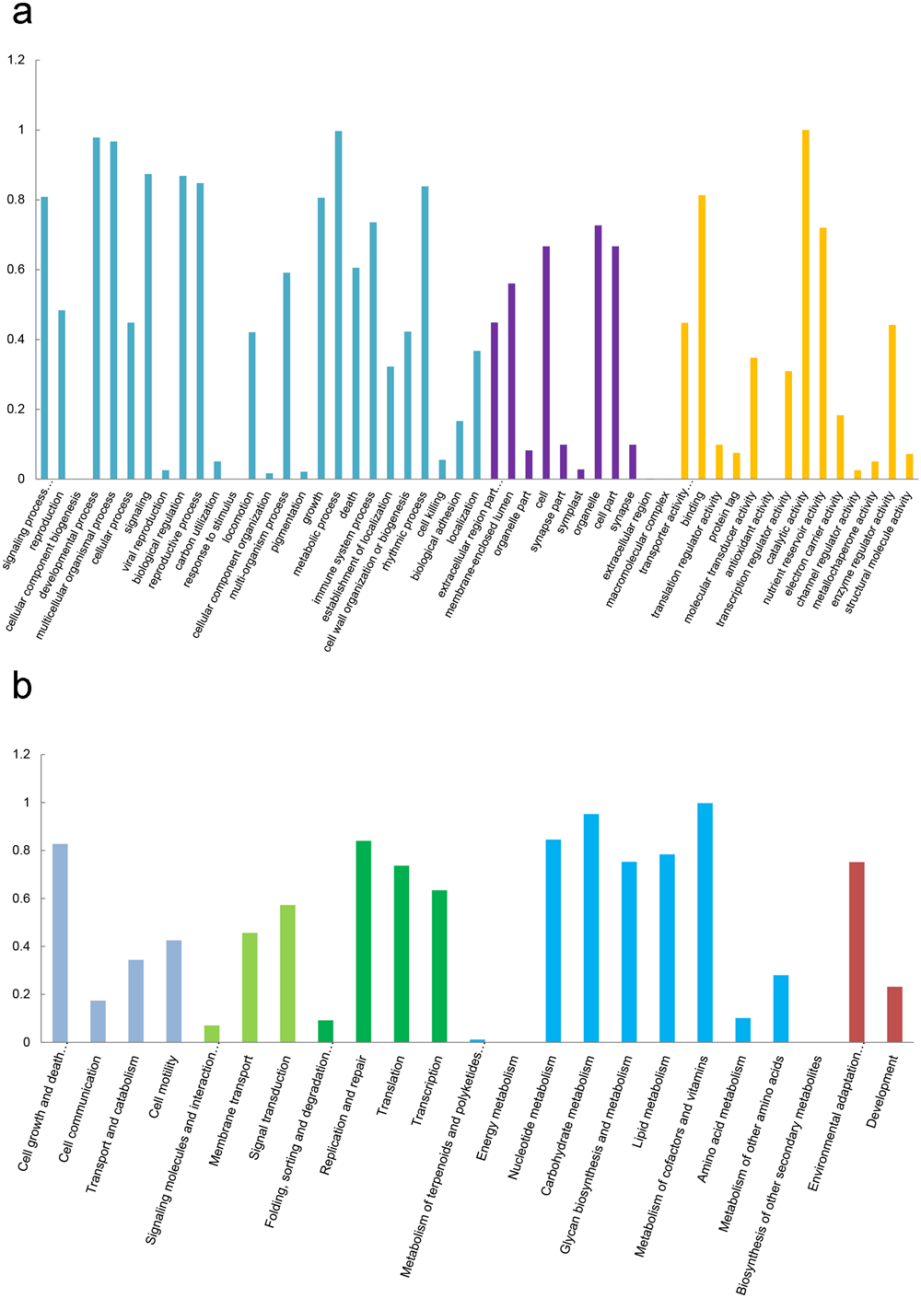
Gene enrichment analysis of differentially expressed genes in the transcriptome sequencing experiment. (**a**) GO enrichment analysis of all differentially expressed genes detected in this study; (**b**) KEGG enrichment analysis of all differentially expressed genes detected in this study.

To further understand the functions of the differentially expressed genes and the related biological processes they participate in, GO and KEGG enrichment analysis were conducted (Fig. 3). GO enrichment showed that genes with functions related to catalytic activity, binding activity, and nutrient storage were up or downregulated under conditions of K^+^ stress. The biological processes that these genes primarily participate in involve metabolism, growth and development, multicellular processes, and signal transduction processes (Fig. 3a). KEGG analysis also showed that the differentially expressed genes were primarily involved in metabolic pathways and signal transduction (Fig. 3b). Enrichment analysis also showed that the expression of genes involved in replication and transcriptional pathways are strongly affected, implying that K^+^ stress also affects the normal biological functions of root cells.

### Real-time fluorescent quantitative analysis confirms differential expression of genes in response to low K^+^

**Figure 4:**
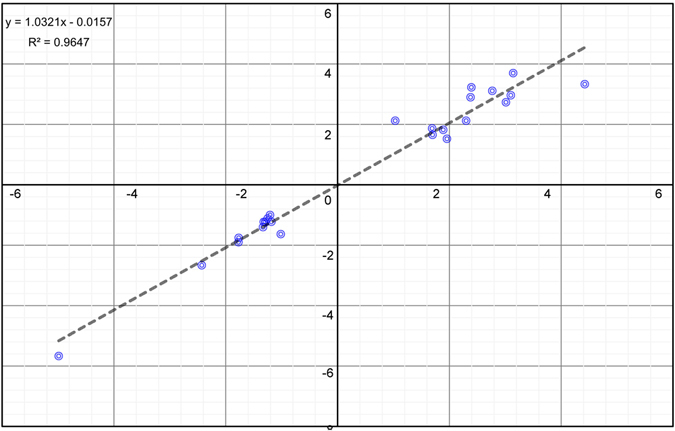
Correlation analysis of differentially expressed genes between QPCR analysis method and transcriptome sequencing experiment.

Transcriptome analysis results show that the expression of many genes changed. To further validate the accuracy of these expression differences, 24 genes among the differentially expressed genes were selected for fluorescent quantitative validation according to their expression pattern (Table S7). Of these, 14 genes were upregulated and 11 genes were downregulated (Fig. 4). Analysis results showed that there was good correlation between the RNA-Seq and QPCR experiments (R^2^ = 0.9647), and that the changes in expression of most differentially expressed genes agreed with the results of the quantitative analysis.

### Gene families are differentially expressed under low K^+^

**Figure 5:**
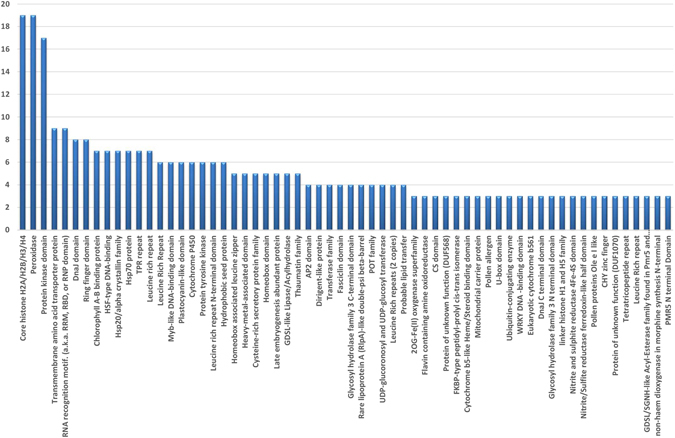
The summary of gene families represented in differentially expressed genes.

Rice has many gene families, and members of the same family all have very important effects upon the same biological processes. To further understand the gene families whose expression was affected by low K^+^ stress, we identified families represented in our list of differentially expressed genes (Table S8). Results showed that members from gene families of core histones H2A/H2B/H3/H4, peroxidases, protein kinases, and transmembrane amino acid transporter proteins closely related to ion transport accounted for many of the genes differentially expressed under low K^+^ conditions. This implies that genes in these families were the most affected by low K^+^ stress, and also shows that these gene families may be important for plant adaptability to low K^+^ stress conditions (Fig. 5).

### Identification of transcription factors that respond to low K^+^

High-throughput sequencing is a more sensitive assay for gene expression when compared to microarrays; it can capture the expression of less abundant genes, such as transcription factors. Further analysis of differentially expressed genes in rice roots showed that there were 47 transcription factors belonging to 22 transcription factor families (Table 1). Of these, heat stress transcription factors and APETALA 2/ethylene-responsive element binding factor family members were the most numerous. The heat stress transcription factors family is related to various stress responses in plants, and the ethylene response factor family primarily affects DNA binding. Other transcription factors are involved in biological processes, including signal transduction and defense against adverse environmental conditions.

**Table 1:** The transcription factors were identified in rice root respond to low K^+^.

#Gene	Function	read_A	RPKM_A	read_B	RPKM_B	log2 (Fold_change) normalized	q-value (Benjamini et al. 1995)	p_value	Result
**B3**									
LOC_Os11g09160.1	gi|108864092|gb|ABA91888.2| B3 DNA binding domain containing protein, expressed	42	8.696509133	121	19.85700082	−1.191139426	2.09138E-05	7.57682E-07	down
**bHLH**									
LOC_Os10g40740.1	hypothetical protein OsI_34609	51	10.38901156	22	3.551888718	1.548400112	0.00022182	1.04738E-05	up
LOC_Os01g06640.1	gi|9757687|dbj|BAB08206.1| unnamed protein product	30	7.940804334	87	18.25135486	−1.200646512	0.000463056	2.43871E-05	down
**bZIP**									
LOC_Os07g08420.1	gi|13365770|dbj|BAB39173.1| RISBZ1	24	5.248145725	116	20.1041284	−1.937612106	2.82623E-10	4.46275E-12	down
**C2H2**									
LOC_Os08g44050.1	gi|45736048|dbj|BAD13075.1| putative zinc finger protein	68	10.7819266	31	3.89566648	1.468672919	2.67626E-05	9.87701E-07	up
LOC_Os03g13600.1	gi|108707079|gb|ABF94874.1| Zinc finger, C2H2 type family protein, expressed	59	18.17039184	28	6.834439903	1.410694516	0.000208503	9.73587E-06	up
**C3H**									
LOC_Os05g03760.1	gi|51854363|gb|AAU10743.1| putative finger transcription factor	469	89.76929216	271	41.11088871	1.12670146	2.70228E-23	1.28094E-25	up
**CO-like**									
LOC_Os04g42020.1	gi|38345390|emb|CAE03116.2| OSJNBa0067K08.19	70	19.57858285	21	4.655165487	2.072371983	1.41492E-08	2.77863E-10	up
**DBB**									
LOC_Os02g39360.1	gi|3618314|dbj|BAA33203.1| zinc finger protein	375	135.6308532	177	50.73797252	1.418547624	1.02694E-26	4.17252E-29	up
**Dof**									
LOC_Os04g47990.1	hypothetical protein OsI_17020	120	30.0689069	62	12.31290986	1.288100674	1.744E-07	4.02073E-09	up
**ERF**									
LOC_Os07g12510.1	gi|50510280|dbj|BAD31677.1| pathogenesis-related genes transcriptional activator Pti6-like protein	68	31.2282185	26	9.46333602	1.722429512	1.42219E-06	3.95733E-08	up
LOC_Os01g58420.1	gi|9309342|dbj|BAB03248.1| ethylene responsive element binding factor3	93	34.17539569	48	13.97991262	1.289602699	6.73193E-06	2.17159E-07	up
LOC_Os09g20350.1	gi|49387678|dbj|BAD25924.1| DRE binding factor 2	347	150.4928165	185	63.59026576	1.24281678	3.85151E-20	2.26434E-22	up
LOC_Os03g08490.1	gi|113547650|dbj|BAF11093.1| Os03g0183200	219	114.0009011	118	48.68316366	1.227550399	1.65698E-12	1.94321E-14	up
LOC_Os03g08460.1	gi|33087061|gb|AAP92744.1| ap2 domain containing protein	182	53.71734723	99	23.15852955	1.213844409	2.93012E-10	4.6358E-12	up
LOC_Os03g08500.1	gi|56567587|gb|AAV98703.1| BTH-induced ERF transcriptional factor 4	429	140.3224064	257	66.62473142	1.074615677	9.84978E-20	5.94237E-22	up
**G2-like**									
LOC_Os06g35140.1	gi|53793087|dbj|BAD54297.1| MYB transcription factor-like	44	8.842945197	17	2.70785743	1.707375166	0.000236532	1.1274E-05	up
LOC_Os02g04640.1	gi|42409277|dbj|BAD10540.1| putative transfactor	137	28.77985372	487	81.08295965	−1.494339491	9.37185E-30	3.40396E-32	down
**GRAS**									
LOC_Os07g40020.1	hypothetical protein OsJ_24947	58	10.4211164	29	4.129684441	1.335406388	0.000490288	2.60175E-05	up
**HD-ZIP**									
LOC_Os09g29460.1	gi|5006853|gb|AAD37697.1|AF145728_1 homeodomain leucine zipper protein	83	22.15000678	29	6.13375444	1.852464825	9.27157E-09	1.75797E-10	up
LOC_Os08g37580.1	gi|42409030|dbj|BAD10283.1| putative homeodomain leucine zipper protein	73	20.20030161	30	6.579439003	1.618340352	1.82949E-06	5.19486E-08	up
LOC_Os09g21180.1	RecName: Full = Homeobox-leucine zipper protein HOX25; AltName: Full = HD-ZIP protein HOX25; AltName: Full = Homeodomain transcription factor HOX25; AltName: Full = OsHox25	206	62.99455648	109	26.41769468	1.253722591	3.61825E-12	4.4326E-14	up
LOC_Os10g23090.1	gi|78708410|gb|ABB47385.1| Homeobox domain containing protein, expressed	66	19.85568713	36	8.583731069	1.209875506	0.00059967	3.26525E-05	up
LOC_Os08g32080.1	gi|5006855|gb|AAD37698.1|AF145729_1 homeodomain leucine zipper protein	77	23.62061286	44	10.69758459	1.14276131	0.000354887	1.80568E-05	up
LOC_Os03g43930.1	hypothetical protein OsJ_11861	100	20.79431962	58	9.558842919	1.121281583	3.90619E-05	1.49211E-06	up
**HSF**									
LOC_Os03g53340.1	gi|30017583|gb|AAP13005.1| putative heat shock factor	7	1.231057487	261	36.37922288	−4.885144686	8.16552E-53	1.35723E-55	down
LOC_Os03g53340.1	gi|30017583|gb|AAP13005.1| putative heat shock factor	7	1.231057487	261	36.37922288	−4.885144686	8.16552E-53	1.35723E-55	down
LOC_Os08g43334.1	gi|42408097|dbj|BAD09238.1| putative heat shock factor RHSF2	3	0.78082809	41	8.457668263	−3.437183116	6.89573E-07	1.80947E-08	down
LOC_Os01g39020.1	gi|52076304|dbj|BAD45089.1| heat shock transcription factor HSF8-like	2	0.510666002	26	5.261540459	−3.36503333	0.000192551	8.83396E-06	down
LOC_Os09g35790.1	gi|52077316|dbj|BAD46357.1| putative heat shock factor	13	2.886455243	97	17.06969755	−2.564066736	4.60322E-12	5.72427E-14	down
LOC_Os09g35790.1	gi|52077316|dbj|BAD46357.1| putative heat shock factor	13	2.886455243	97	17.06969755	−2.564066736	4.60322E-12	5.72427E-14	down
LOC_Os10g28340.1	gi|31432122|gb|AAP53792.1| HSF-type DNA-binding domain containing protein, expressed	43	12.54896721	133	30.76265296	−1.293611292	1.15434E-06	3.15874E-08	down
LOC_Os03g06630.1	predicted protein	31	5.322192108	90	12.24627418	−1.202250398	0.000341571	1.73057E-05	down
**LSD**									
LOC_Os08g03610.1	gi|29467531|dbj|BAC66720.1| putative zinc-finger protein Lsd1	112	50.60031424	353	126.3986726	−1.320763063	7.41044E-18	5.22344E-20	down
**MIKC**									
LOC_Os02g49840.1	gi|30313689|gb|AAO47712.1| transcription factor MADS57	13	3.662294369	91	20.31813799	−2.471948534	6.99002E-11	1.00693E-12	down
**MYB**									
LOC_Os04g42950.1	gi|125548977|gb|EAY94799.1| hypothetical protein OsI_16582	7	1.989252514	57	12.83805842	−2.690128704	1.4708E-07	3.36372E-09	down
LOC_Os01g65370.1	gi|19386839|dbj|BAB86217.1| putative myb-related protein	73	27.55297674	30	8.974278368	1.618340352	1.82949E-06	5.19486E-08	up
**MYB_related**									
LOC_Os01g09280.1	gi|55771329|dbj|BAD72254.1| putative MybSt1	31	8.440431757	108	23.30554916	−1.465284803	1.51254E-06	4.21806E-08	down
LOC_Os08g39980.1	gi|28411872|dbj|BAC57402.1| DNA-binding protein family-like	147	22.76185204	74	9.081437477	1.325625368	1.61096E-09	2.77683E-11	up
**NAC**									
LOC_Os12g03040.1	gi|113644331|dbj|BAF27472.1| Os11g0126900	60	14.32554168	153	28.95235195	−1.015090859	2.77213E-05	1.02649E-06	down
NF-YA									
LOC_Os08g09690.1	gi|38637163|dbj|BAD03416.1| putative CCAAT box binding factor/transcription factor Hap2a	19	7.991344513	94	31.33478526	−1.97125495	1.49218E-08	2.93493E-10	down
**SRS**									
LOC_Os06g49830.1	gi|53792887|dbj|BAD54064.1| putative LRP1	25	8.439253091	5	1.337724322	2.657334483	0.000257162	1.24433E-05	up
**TALE**									
LOC_Os10g39030.1	gi|22002143|gb|AAM88627.1| putative homeodomain protein	42	6.831675719	13	1.675922462	2.027284093	3.77648E-05	1.44024E-06	up
LOC_Os07g03770.1	gi|3327240|dbj|BAA31688.1| OSH15	64	17.03288979	35	7.38259464	1.206123371	0.000792451	4.49059E-05	up
**WRKY**									
LOC_Os01g54600.1	hypothetical protein OsJ_03461	54	19.71371593	24	6.944141988	1.50533139	0.000193397	8.92037E-06	up
LOC_Os08g13840.1	TPA_inf: WRKY transcription factor 25	172	61.58097407	99	28.09221077	1.132314523	1.04697E-08	2.00126E-10	up
LOC_Os04g51560.1	gi|38346908|emb|CAE03880.2| OSJNBb0015N08.8	687	234.7562047	423	114.5600848	1.035058824	8.58044E-30	3.0901E-32	up

### Construction of protein interaction networks responsive to K^+^ stress

To further understand the relationships between the differentially expressed genes in more detail and to search for possible critical pathways, protein-protein interaction analysis was conducted on the genes identified in our sequencing experiment (Figure S9). The gene with the most abundant connection points was the Putative activator of 90-kDa heat shock protein adenosinetriphosphatase homolog 1, which was downregulated in this study (Figure S9). Because life processes were highly related with energy, the identification of many differentially expressed genes involved in energy-related process seems quite reasonable. Results showed that upregulated genes were involved in transcription, implying that K^+^ stress affects the transcription process and the role of K^+^ involved in transcription needs further investigation.

### Microarray data analysis confirms genes related to K^+^ deficiency are also involved in other environmental stress responses

To further understand the behavior of the differentially expressed genes identified in this experiment in other adverse environments and stresses, microarray data from public databases were used to analyze their expression profiles under conditions of pathogen assault^32^, drought, salt, and low temperature stress^33^. Results showed that most genes that were significantly differentially expressed in low K^+^ were also responsive to other adverse environmental conditions, suggesting that genes related to K^+^ stress are not limited to ion transport, but also play important roles in plant responses to several adverse environmental conditions (Figures S10, S11 and S12).

## Discussion

While K^+^ is important for plant growth and development, the amount of K^+^ that plants can absorb directly from the soil is very limited. This is not only because the amount of free and readily useable K^+^ in the soil is low, but it is also due to an increased grain yield and cropping index, which depletes K^+^ from the soil and leads to insufficiently supplemented soils over time. According to statistical analyses, one-quarter of the soil in the global tropical and subtropical zones is in a state of K^+^ deficiency^34^. This forces plants in these regions to grow in low K^+^ environments for long periods. To adapt to this environment, plants must take various actions to promote K^+^ absorption. To effectively absorb K^+^ in environments with low potassium availability, plants initiate two important mechanisms to improve the efficiency of K^+^ uptake^35^. In addition, to transport potassium more effectively, K^+^ transport also needs to be more efficient. Nutrient content and availability in the soil complicate K^+^ uptake, as does how plants access and utilize the nutrient; this suggests that regulation at the molecular and cellular levels may also be highly complex. All of these activities require the participation of a series of genes and may also require certain organs to change shape (Figure S13)^1^.

Low K^+^ stress affects the growth and development of rice. This study shows that low K^+^ stress during the seedling phase results in increased Malonic Dialdehyde (MDA) content, MDA is an important indicator for membrane lipid peroxidation, which indicates membrane system damage, suggesting an oxidative stress aggravation in roots. Correspondingly, the activity of some antioxidases also increased (Figure S14). As the primary plant organ that directly contacts the soil, the roots sense and absorb K^+^. Analyses of mechanisms related to K^+^ uptake and roots are integral to the understanding of the molecular regulation induced by low K^+^ conditions and provide a model for addressing plant responses to K^+^ soil deficiency. Plant roots are responsible for detecting external K^+^ concentrations and their fluctuations during plant growth. Although no potassium receptor has been identified, research has shown that shaker transporter proteins are involved in the detection and absorption of K^+^ in rice^48^. Significant changes in the expression of the shaker transporter proteins were not detected in the present study, which may be due to differences between their expression in the roots and in other parts of the plant. Protein kinases, particularly pyruvate kinases^36^, play an important role in intracellular detection of K^+^. The present study found two similar pyruvate kinases, one upregulated and the other downregulated. In addition, protein kinases also have K^+^ channel regulating activity^1^. These genes should be closely related to the regulation of K^+^ channels. We identified genes clearly annotated as encoding protein kinases; 21 genes were upregulated and six were downregulated. In addition, the upregulated protein kinases included mitogen-activated protein kinase pathway genes, implying that these kinases may be responsible for K^+^ signal transduction. Previous research had always considered calcium and reactive oxygen species (ROS) as important secondary messengers of K^+^ signal transduction^1^; however, kinase signal transduction functions have not been studied in detail.

Plant hormones are required in each of the growth phases of plants. In low K^+^ stress conditions, hormones similarly play an important role. In this study, genes related to jasmonic acid, auxin, and ethylene were the most abundant genes involved hormone-related processes, which is consistent with previous reports^37^. Two genes in the *ASR* gene family in rice, *OsASR4* and *OsASR6*, were detected in this study and were both downregulated. Previous research has shown that this gene family has different effects on different hormones and has a certain enhanced effect under adverse environmental conditions^38^. Additionally, the genes in this family are related to aluminum stress responses^39^, implying that there may be crosstalk between heavy metal stress and potassium stress response pathways in plants. However, previous research has concentrated on interaction between K^+^ and other ions such as Na^+^ and NH_4_^+^ and has not focused on the relationship between heavy metals and potassium ions. Pyrrolysine gene family members, which are possible receptors for abscisic acid^40^, were regulated somewhat differently; *OsPYL6* was downregulated and *OsPYL9* was upregulated. These two genes can significantly increase drought and cold tolerance in rice^40^, suggesting that there may be a close relationship between K^+^ stress and other nonbiological stressors. The *OsHAK1* gene is the rice homolog of the *Arabidopsis HAK* gene and belongs to the KT/KUP/HAK gene family, which is closely related to potassium transport. New research by Chen *et al*. shows that just as the *Arabidopsis HAK* gene plays important roles in potassium transport, the *OsHAK1* gene does so regardless of whether potassium conditions are low in rice^41^. In their study, the *OsHAK1* gene was significantly upregulated under K^+^ stress conditions^41^, but in this study, *OsHAK1* was among the downregulated genes, possibly because of differences in sample mixing and the time of sample collection (Figure S14). Gene expression studies under long-term K^+^ stress conditions would benefit from the study of how the expression of genes that are essential for K^+^ transport changes over time and from gaining a deeper understanding of how gene expression changes in rice under K^+^ stress.

ROS have been an important component in research on K^+^ stress^1^. The present study identified many peroxidases that were upregulated, indicating that ROS are important in plant responses to low potassium. However, there are currently very few reports on the interactions between ROS and K^+^. The level of participation of ROS in signal transduction and K^+^ transport is not clear and requires further research. In addition, some of these peroxidases are also related to other biological stressors, and we observed that the gene expression for some proteins related to disease was upregulated. Thus, the relationship between K^+^ stress and biological stressors warrants further investigation. Genes related to ethylene in this study were upregulated, showing that ethylene has a positive function in the response to K^+^ stress. In addition, a large number of genes related to auxin was also upregulated, indicating the possibility of hormonal participation in plant morphology during K^+^ stress. Genes related to rice root morphology had differential expression levels, implying that rice root morphology is also susceptible to K^+^ stress.

The growth of rice is regulated by many environmental factors. This study showed that the effects of K^+^ stress on rice are not only limited to kinase and transporter proteins but also have a very large effect through changes in the expression of genes encoding heat shock proteins and other transcription factor families, implying that K^+^ stress is closely related to temperature. Ion transport in the plant body inevitably involves many transmembrane proteins. We found that the upregulation of genes for transmembrane proteins were significant for three members of the transforming growth factor beta receptor family. However, the role of this gene family in K^+^ stress in rice has not been documented and awaits further experimental confirmation.

With the continued advancement of functional genomics research in rice, more K^+^ transport proteins and their functions, such as OsHAK, OsAKT, and OsHKT, have been confirmed^42–44^. Recent research has found that there are many other ions that promote or inhibit K^+^ absorption. Lan *et al*. found that the high-affinity K^+^ transporter in rice can also mediate Ca^2+^ transport^45^. Ma *et al*. utilized gene microarrays to analyze gene expression in rice under long-term and short-term K^+^ stress and found that many groups of genes, especially protein kinases and metal ion transporter family, were significantly upregulated^46^. Although genes related to efficient nutritional activity have already been cloned and characterized, further investigation of the functional genomics and molecular genetic regulatory networks, and their possible application in the improvement of potassium nutritional activities in crops plants, should be a focus of future research^1, 12, 47^.

**Figure 6:**
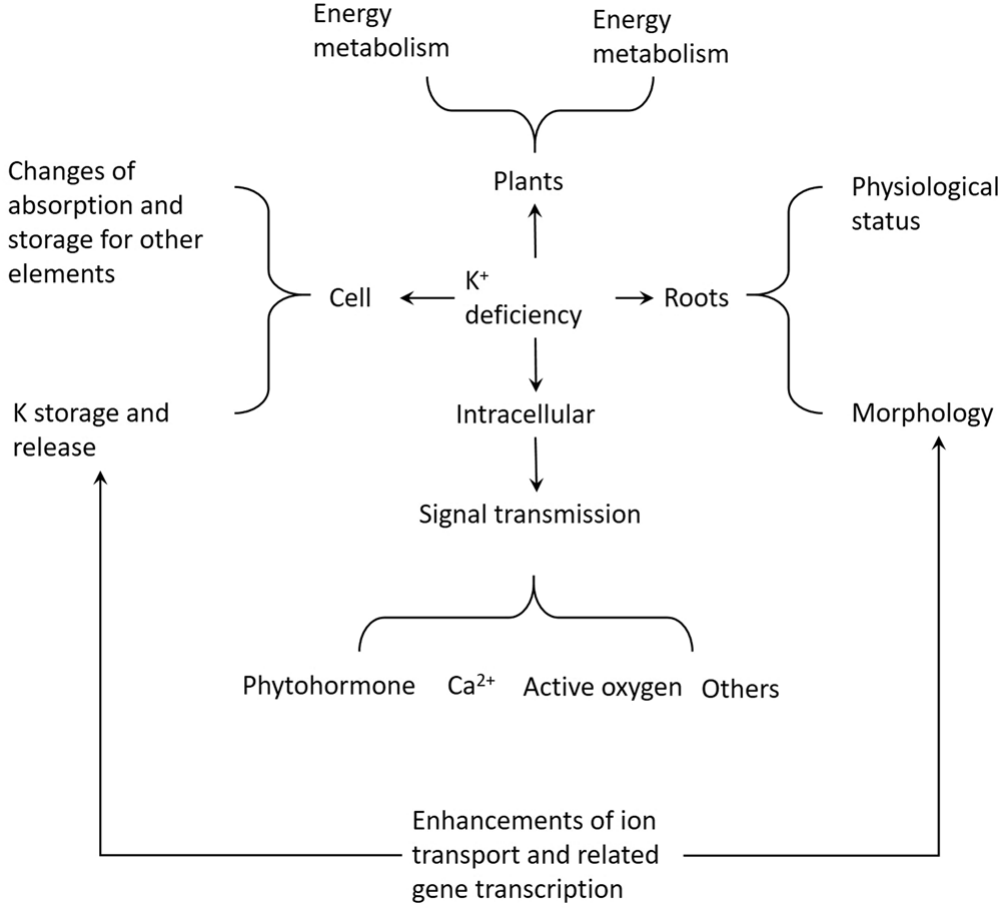
A hypothetical model of low K^+^ stress tolerance mechanism underlying in rice root at seedling stage.

This study employed high-throughput sequencing using mixed materials with different processing times for the analysis of the rice root transcriptome under potassium stress conditions. The results of the study revealed that a large number of genes exhibit clear changes in expression in response to low K^+^ growing conditions. Among the large number of genes differentially expressed in this study, many have unknown functions (Fig. 6), implying that research on the transcriptional changes in rice caused by K^+^ stress is a rich area for future investigations.

## Conclusions

Transcriptome sequencing was applied to perform transcriptomic analysis of mixed rice root samples at different times under K^+^ stress conditions. Physiological experiments showed that low potassium treatment during the seedling phase resulted in increased Superoxide Dismutase (SOD) and Peroxidase (POD) activity and increased MDA content. Transcriptome analysis yielded a total of 805 differentially expressed genes, including genes related to plant hormones, peroxidases, and a series of genes related to the detection and transport of K^+^. The differentially expressed genes were related to root growth and development and included transcription factors from many different gene families, implicating a complex mechanism for the response to low potassium stress conditions in rice. Changes in rice growth and biological pathways under low potassium conditions are highly important for understanding K^+^ detection and transport in rice varieties that are tolerant to low potassium.

## Material and Methods

### Plant materials and growth conditions

A hydroponic experiment was conducted in a plant growth chamber set to a photoperiod of 14 h light/10 h dark. Seeds of Nipponbare rice were sterilized with 2% H_2_O_2_ for 30 min, rinsed with distilled water three times, and then incubated for two days in the dark at 26 °C. The seedlings were cultured and transplanted as previously described^25^. The nutrient solution was prepared according to an established protocol^26^ and contained 2.9 mM NH_4_NO_3_, 0.32 mM NaH_2_PO_4_, 1.0 mM K_2_SO_4_, 1.0 mM CaCl_2_, 1.7 mM MgSO_4_ · 7H_2_O, 9.1 × 10^−3^ mM MnCl_2_ · 4H_2_O, 5.2 × 10^−4^ mM (NH_4_)_6_MoO_24_ · 4H_2_O, 1.8 × 10^−2^ mM H_3_BO_3_, 1.5 × 10^−4^ mM ZnSO_4_ · 7H_2_O, 1.6 × 10^−4^ mM CuSO_4_ · 5H_2_O, and 3.6 × 10^−2^ mM FeCl_3_ · 6H_2_O. The pH value of the culture solution was adjusted to 5.1 using 1 M HCl or NaOH solution as required every two days. Ten days after transplanting the seedlings to the full-strength nutrient solution, rice seedlings were subjected to low-K treatment. The potassium concentration was adjusted to 0.01 mM (low-K treatment) or 1 mM for the control. The experiment was conducted as a split-plot design with three independent replicates.

### RNA-Seq sampling and RNA isolation

For RNA-Seq sampling, seedlings at the three-leaf-stage were exposed to low-K^+^ stress (0.01 mM) for 24 h, 48 h, and 84 h, respectively. The roots of 12 seedlings were collected and pooled together, immediately frozen in liquid nitrogen, and stored at −80 °C. RNA was isolated according to the instructions of the miRNeasy mini kit (QIAGEN, Germany). RNA sample quantity and purity were tested to confirm they met the protocol requirements. Subsequently, equal amounts of total RNA from 24 h, 48 h, and 84 h were mixed and stored.

### RNA extraction

TRIZOL reagent (Invitrogen, USA) was used for RNA extraction per the manufacturer’s instructions. Extracted RNA was stored at −80 °C until it was used for transcriptome sequencing and real-time fluorescent quantitative PCR validation.

### Transcriptome sequencing

Total RNA was treated with 10 U DNaseI (Ambion, USA) at 37 °C, and then mRNA was purified using the Micropoly(A) Purist mRNA purification kit (Ambion). First-strand complementary deoxyribonucleic acid (cDNA) synthesis was performed using *Gsu*I-oligo(dT) (Invitrogen) as the primer for reverse transcription, 10 µg mRNA as template, and Superscript II reverse transcriptase (Invitrogen) for elongation at 42 °C. Next, NaIO_4_ (Sigma, USA) was applied to oxidize 5′ mRNA cap structures and conjugate them to biotin. Dynal M280 magnetic beads (Invitrogen) were used to screen for biotin-conjugated mRNA/cDNA, and cDNA was released by alkaline hydrolysis. Next, DNA ligase (TaKaRa, Japan) was employed to add an adapter to the 5′ end of the first-strand cDNA, and ExTaq polymerase (TaKaRa) was used to synthesize the second strand of cDNA. Finally, the *Gsu*I restriction enzyme was used to cleave the poly(A) tail and the 5′ end adapter. Synthesized cDNA was broken into 300–500-bp fragments using a sonicator (Fisher) and then purified with Ampure beads (Agencourt, USA). The purified cDNA was used to produce a library with the TruSeq^TM^ DNA Sample Prep Kit – Set A (Illumina, USA), followed by amplification with the TruSeq PE Cluster Kit (Illumina). Finally, the sequencing reaction was performed on an Illumina Hiseq 2500.

### Sequencing data processing

Clean reads were obtained using the FASTX-Toolkit software (http://hannonlab.cshl.edu/fastx_toolkit/) and the following data processing procedure and statistical analysis: 1) FASTX Clipper tool was used to remove linker sequences from the reads; 2) FASTX Quality Filter tool was used to remove unconfirmed bases from the reads in the 3′ to 5′ direction until the first confirmed base was reached; 3) After the FASTX Quality Filter removed successive low-quality bases, if the length of clean reads was less than 40 bp, the sequence and its complement were deleted; 4) A local script was used to complement pair end reads (using pair-end sequences).

Clean read mapping was performed with a provided reference genome (http://plants.ensembl.org/Oryza_sativa/Info/Index). Statistical analysis of mapping data was performed as follows. Gene Ontology (GO) analysis was performed using GoPipe^27^. Predicted proteins were first compared with the SwissProt and TrEMBL^28^ databases using the Protein Basic Local Alignment Search Tool and an e-value < 1e-5. Next, the GoPipe program was used to compare results based on gene2go (National Center for Biotechnology Information), and GO data of predicted proteins were obtained.

Predicted proteins were compared using the Kyoto Encyclopedia of Genes and Genomes (KEGG) database with bidirectional BLAST (http://www.genome.jp/tools/kaas/help.html), and the KEGG ORTHOLOGY number of predicted proteins was obtained. Based on the KEGG ORTHOLOGY number, data for the metabolic pathways predicted proteins participated in were obtained.

Statistical analysis of genes with read numbers from two samples was converted into reads per kilobase of transcript per million mapped reads^29^. Finally, the microarray-plot-based method with random sampling model) in the DEGseq program package^30^ was adopted to calculate the difference in expression abundance between the two samples for each gene. False discovery rate values less than 0.001 were considered to be significantly different.

### Construction of protein-protein interaction networks

Medusa software^31^ was used for data display based on the comparison of the protein-protein interaction data from rice (http://arabidopsisreactome.org/download/all_interactions.html).

### QPCR validation

To further evaluate the accuracy of the sequencing data, 30 genes with significant changes in their expression were selected for investigation. Fluorescent quantitative analysis of gene expression from rice roots grown in K^+^ stress conditions and correlation analysis of transcriptome data were conducted. Primers and probes for quantitative analysis used in this study were designed using the online tool at https://www.genscript.com/ssl-bin/app/primer.

### Gene microarray data collection

To understand the pattern of differentially expressed genes in different tissues and environmental conditions more thoroughly, this study conducted correlation analysis using gene microarray data. Analyses of gene microarrays were conducted using the online tool Rice Oligonucleotide Array Database at http://www.ricearray.org with the default parameters.

## Electronic supplementary material

SUPPLEMENTARY MATERIAL

## References

[1] Wang Y, Wu WH (2013). Potassium transport and signaling in higher plants. Annu. Rev. Plant Biol..

[2] Amtmann A, Armengaud P (2009). Effects of N, P, K and S on metabolism: new knowledge gained from multi-level analysis. Curr. Opin. Plant Biol..

[3] Maathuis FJ (2009). Physiological functions of mineral macronutrients. Curr. Opin. Plant Biol..

[4] Rengel Z, Damon PM (2008). Crops and genotypes differ in efficiency of potassium uptake and use. Physiol. Plant..

[5] Römheld V, Kirkby EA (2010). Research on potassium in agriculture: needs and prospects. Plant Soil..

[6] Besford R (1978). Effect of replacing nutrient potassium by sodium on uptake and distribution of sodium in tomato plants. Plant Soil..

[7] Leigh R, Wyn Jones R (1984). A hypothesis relating critical potassium concentrations for growth to the distribution and functions of this ion in the plant cell. New Phytol..

[8] Dobermann A, Cassman KG, Mamaril CP, Sheehy JE (1998). Management of phosphorus, potassium, and sulfur in intensive, irrigated lowland rice. Field Crops Res..

[9] Liu G (2002). Screening indica Rice for K-efficient Genotypes. Acta Agron. Sin..

[10] Liu J, Yang X, Yang Y, Lianghuan WU (2003). Some agronomic and nutritional characteristics for potassium efficient rice genotypes under low potassium stress. Plant Nutr. Fert. Sci..

[11] Yang XE, Liu JX, Wang WM, Ye ZQ, Luo AC (2004). Potassium Internal Use Efficiency Relative to Growth Vigor, Potassium Distribution, and Carbohydrate Allocation in Rice Genotypes. J. Plant Nutr..

[12] Cherel I, Lefoulon C, Boeglin M, Sentenac H (2014). Molecular mechanisms involved in plant adaptation to low K(+) availability. J. Exp. Bot..

[13] Wang Z, Gerstein M, Snyder M (2009). RNA-Seq: a revolutionary tool for transcriptomics. Nat. Rev. Genet..

[14] Marioni JC, Mason CE, Mane SM, Stephens M, Gilad Y (2008). RNA-seq: an assessment of technical reproducibility and comparison with gene expression arrays. Genome Res..

[15] Fatih O, Milos PM (2011). RNA sequencing: advances, challenges and opportunities. Nat. Rev. Genet..

[16] Cloonan N, Grimmond SM (2008). Transcriptome content and dynamics at single-nucleotide resolution. Genome Biol..

[17] Mortazavi A, Williams BA, McCue K, Schaeffer L, Wold B (2008). Mapping and quantifying mammalian transcriptomes by RNA-Seq. Nat. Methods.

[18] Oshlack A, Robinson MD, Young MD (2010). From RNA-seq reads to differential expression results. Genome Biol..

[19] Postnikova OA, Shao J, Nemchinov LG (2013). Analysis of the alfalfa root transcriptome in response to salinity stress. Plant Cell Physiol..

[20] Zeng J (2014). Comparative transcriptome profiling of two Tibetan wild barley genotypes in responses to low potassium. PLoS One.

[21] Yang W (2015). Transcriptome analysis of nitrogen-starvation-responsive genes in rice. BMC Plant Biol..

[22] Yamamoto N (2015). Comprehensive analysis of transcriptome response to salinity stress in the halophytic turf grass Sporobolus virginicus. Front Plant Sci..

[23] Rahman H (2014). Transcriptome analysis of salinity responsiveness in contrasting genotypes of finger millet (*Eleusine coracana* L.) through RNA-sequencing. Plant Mol. Biol..

[24] Wang GY, Lu WY, Chen HN, Zhang XQ, Xue DW (2015). Seedling Screening of Rice Germplasm Resources with Low Potassium Tolerance. J. Hangzhou Norm. Univ..

[25] Fang Y (2015). Identification of quantitative trait loci associated with tolerance to low potassium and related ions concentrations at seedling stage in rice (*Oryza sativa* L.). Plant Growth Regul..

[26] Yoshida, S., Forno, D. A. Cock, J. H. & Gomez, K. A. Routine procedures for growing rice plants in culture solution in *Laboratory manual for physiological studies of rice* (ed. Yoshida, S., Forno, D. A. Cock, J. H. & Gomez, K. A.) 61 (International Rice Research Institute, 1976).

[27] Chen Z (2005). GoPipe: Streamlined gene ontology annotation for batch anonymous sequences with statistics. Prog. Biochem. Biophys..

[28] Bairoch A, Apweiler R (2000). The SWISS-PROT protein sequence database and its supplement TrEMBL in 2000. Nucleic Acids Res..

[29] Wagner GP, Kin K, Lynch VJ (2012). Measurement of mRNA abundance using RNA-Seq data: RPKM measure is inconsistent among samples. Theory Biosci..

[30] Wang L, Feng Z, Wang X, Wang X, Zhang X (2009). DEGseq: an R package for identifying differentially expressed genes from RNA-seq data. Bioinformatics.

[31] Hooper SD, Bork P (2005). Medusa: a simple tool for interaction graph analysis. Bioinformatics.

[32] Ribot C (2008). Susceptibility of rice to the blast fungus, Magnaporthe grisea. J. Plant Physiol..

[33] Jain M (2007). F-box proteins in rice. Genome-wide analysis, classification, temporal and spatial gene expression during panicle and seed development, and regulation by light and abiotic stress. Plant Physiol.

[34] Munson, R. D. Potassium in Agriculture (Soil Science Society of America Madison, Wisconsin, USA, 1985).

[35] Demidchik V (2014). Mechanisms and physiological roles of K+ efflux from root cells. J. Plant Physiol..

[36] Armengaud P (2009). Multilevel analysis of primary metabolism provides new insights into the role of potassium nutrition for glycolysis and nitrogen assimilation in Arabidopsis roots. Plant Physiol..

[37] Hafsi C, Debez A, Abdelly C (2014). Potassium deficiency in plants: effects and signaling cascades. Acta Physiol. Plant..

[38] Joo J, Lee YH, Kim YK, Nahm BH, Song SI (2013). Abiotic stress responsive rice ASR1 and ASR3 exhibit different tissue-dependent sugar and hormone-sensitivities. Mol. Cells.

[39] Arenhart RA (2013). Involvement of ASR genes in aluminium tolerance mechanisms in rice. Plant Cell Environ..

[40] Tian X (2015). Characterization and Functional Analysis of Pyrabactin Resistance-Like Abscisic Acid Receptor Family in Rice. Rice (NY).

[41] Chen G (2015). Rice potassium transporter OsHAK1 is essential for maintaining potassium-mediated growth and functions in salt tolerance over low and high potassium concentration ranges. Plant Cell Environ.

[42] Banuelos MA, Garciadeblas B, Cubero B, Rodriguez-Navarro A (2002). Inventory and functional characterization of the HAK potassium transporters of rice. Plant Physiol..

[43] Horie T (2001). Two types of HKT transporters with different properties of Na+ and K+ transport in *Oryza sativa*. Plant J..

[44] Fuchs I, Stolzle S, Ivashikina N, Hedrich R (2005). Rice K+ uptake channel OsAKT1 is sensitive to salt stress. Planta.

[45] Lan WZ (2010). A rice high-affinity potassium transporter (HKT) conceals a calcium-permeable cation channel. Proc Natl. Acad. Sci. USA.

[46] Ma TL, Wu WH, Wang Y (2012). Transcriptome analysis of rice root responses to potassium deficiency. BMC Plant Biol..

[47] Takehisa H, Sato Y, Antonio B, Nagamura Y (2015). Coexpression Network Analysis of Macronutrient Deficiency Response Genes in Rice. Rice (N Y).

[48] Li J (2014). The Os-AKT1 channel is critical for K+ uptake in rice roots and is modulated by the rice CBL1-CIPK23 complex. Plant Cell.

