# Supplementary material for: Transcriptome Analysis of Rice Seedling Roots in Response to Potassium Deficiency
Source: Sci Rep. 2017 Jul 17;7:5523. doi: 10.1038/s41598-017-05887-9 (PMC5514036; doi:10.1038/s41598-017-05887-9)

Transcriptome Analysis of Rice Seedling Roots in Response to Potassium Deficiency

Xiaoqin Zhang[1, #], Hua Jiang[2, #], Hua Wang[2], Jun Cui[1], Jiahui Wang[1], Jiang Hu[3], Longbiao Guo[3], Qian Qian[3]*, Dawei Xue[1,]*

[1] College of Life and Environmental Sciences, Hangzhou Normal University, Hangzhou, China

[2] State Key Laboratory Breeding Base for Zhejiang Sustainable Pest and Disease Control, Zhejiang Academy of Agricultural Science, Hangzhou, China

[3] State Key Laboratory of Rice Biology, China National Rice Research Institute, Hangzhou, China

[#] These authors contributed equally to this work. * Correspondence should be addressed to Q.Q. (email: qianqian188@hotmail.com) or D.X. (email: dwxue@hznu.edu.cn).

SUPPLEMENTARY MATERIAL

Table S1 Summary of the sequencing data of the transcriptome sequencing

Figure S2 Length of genes identified from the transcriptome sequencing experiment

Table S3 The sum results of map to gene in this transcriptome sequencing experiment

Table S4 The list of length of genes from the analysis of the transcriptome sequencing

Figure S5 The coverage results from the analysis of the transcriptome sequencing

Table S6 List of all differentially expressed genes identified in this study

Table S7 List of primers used in QPCR analysis

Table S8 Pfam ID of differentially expressed genes

Figure S9 Protein network of differentially expressed genes

Figure S10 Expression analysis of differentially expressed genes from different tissue using microarray data

Figure S11 Heat map of differentially expressed genes during different growth stages in rice using microarray data

Figure S12 Expression analysis of differentially expressed genes in two particular microarray GSE6901 and GSE7256

Figure S13 Gene function classification of differentially expressed genes identified in this study

Figure S14 Effect of low K^+ stress at seedling stage on activities of SOD (A), activities of POD (B) and MDA contents (C) of Nipponbare for 24 h, 48 h, and 84 h. Error bars represent ± SE (n=3).

SUPPLEMENTARY METHOD

Cellular antioxidant enzyme activities and malondialdehyde content. For determining enzyme activities and malondialdehyde (MDA) content, rice roots were homogenized in 8 mL of 50 mM sodium phosphate buffer (pH 7.8) using a prechilled mortar and pestle. The mixture was then centrifuged at 10,000 × *g* for 15 min at 4°C. The supernatant was used for the enzyme activity assays. Superoxide dismutase (SOD) and peroxidase (POD) activities, and MDA content, were measured according to an established protocol [1].

1 Zhang, X. *et al.* Measuring the damage of heavy metal cadmium in rice seedlings by SRAP analysis combined with physiological and biochemical parameters. *Journal of the science of food and agriculture* **95**, 2292-2298, doi:10.1002/jsfa.6949 (2015).

Table S1 Summary of the sequencing data of the transcriptome sequencing

**Sample**	**ck**	**wk**
Raw Reads (pair)	11432307	15640647
Clean reads(pair)	10972168	15120257
Average length(bp)	2*100	2*100
Raw data	2.28G	3.12G
Clean data	2.08G	3.01G
Read 1 Q20	98.28%	98.34%
Read 1 Q30	92.40%	92.82%
Read 1 GC content	46.04%	44.14%
Read 2 Q20	98.76%	98.71%
Read 2 Q30	93.45%	93.49%
Read 2 GC content	45.90%	44.03%

Figure S2 Length of genes identified from the transcriptome sequencing experiment

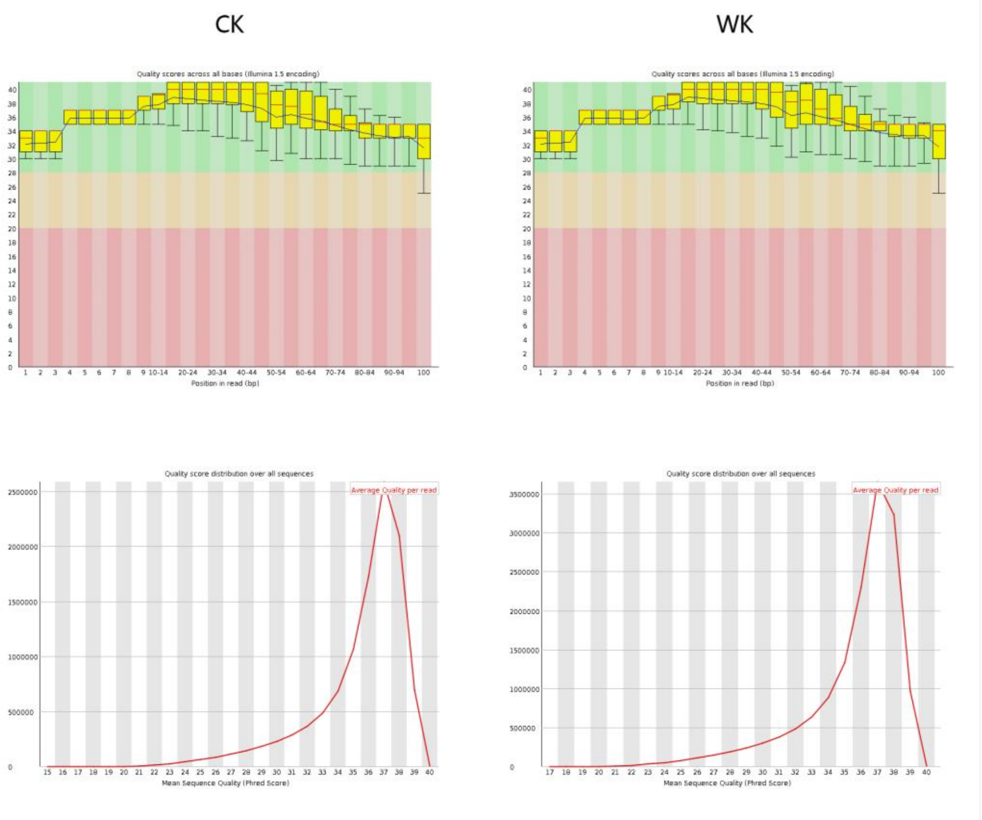

Table S3 The sum results of map to gene in this transcriptome sequencing experiment

Map to gene	ck		wk	
	Reads number	percentage	Reads number	percentage
clean reads	10972168	100%	15120257	100%
Total mapped reads	2564803	23.38%	3236096	21.40%
Total unmapped reads	8407365	76.62%	11884161	78.60%
Unique_match	1390948	12.68%	1796520	11.88%
Multi-position match	1173855	10.70%	1439576	9.52%
Perfect match	2290442	20.88%	2898012	19.17%
<=5bp mismatch	268003	2.44%	329640	2.18%

Table S4 The list of length of genes from the analysis of the transcriptome sequencing

Lehgth of genes	Count
200~300	460
300~400	744
400~500	1154
500~600	1665
600~700	2072
700~800	1969
800~900	1884
900~1000	2062
1000~1200	4323
1200~1400	4308
1400~1600	4222
1600~1800	3660
1800~2000	2771
2000~2500	4618
2500~3000	2760
3000~3500	1653
3500~4000	881
>4000	835

Figure S5 The coverage results from the analysis of the transcriptome sequencing

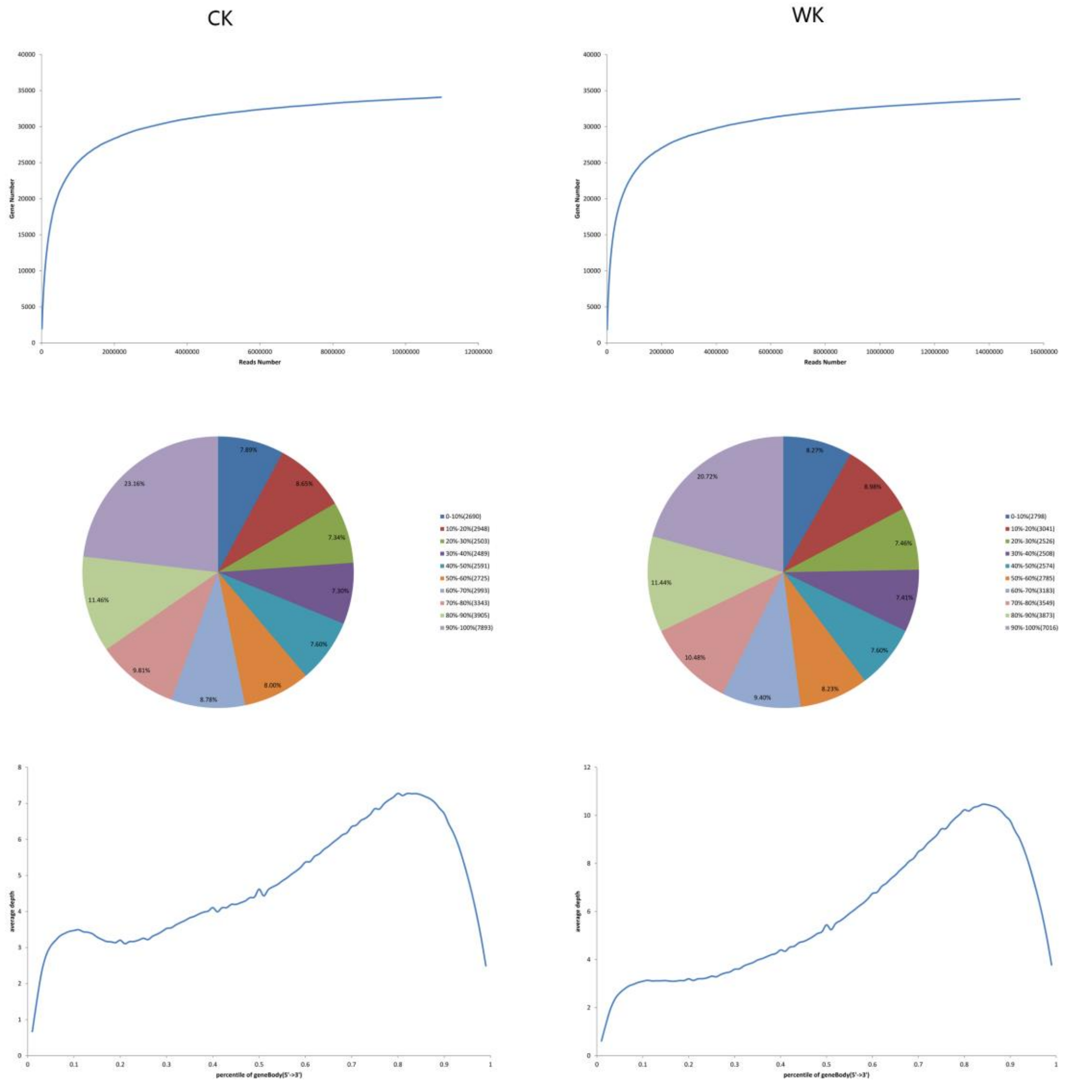

Table S6 List of all differentially expressed genes identified in this study

#Gene	RAP-DB Description	Length	Function	read_A	RPKM_A	read_B	RPKM_B	Ration	log2(Fold_change) normalized	q-value (Benjamini et al. 1995)	p_value
OS02T0814300-01	Cytochrome c, monohaem domain containing protein.	614	gi|47847871|dbj|BAD21664.1| unknown protein	15	9.525085	1	0.503281	18.92559	4.242297	0.000723	4.03E-005

OS05T0428400-00	Rossmann-like alpha/beta/alpha sandwich fold domain containing protein.	1132	gi|46391138|gb|AAS90665.1| hypothetical protein	26	8.955151	7	1.910866	94.686436	2.228491	0.000969	5.69E-05
OS10T0191300-01	Similar to PR-1a pathogenesis related protein (Hv-1a) precursor.	698	gi|22138454|gb|AAM93438.1| putative type-1	643	359.1712	299	132.3715	2.71333	1.44008	4.70E-47	9.26E-5

			pathogenesis-related protein					58			0
OS10T0573700-01	Similar to Mitochondrial carnitine/acylcarnitine carrier-like protein (A BOUT DE SOUFFLE) (Carnitine/acylcarnitine translocase-like protein) (CAC-like protein).	1229	gi|12643054|gb|AAK00443.1|AC060755_13 putative carnitine/acylcarnitine translocase	48	15.22774	16	4.022969	3.785198	1.920369	1.86E-05	6.64E-07
OS03T0	Similar to Physical impedance induced protein.	77	gi|39748092|gb|AAR3	200	100.101	101	40.06476	2.4	1.321051	8.32E-13	9.5

103100-01		9	0139.1| lipid transfer protein-like protein					98481			3E-15
OS05T0526200-01	Similar to Calcium homeostasis regulator CHoR1.	1215	gi|52353494|gb|AAU44060.1| unknown protein	41	13.1569	17	4.323657	3.043003	1.605496	0.000865	4.97E-005
OS0	Similar to Histone H4.	6	TPA:	238	138.0873	149	68.51657	2.	1.0110	5.41E-	8.

5T0466600-01		72	histone H4.3					015385	56	10	89E-12
OS04T0689000-01	Similar to Peroxidase (EC 1.11.1.7).	1239	gi|38345511|emb|CAE01795.2| OSJNBa0039K24.14	352	110.7688	153	38.15915	2.90281	1.53745	2.76E-28	1.06E-30
OS0	Peroxidase (EC 1.11.1.7).	1	gi|3834550	292	76.66593	146	30.38121	2.	1.3354	5.82E-	3.

4T0688100-01		485	6|emb|CAE01785.2| OSJNBa0039K24.4					523466	06	19	83E-21
OS06T0232300-01	Similar to Auxin transport protein REH1.	24469	gi|51535183|dbj|BAD38156.1| putative auxin transporter	154	24.31899	81	10.13777	2.39885	1.262343	2.99E-09	5.35E-11
OS0	Protein of unknown function DUF26	1	gi|3834698	135	47.93772	76	21.38897	2.	1.1642	3.36E-	8.

4T0<br>322<br>100-<br>01	domain containing protein.	0<br>9<br>8	5|emb|CAD<br>40285.2| OSJNBb00<br>62H02.10					2<br>4<br>1<br>2<br>3<br>6	94	07	2<br>7<br>E-<br>0<br>9
OS0<br>3T0<br>694<br>700-<br>01	Similar to PIF-like orf1.	5<br>8<br>0	gi|1087105<br>35|gb|ABF9<br>8330.1| expressed<br>protein	106	71.2564	57	30.36865	2.<br>3<br>4<br>6<br>3<br>8	1.2304<br>37	3.19E-<br>06	9.<br>5<br>5<br>E-<br>0<br>8
OS0	Hypothetical protein.	1	gi|3439363	161	39.78001	69	13.51203	2.	1.5577	3.43E-	3.

7T0582800-01		578	4|dbj|BAC83314.1| unknown protein					9440443	99	13	68E-15
OS03T0673700-01	Similar to predicted protein.	4889	gi|215693129|dbj|BAG88511.1| unnamed protein product	41	32.69046	12	7.583173	4.31092	2.107996	2.82E-05	1.05E-06
OS0	Hypothetical conserved gene.	2	gi|5185436	469	89.76929	271	41.11089	2.	1.1267	2.70E-	1.

5T0128200-01		037	3|gb|AAU10743.1| putative finger transcription factor					183589	01	23	28E-25
OS07T0297400-01	Conserved hypothetical protein.	17229	hypothetical protein OsJ_09028	113	25.48176	61	10.90218	2.337308	1.224848	1.45E-06	4.05E-08

OS12T0438000-02	Similar to Histone H2A.	823	gi|77555213|gb|ABA98009.1| Histone H2A, putative, expressed	98	46.42717	53	19.90007	2.333015	1.222196	1.01E-05	3.35E-07
OS07T0112700-01	Cupredoxin domain containing protein.	858	gi|38175738|dbj|BAC22293.2| blue copper-binding	132	59.98361	54	19.44845	3.084233	1.624913	1.55E-11	2.09E-13

			protein-like					6			
OS0<br>3T0<br>749<br>300-<br>03	Similar to Exoglucanase.	1<br>6<br>7<br>5	gi|1808767<br>4|gb|AAL58<br>966.1|AC09<br>1811_15<br>putative<br>exoglucana<br>se<br>precursor	112	26.07049	54	9.962253	2.<br>6<br>1<br>6<br>9<br>2<br>7	1.3878<br>74	8.01E-<br>08	1.<br>7<br>5<br>E-<br>0<br>9
OS1<br>1T0<br>474<br>566-	Hypothetical conserved gene.	5<br>7<br>8	expressed<br>protein	356	240.142	178	95.16358	2.<br>5<br>2<br>3	1.3354<br>06	4.21E-<br>23	2.<br>0<br>1<br>E-

01								466			25
OS04T0253000-01	Similar to Histone H1.	890	gi|113563891|dbj|BAF14234.1| Os04g0253000, partial	222	97.25433	113	39.2344	2.4788802	1.309643	5.25E-14	5.24E-16
OS10T0169	Conserved hypothetical protein.	4991	expressed protein	265	210.4313	155	97.55034	2.115	1.109131	7.16E-13	8.114

200-01								7156			E-15
OS05T0363200-01	UDP-glucuronic acid decarboxylase.	17331	gi|49387469|dbj|BAD24936.1| UDP-glucuronic acid decarboxylase	414	93.25009	260	46.41463	2.009067	1.006526	3.97E-17	3.06E-19
OS01T0	Conserved hypothetical protein.	60	gi|15623980|dbj|BAB6	126	80.93341	8	4.072676	19.	4.312686	1.84E-30	6.4

510200-01		7	8035.1| unknown protein					87229			5E-33
OS03T0204300-01	Conserved hypothetical protein.	1661	gi|255674292|dbj|BAH92034.1|Os03g0204201	234	54.9278	97	18.04599	3.043768	1.605858	5.43E-20	3.21E-22
OS1	Similar to Tfm5 protein.	7	gi|1014065	320	177.7292	164	72.19138	2.	1.2997	6.47E-	3.

0T0552800-01		02	1|gb|AAG13487.1|AC026758_24 putative lipid transfer protein					461918	82	20	84E-22
OS11T0258900-01	Conserved hypothetical protein.	1882	hypothetical protein	75	15.53773	31	5.090034	3.052257	1.610029	1.38E-06	3.884E-08

								9			
OS12T0150100-01	Similar to 60S ribosomal protein L26A.	672	gi|77553015|gb|ABA95811.1| 60S ribosomal protein L26-1, putative, expressed	585	339.4162	357	164.1639	2.067545	1.047919	7.39E-26	3.14E-28
OS04T0659200-	Hypothetical protein.	2487	gi|38346411|emb|CAE54576.1| OSJNBa00	99	15.52049	46	5.715584	2.7155	1.441201	2.44E-07	5.80E-

01			11F23.17					468			09
OS04T0320700-01	Similar to Glucosyltransferase (Fragment).	1673	gi|38344780|emb|CAE01506.2| OSJNBb0026L04.11	78	18.17794	47	8.681215	2.093939	1.06622	0.000793	4.500E-05
OS10T0556	Similar to beta-expansin EXPB4.	1447	gi|14165334|gb|AAK55466.1|AC06	47	12.44904	19	3.988636	3.12	1.642068	0.000216	1.001

100- 01		2	9300_21 beta-expan sin (EXPB4)					1 1 2 8			E- 0 5
OS0 1T0 797 600- 01	AP2/ERF family protein, ERF-associated EAR-motif-containing repressor, Abiotic stress response, Stress signaling	1 0 6 1	gi|9309342| dbj|BAB032 48.1| ethylene responsive element binding factor3	93	34.1754	48	13.97991	2. 4 4 4 6 0 7	1.2896 03	6.73E- 06	2. 1 7 E- 0 7
OS0	FAS1 domain domain containing	1	hypothetical	57	20.61589	23	6.59307	3.	1.6447	3.03E-	1.

8T0<br>502<br>400-<br>01	protein.	0<br>7<br>8	protein<br>OsI_29781					1<br>2<br>6<br>9<br>0<br>3	34	05	1<br>3<br>E-<br>0<br>6
OS0<br>2T0<br>236<br>600-<br>01	Peroxidase P7 (EC 1.11.1.7) (TP7).	1<br>5<br>3<br>3	gi|5570092<br>1|tpe|CAH6<br>9269.1| TPA: class<br>III<br>peroxidase<br>27	51	12.97102	23	4.636223	2.<br>7<br>9<br>7<br>7<br>5<br>5	1.4842<br>7	0.0003<br>79	1.<br>9<br>4<br>E-<br>0<br>5

			precursor								
OS10T0529800-00	Similar to Tau class GST protein 4.	8333	gi|22213179|gb|AAM94519.1| putative glutathione S-transferase	320	149.779	114	42.29007	3.541706	1.824444	8.29E-33	2.60E-35
OS09T0483150-00	Hypothetical gene.	3886	unknown	41	41.41356	17	13.60944	3.0430	1.605496	0.000865	4.97E-0

								03			5
OS01T0294700-01	Haem peroxidase, plant/fungal/bacterial family protein.	1331	gi|9909174|dbj|BAB12033.1| putative peroxidase	374	109.5568	207	48.05858	2.279652	1.188814	3.09E-20	1.80E-22
OS11T0151300-	Similar to 60S ribosomal protein L26-1.	786	gi|125533419|gb|EAY79967.1| hypothetical	332	164.6878	201	79.02275	2.084	1.059394	6.13E-15	5.57E-

01			protein OsI_35131					056			17
OS06T0157125-00	Hypothetical protein.	861	no hit	119	53.88772	60	21.5341	2.502437	1.323334	9.89E-08	2.220E-09
OS02T0625	Similar to PEPC kinase.	140	phosphoenolpyruvate carboxylase	47	13.07061	18	3.967374	3.29	1.72007	0.000117	5.06

300-00		2	kinase					4524			E-06
OS01T0839900-01	Thaumatin, pathogenesis-related family protein.	1001	gi|15623832|dbj|BAB67891.1| putative thaumatin-like cytokinin-binding protein	182	70.88973	89	27.4748	2.580173	1.367468	2.72E-12	3.28E-14

OS04T0583600-01	Similar to Histone H4.	594	TPA: histone H4.3	192	126.0262	86	44.73945	2.816892	1.494104	8.74E-15	8.12E-17
OS10T0551401-00	Conserved hypothetical protein.	2261	predicted protein	54	9.311919	19	2.596759	3.585597	1.842366	8.88E-06	2.93E-07

OS04T0517000-01	Similar to OSIGBa0145M07.3 protein.	923	gi|125549031|gb|EAY94853.1| hypothetical protein OsI_16649	52	21.96583	16	5.356695	74.1000632	2.035846	2.60E-06	7.577E-008
OS07T0119400-01	Similar to Pectinesterase like protein.	19963	gi|33146556|dbj|BAC79733.1| putative pollen-speci	87	17.28005	50	7.870971	2.19954	1.134494	0.000133	5.887E-0

			fic protein NTP303 precursor					15			6
OS03T0101000-01	Similar to AMP binding protein.	835	gi|125542031|gb|EAY88170.1| hypothetical protein OsI_09611	60	28.0163	29	10.73223	2.610482	1.384316	0.000232	1.10E-05
OS02T0749	Hypothetical protein.	4467	no hit	60	50.09338	14	9.263813	5.440	2.434942	6.18E-09	1.14

400-01								7426			E-10
OS08T0462900-01	Protein of unknown function DUF716 family protein.	11665	gi|42409056|dbj|BAD10308.1| plant viral-response family protein-like	524	175.3684	285	75.59578	2.319817	1.214011	2.24E-29	8.20E-32
OS03T0	Cupredoxin domain containing protein.	76	gi|113547219|dbj|BAF	44	22.33765	17	6.840161	3.2	1.707375	0.000237	1.11

115000-01		8	10662.1| Os03g0115000, partial					65661			3E-05
OS04T0462900-05	Similar to prohibitin2.	1179	B1358B12.15	83	27.44797	34	8.911354	3.080112	1.622983	2.50E-07	5.988E-09
OS0	Similar to predicted protein.	7	expressed	88	45.32447	55	22.4515	2.	1.0134	0.0005	3.

3T0439800-02		57	protein, having alternate splicing products					018772	78	92	22E-05
OS01T0357100-02	Similar to Ferredoxin-nitrite reductase.	2137	putative ferredoxin--nitrite reductase, chloroplast precursor	533	97.24531	157	22.7025	4.2834662	2.098777	4.60E-66	5.38E-69

OS01T0256500-02	Similar to ZnI.	805	gi|124244495|gb|ABM92337.1| zinc inducible protein	16188	7840.492	5663	2173.849	3.606673 3	1.850693	0	0
OS11T0169200-00	Amino acid transporter, transmembrane domain containing protein.	1443	gi|62701938|gb|AAX93011.1| probable AUX1-like permease,	23	6.214519	5	1.070736	5.880397	2.53704	0.000776	4.39E-05

			10674-8589					1			
OS01T0278950-00	Similar to predicted protein.	2397	putative subtilisin-like protein	68	11.06081	32	4.125348	2.681182	1.422869	4.52E-05	1.76E-06
OS09T0365100-01	Hypothetical conserved gene.	747	no hit	77	40.18982	45	18.61532	2.1589	1.11034	0.000522	2.80E-00

								65			5
OS09T0367900-02	Hypothetical conserved gene.	8443	gi|50725951|dbj|BAD33479.1| unknown protein	238	110.0767	111	40.68872	2.7053337	1.435808	2.93E-17	2.21E-19
OS09T0392666-	Conserved hypothetical protein.	1070	hypothetical protein	197	71.78413	63	18.1943	3.9945	1.980178	1.75E-22	8.79E-

01								418			25
OS05T0199100-01	Similar to NDR1/HIN1-Like protein 2.	12268	gi|125551167|gb|EAY96876.1| hypothetical protein OsI_18799	100	30.7487	55	13.40362	2.29406	1.197903	1.16E-05	3.91E-07
OS03T0102500-	Similar to Beta-expansin precursor (Fragment).	10117	gi|108705702|gb|ABF93497.1| Beta-expan	56	21.46906	20	6.076978	3.5532	1.820833	6.86E-06	2.22E-

01			sin 1a precursor, putative, expressed					852			07
OS06T0133200-01	Conserved hypothetical protein.	626	gi|55296162|dbj|BAD67880.1| unknown protein	57	35.50148	18	8.885396	3.995487	1.998371	9.86E-07	2.67E-08
OS09T0	Similar to Chlorophyll a-b binding protein, chloroplast precursor (LHCII	12	gi|50252362|dbj|BAD2	219	69.19504	109	27.29543	2.5	1.342009	2.42E-14	2.3

346500-04	type I CAB) (LHCP).	34	8469.1| putative chlorophyll a-b binding protein, chloroplast precursor (LHCII type I CAB) (LHCP)					35041			3E-16
OS12T0454	Cupredoxin domain containing protein.	836	Plastocyanin-like domain	55	25.65089	16	5.91415	4.333	2.116766	5.56E-07	1.43

600-00			containing protein, expressed					7206			E-08
OS03T0188500-01	Glutelin family protein.	629	gi|113547689|dbj|BAF11132.1| Os03g0188500	71	44.01024	27	13.26453	3.31789	1.730266	6.86E-07	1.80E-08
OS05T0311	Hypothetical protein.	523	no hit	43	32.05625	11	6.499345	4.93	2.30224	4.19E-06	1.29

550-00								2228			E-07
OS01T0860601-01	Similar to Ferredoxin, root R-B1.	910	gi|56784805|dbj|BAD82026.1| putative ferredoxin	123	52.69989	73	24.78906	2.125933	1.088096	5.97E-06	1.90E-07
OS01T0	Similar to 60S ribosomal protein L26B.	49	gi|54290308|dbj|BAD6	283	221.122	169	104.6562	2.1	1.079185	3.89E-13	4.2

140500-01		9	1308.1| putative ribosomal protein L26					12842			0E-15
OS03T0306800-01	Similar to CP12 (Fragment).	622	gi|108707737|gb|ABF95532.1| CP12, putative, expressed	118	73.96693	39	19.3755	3.81755	1.932647	4.51E-13	4.94E-15
OS11T0	Similar to RPT2-like protein.	20	gi|77548377|gb|ABA91	51	9.752118	16	2.42483	4.0	2.007832	4.24E-06	1.3

118<br>300-<br>01		3<br>9	174.1| RPT2,<br>putative,<br>expressed					2<br>1<br>7<br>7<br>3			1<br>E-<br>0<br>7
OS0<br>3T0<br>749<br>300-<br>02	Similar to Exoglucanase precursor.	2<br>1<br>1<br>4<br>2	gi|1808767<br>4|gb|AAL58<br>966.1|AC09<br>1811_15<br>putative<br>exoglucana<br>se<br>precursor	139	25.30121	68	9.809978	2.<br>5<br>7<br>9<br>1<br>3	1.3668<br>85	1.95E-<br>09	3.<br>4<br>0<br>E-<br>1<br>1

OS01T0765600-01	EF-Hand type domain containing protein.	1112	gi|15289907|dbj|BAB63602.1| calmodulin-like protein	443	155.3263	254	70.58421	2.200581	1.137885	2.33E-22	1.19E-24
OS01T0878800-01	Similar to 4,5-DOPA dioxygenase extradiol.	1244	gi|113534513|dbj|BAF06896.1| Os01g0878800	124	38.86398	54	13.4138	2.89731	1.534715	5.27E-10	8.64E-12

OS03T0355700-00	Hypothetical conserved gene.	13557	gi|108708215|gb|ABF96010.1| IQ calmodulin-binding motif family protein, expressed	27	7.757645	5	1.138594	26.8133557	2.768366	8.34E-05	3.47E-06
OS02T0689900-	TGF-beta receptor, type I/II extracellular region family protein.	2237	gi|41052810|dbj|BAD07678.1| putative	431	75.12029	188	25.96991	2.892	1.532362	1.64E-34	4.88E-

01			peptide transporter					589			37
OS03T0757950-00	Hypothetical protein.	1447	hypothetical protein BthaB_22716	92	24.78936	56	11.95909	2.0728847	1.051613	0.000245	1.118E-05
OS08T0129	BTB/POZ-like domain containing protein.	1553	putative spop	129	32.68113	60	12.04734	2.771	1.439743	1.79E-09	3.111

300-00		9						2725			E-11
OS01T0179600-01	UDP-glucuronosyl/UDP-glucosyltransferase family protein.	15998	gi|215769461|dbj|BAH01690.1| unnamed protein product	38	9.27156	11	2.127132	4.3587133	2.123902	5.84E-05	2.33E-06
OS08T0	Similar to WRKY transcription factor 25.	10	TPA_inf: WRKY	172	61.58097	99	28.09221	2.1	1.132315	1.05E-08	2.0

235800-00		89	transcription factor 25					92101			0E-10
OS08T0427700-00	Histone H2A.	6990	gi|29837243|dbj|BAC75621.1| putative histone H2A	87	49.16048	50	22.39234	2.195415	1.134494	0.000133	5.87E-06
OS0	Similar to SHI.	1	gi|5379288	25	8.439253	5	1.337724	6.	2.6573	0.0002	1.

6T0712600-00		155	7|dbj|BAD54064.1| putative LRP1					308664	34	57	24E-005
OS09T0433600-01	Similar to Histone H4.	469	TPA: histone H4.3	114	94.77155	67	44.1449	2.1446829	1.102207	1.19E-05	4.03E-07

OS04T0684900-01	Component of the CCR4-NOTcomplex, Deadenylase, Deadenylation (poly(A) tail shortening), Development and stress response	10991	gi|113565884|dbj|BAF16227.1| Os04g0684900	63	22.35058	26	7.310621	3.05727276	1.612247	1.33E-05	4.56E-07
OS02T0116750-00	Hypothetical protein.	11985	no hit	49	9.624575	18	2.802145	3.434471	1.780191	4.68E-05	1.83E-06

								7			
OS03T0738600-01	Lipoxygenase, Seed germination and longevity	2927	gi|31126788|gb|AAP44707.1| lipoxygenase L-2; lipoxygenase	64	8.525174	33	3.483933	2.446997	1.291012	0.000333	1.68E-05
OS10T0114400-01	Protein kinase, catalytic domain domain containing protein.	37781	gi|255679171|dbj|BAF25961.2| Os10g0114400, partial	80	8.249532	35	2.860487	2.8839	1.528051	1.64E-06	4.59E-0

								61			8
OS02T0653200-01	Cupredoxin domain containing protein.	694	putative uclacyanin 3	212	119.1029	110	48.97921	2.431703	1.281967	5.98E-13	6.65E-15
OS11T0703900-	Similar to Heat shock protein 70.	529	Heat shock cognate 70 kDa protein,	1310	965.5207	815	476.0807	2.028	1.020101	1.11E-55	1.60E-

02			putative, expressed					061			58
OS02T0611800-01	Similar to Hydroxyanthranilate hydroxycinnamoyltransferase 3.	17116	gi|47497614|dbj|BAD19683.1| putative hydroxyanthranilate hydroxycinnamoyltransferase 3	535	121.5577	321	57.80513	2.1028888	1.072372	1.50E-24	6.855E-27
OS0	Hypothetical protein.	9	gi|1136317	2048	819.8171	1244	394.6754	2.	1.0546	2.80E-	1.

9T0484200-01		74	46|dbj|BAF25427.1| Os09g0484200					077194	36	92	98E-95
OS03T0105633-00	Hypothetical protein.	1348	PREDICTED: uncharacterized protein LOC100947132	100	28.92385	50	11.46196	2.523466	1.3354 06	1.22E-06	3.34E-08

OS03T0799050-00	Hypothetical protein.	502	no hit	54	41.94073	14	8.617929	4.8666884	2.282939	1.71E-07	3.933E-09
OS05T0447700-01	Tetratricopeptide-like helical domain containing protein.	20998	gi|51854380|gb|AAU10760.1| unknown protein	40	7.433622	15	2.209349	3.336462	1.750444	0.000394	2.04E-05

OS03T0183300-01	Pathogenesis-related transcriptional factor and ERF domain containing protein.	1192	gi|56567587|gb|AAV98703.1| BTH-induced ERF transcriptional factor 4	429	140.3224	257	66.62473	12.106161	1.074616	9.85E-20	5.94E-22
OS07T0580800-01	Hypothetical protein.	14665	gi|222637341|gb|EEE67473.1| hypothetical protein	37	9.847139	11	2.320244	4.2440	2.085428	9.67E-05	4.10E-0

			OsJ_24883					1			6
OS01T0950300-01	Conserved hypothetical protein.	8661	TPA: hypothetical protein ZEAMMB73_129180, partial	65	29.43447	34	12.20266	2.412136	1.270311	0.000362	1.84E-05
OS05T0539800-02	Protein of unknown function DUF778 family protein.	1070	gi|55733902|gb|AAV59409.1| unknown protein	84	30.60846	38	10.97434	2.78890	1.479796	1.61E-06	4.50E-0

								93			8
OS05T0135200-01	Plant peroxidase domain containing protein.	1306	gi|55701005|tpe|CAH69311.1| TPA: class III peroxidase 69 precursor	41	12.24015	13	3.075947	3.979311	1.992519	6.22E-05	2.500E-06
OS08T0554	Zinc finger, C2H2-type domain containing protein.	245	gi|45736048|dbj|BAD13075.1|	68	10.78193	31	3.895666	2.76	1.468673	2.68E-05	9.888

400-02		9	putative zinc finger protein					7672			E-07
OS09T0403300-00	Similar to cytochrome P450.	17551	gi|51090610|dbj|BAD36323.1| putative cytochrome P450 monooxygenase CYP72A5	103	22.93491	55	9.706332	2.3628881	1.240547	3.92E-06	1.20E-07

OS06T0127250-00	Hypothetical protein.	1805	no hit	173	37.36929	103	17.6335	2.119221	1.083534	3.55E-08	7.43E-10
OS01T0851600-01	3-oxo-5-alpha-steroid 4-dehydrogenase, C-terminal domain containing protein.	1111	gi|20805184|dbj|BAB92853.1| putative steroid 5alpha-redu	55	19.30166	28	7.78794	2.47840	1.309411	0.000942	5.48E-05

			ctase					4			
OS04T0677033-01	Similar to H0402C08.3 protein.	5336	H0402C08.3	39	28.36912	15	8.647789	3.2805055	1.713918	0.000623	3.39E-05
OS12T0100200-00	Conserved hypothetical protein.	25596	gi|77548278|gb|ABA91075.1| expressed protein	160	24.03042	45	5.356566	4.44861	2.165481	1.79E-20	1.02E-22

								6<br>1<br>3.			2
OS0<br>6T0<br>686<br>400-<br>01	Bifunctional inhibitor/plant lipid transfer protein/seed storage domain containing protein.	6<br>6<br>9	gi|5207667<br>3|dbj|BAD4<br>5573.1| unknown<br>protein	31	18.06681	10	4.619048	9<br>1<br>1<br>3<br>7<br>2	1.9676<br>75	0.0008<br>54	4.<br>9<br>0<br>E-<br>0<br>5
OS0<br>1T0<br>914<br>300-	Plant lipid transfer protein/seed storage/trypsin-alpha amylase inhibitor domain containing protein.	8<br>2<br>2	gi|1135347<br>09|dbj|BAF<br>07092.1| Os01g0914	102	48.38094	50	18.79649	2.<br>5<br>7<br>3	1.3639<br>76	5.59E-<br>07	1.<br>4<br>4<br>E-

01			300					935			08
OS01T0299400-01	Sterile alpha motif homology domain containing protein.	527	gi|57899040|dbj|BAD87814.1| unknown protein	85	62.88605	52	30.49098	2.062448	1.044358	0.000525	2.82E-05
OS01T0357	Ferredoxin-nitrite reductase (EC 1.7.7.1).	249	gi|113532539|dbj|BAF04922.1|	487	76.07297	162	20.05622	3.79	1.923334	8.71E-54	1.34

100-01		6	Os01g0357100					2987			E-56
OS03T0670700-02	Similar to Glycine-rich protein.	853	gi|108710320|gb|ABF98115.1| retrotransposon protein, putative, Ty1-copia subclass,	5569	2545.506	2527	915.4504	2.7806005	1.475399	0	0

OS11T0139900-00	Mitochondrial glycoprotein domain containing protein.	744	expressed gi|77548596|gb|ABA91393.1| Uncharacterised protein family containing protein, expressed	147	77.03541	92	38.21145	2.016003	1.011517	2.77E-06	8.12E-08
OS07T0	Polygalacturonase-inhibiting protein, Inhibitor of fungal polygalacturonase,	14	gi|27497120|gb|AAO1	148	39.82349	58	12.3691	3.2	1.686879	1.38E-13	1.4

568700-02	Regulation of floral organ number	49	7320.1|AF466357_1 floral organ regulator 1					195941			0E-155
OS04T0412100-01	Similar to H0717B12.8 protein.	988	gi|38346335|emb|CAD40657.2| OSJNBa0073L04.6	15	5.919436	1	0.312768	8.92599	4.242297	0.000723	4.03E-05
OS0	Similar to histone H1.	7	no hit	101	54.01817	50	21.1944	2.	1.3497	8.27E-	2.

7T0184850-00		29						5487	62	07	20E-08
OS07T0558400-01	Similar to Chlorophyll a/b-binding protein CP29 precursor.	1221	gi|3075488|gb|AAC14566.1| chlorophyll a/b-binding protein	117	37.3608	61	15.43806	2.420045	1.275034	3.36E-07	8.24E-09
OS0	Conserved hypothetical protein.	1	gi|1135640	27	8.211485	6	1.446245	5.	2.5053	0.0002	1.

4T0309500-01		282	01|dbj|BAF14344.1|Os04g0309500					677797	31	33	10E-05
OS05T0438700-01	Similar to Histone H3.	731	unnamed protein product	182	97.07335	96	40.58191	2.392035	1.258239	7.73E-11	1.12E-12

OS10T0205200-01	Similar to Ufm1-conjugating enzyme 1 (Ubiquitin-fold modifier conjugating enzyme 1).	767	gi|20279484|gb|AAM18764.1|AC099325_20 unknown protein	84	42.7002	40	16.11548	2.6496399	1.405796	4.55E-06	1.41E-07
OS02T0770800-01	Similar to Nitrate reductase [NAD(P)H] (EC 1.7.1.2).	3050	gi|46805324|dbj|BAD16843.1| putative nitrate reductase	177	22.62661	94	9.52372	2.375581	1.248423	2.02E-10	3.10E-12

								6			
OS06T0545900-01	Protein of unknown function DUF246, plant family protein.	25550	gi|53791993|dbj|BAD54578.1| putative axi1	71	10.85586	38	4.604919	2.357448	1.237226	0.000242	1.16E-05
OS06T0163300-01	Harpin-induced 1 domain containing protein.	12266	gi|55296085|dbj|BAD67647.1| unknown protein	94	28.94944	58	14.15705	2.0448	1.032014	0.000266	1.29E-0

								77			5
OS05T0149300-00	Similar to 1-aminocyclopropane-1-carboxylate oxidase.	1031	gi|113578207|dbj|BAF16570.1| Os05g0149300	57	21.5557	27	8.092519	2.663658	1.413409	0.000273	1.34E-05
OS03T0692700-	Similar to predicted protein.	1016	hypothetical protein OsI_13104	52	19.95518	19	5.778811	3.453	1.787919	2.25E-05	8.24E-

05								163			07
OS12T0235200-01	Conserved hypothetical protein.	799	gi|297728989|ref|NP_001176858.1|Os12g0235300	53	25.86277	25	9.668783	2.6774873	1.419471	0.000479	2.53E-05
OS06T0156	Hypothetical protein.	108	no hit	39	14.04049	6	1.711991	8.220	3.035846	2.23E-07	5.224

650-00		3						1263			E-09
OS12T0569200-01	Conserved hypothetical protein.	800	gi|77556849|gb|ABA99645.1| expressed protein	380	185.1994	221	85.36521	2.1694995	1.117359	1.25E-18	8.388E-21
OS08T0	Ribosomal protein L30 family protein.	74	gi|42408383|dbj|BAD0	89	46.82931	28	11.67665	4.0	2.003785	2.15E-10	3.3

110100-01		1	9534.1| ribosomal protein L30p family-like					10508			4E-12
OS02T0629800-01	Similar to Defensin precursor.	602	hypothetical protein OsI_08161	57	36.91683	19	9.752944	3.785198	1.920369	2.11E-06	6.04E-08
OS0	Cytochrome P450 family protein.	1	gi|4639052	33	7.686072	8	1.476771	5.	2.3798	5.97E-	2.

2T0204700-00		674	2|dbj|BAD16010.1| putative cytochrome P450 monooxygenase					204648	01	05	39E-006
OS08T0239300-01	Dienelactone hydrolase domain containing protein.	11135	gi|113623198|dbj|BAF23243.1| Os08g0239300	38	13.0537	15	4.08389	3.19639	1.676443	0.000961	5.62E-005

OS05T0439200-01	Hypothetical protein.	8002	gi|113579215|dbj|BAF17578.1| Os05g0439200, partial	66	32.086	29	11.17383	2.871533	1.52182	2.05E-05	7.41E-07
OS06T0199402-00	Similar to predicted protein.	3390	gi|51091831|dbj|BAD36645.1| unknown protein	28	27.99235	1	0.792344	5.32852	5.142761	2.50E-07	5.96E-09

OS08T0481400-01	Similar to HAHB-7 (Fragment).	14009	gi|42409030|dbj|BAD10283.1| putative homeodomain leucine zipper protein	73	20.2003	30	6.579439	3.0702166	1.61834	1.83E-06	5.19E-08
OS12T0632000-01	Gly-rich protein, Partner protein of RF5, Fertility restoration	815	gi|77557139|gb|ABA99935.1| Glycine-rich RNA-bindin	3452	1651.426	1485	563.0506	2.99329	1.552376	########	#####

<table>
<tr><th></th><th></th><th></th><th></th><th></th><th></th><th></th><th></th><th></th><th></th><th></th><th></th></tr>
<tr><td></td><td></td><td></td><td>g protein GRP1A, putative, expressed</td><td></td><td></td><td></td><td></td><td>98</td><td></td><td></td><td>###</td></tr>
<tr><td>OS07T0107300-01</td><td>Plant disease resistance response protein family protein.</td><td>958</td><td>gi|23617048|dbj|BAC20736.1| putative disease resistance response protein-related/dirigent</td><td>81</td><td>32.96594</td><td>40</td><td>12.90248</td><td>2.555009</td><td>1.353328</td><td>1.47E-05</td><td>5.13E-07</td></tr>
</table>

			protein-related								
OS03T0265666-00	Hypothetical conserved gene.	3333	hypothetical protein OsI_10867	14	16.39192	0.5	0.463985	35.32852	5.142761	0.000701	3.89E-05
OS10T0377300-	Similar to Homeobox-leucine zipper protein HOX8.	1296	gi|78708410|gb|ABB47385.1| Homeobox	66	19.85569	36	8.583731	2.3133	1.209876	0.0006	3.27E-

01			domain containing protein, expressed					1 7 7			0 5
OS0 1T0 933 075- 01	Hypothetical protein.	1 2 0 4	auxin transport protein-like protein	38	12.30561	8	2.053251	5. 9 9 3 2 3 1	2.5833 34	3.70E- 06	1. 1 3 E- 0 7
OS0 8T0	Similar to Nitrate reductase.	3 1	gi|4240740 0|dbj|BAD0	184	22.80369	95	9.331329	2. 4	1.2891 13	2.26E- 11	3. 1

468100-01		46	9558.1| nitrate reductase apoenzyme					43777			1E-13
OS07T0107366-01	Hypothetical gene.	1001	no hit	52	20.25421	16	4.93929	4.100632	2.035846	2.60E-06	7.57E-08
OS0	Similar to cDNA clone:J033124M18,	1	hypothetical	100	20.79432	58	9.558843	2.	1.1212	3.91E-	1.

3T0640800-02	full insert sequence.	875	protein OsJ_11861					175401	82	05	49E-06
OS03T0634400-01	Serine/threonine protein kinase domain containing protein.	15559	gi|41469487|gb|AAS07272.1| putative serine/threonine protein kinase	62	15.50571	23	4.558903	3.401193	1.766041	3.16E-06	9.44E-08

OS11T0149100-01	Protein of unknown function DUF296 domain containing protein.	1579	gi|62701672|gb|AAX92745.1| expressed protein	74	18.2724	44	8.610912	2.122005	1.085428	0.000948	5.52E-05
OS06T0543200-01	Similar to CDPK substrate protein 1.	1940	gi|53793087|dbj|BAD54297.1| MYB transcription factor-like	44	8.842945	17	2.707857	3.26566	1.707375	0.000237	1.13E-05

OS03T0113000-01	Serine/threonine protein kinase domain containing protein.	1609	gi|27476081|gb|AAO17012.1| Hypothetical protein	65	15.75082	31	5.953663	12.645569	1.403578	8.92E-05	3.75E-06
OS02T0620600-01	Ammonium transporter family protein.	1853	gi|47847757|dbj|BAD21534.1| putative ammonium	130	27.35356	73	12.1738	2.2469	1.16795	5.58E-07	1.43E-0

			transporter 1-3					21			8
OS03T0279200-01	Similar to Histone H2A.	753	gi|115452261|ref|NP_001049731.1| Os03g0279200	605	313.261	225	92.33496	3.392659	1.762417	2.21E-59	3.06E-62
OS07T0658600-	Similar to Nucleoid DNA-binding-like protein.	1677	gi|22775625|dbj|BAC15479.1| nucleoid	24	5.579871	5	0.921331	6.056	2.598441	0.000446	2.34E-

00			DNA-binding-like protein					317			05
OS07T0631500-01	Similar to Mov34/MPN/PAD-1 family protein.	15442	hypothetical protein OsI_22696	25	6.32123	5	1.001992	6.308664	2.657334	0.000257	1.24E-05
OS04T0401	Hypothetical protein.	139	gi|255675421|dbj|BAH92645.1|	42	11.74715	16	3.546793	3.331	1.727724	0.000305	1.552

751-00		4	Os04g0401750					2049			E-05
OS07T0582700-01	Conserved hypothetical protein.	1773	hypothetical protein OsJ_24895	135	29.68732	45	7.843003	3.785198	1.920369	8.20E-15	7.57E-17
OS02T0	Hypothetical protein.	15	no hit	31	7.762812	2	0.396936	19.	4.289603	1.42E-07	3.2

620566-00		57						55686			3E-09
OS02T0653900-03	Similar to Avr9/Cf-9 rapidly elicited protein 14 (Fragment).	19661	gi|49387523|dbj|BAD24988.1| putative defective in anther dehiscence 1	76	15.11061	42	6.618359	2.283135	1.191016	0.000226	1.07E-05

OS03T0708250-00	Conserved hypothetical protein.	6551	gi|255675462|dbj|BAH92666.1| Os04g0426266, partial	60	35.93488	28	13.29094	2.70037713	1.434942	0.000141	6.24E-06
OS01T0841000-01	ENTH/VHS domain containing protein.	12268	gi|56784669|dbj|BAD81760.1| unknown protein	131	40.28079	77	18.76507	2.146658	1.102043	1.96E-06	5.59E-08

								4			
OS03T0109500-00	Similar to 40S ribosomal protein S17-4.	632	gi|24431604|gb|AAN61484.1| Putative 40S ribosomal protein S17	70	43.18441	40	19.55787	2.208032	1.142761	0.00077	4.35E-05
OS10T0454200-01	Eggshell protein family protein.	805	gi|31432441|gb|AAP54071.1| expressed protein	1151	557.475	692	265.6372	2.09886	1.06945	7.39E-53	1.21E-55

								34			5
OS02T0669000-01	Lipase, GDSL domain containing protein.	14552	gi|50251329|dbj|BAD28305.1| putative anter-specific proline-rich protein APG	96	25.77808	52	11.06663	2.329353	1.219929	1.33E-05	4.58E-07
OS06T0	Conserved hypothetical protein.	97	hypothetical protein	146	58.32423	74	23.42936	2.4	1.315778	2.38E-09	4.1

199400-00		6	OsJ_20466					89365			9E-11
OS12T0117500-01	Hypothetical protein.	1800	no hit	34	7.364655	9	1.545072	4.766546	2.252944	8.56E-05	3.59E-06
OS0	OSH15 protein (Homeobox gene).	1	gi|3327240|	64	17.03289	35	7.382595	2.	1.2061	0.0007	4.

7T0129700-01		465	dbj|BAA31688.1| OSH15					307168	23	92	49E-05
OS08T0502700-01	Pyridoxal phosphate-dependent transferase, major region, subdomain 1 domain containing protein.	1506	gi|42407771|dbj|BAD08917.1| putative aminotransferase	124	32.10278	67	13.74765	2.335147	1.223514	3.69E-07	9.21E-09

<table>
<tr><td>OS04T0290400-01</td><td>Conserved hypothetical protein.</td><td>12772</td><td>gi|255675289|dbj|BAH92570.1| Os04g0290350, partial</td><td>72</td><td>22.06944</td><td>33</td><td>8.016881</td><td>2.752872</td><td>1.460937</td><td>1.51E-05</td><td>5.27E-07</td></tr>
<tr><td>OS02T0793700-01</td><td>Similar to Membrane steroid-binding protein 1.</td><td>3880</td><td>gi|47497145|dbj|BAD19194.1| cytochrome b5 domain-con</td><td>122</td><td>125.1763</td><td>63</td><td>51.23132</td><td>2.443335</td><td>1.288864</td><td>1.30E-07</td><td>2.94E-09</td></tr>
</table>

			taining protein-like					6			
OS03T0126600-01	Conserved hypothetical protein.	1431	gi|108705964|gb|ABF93759.1| expressed protein	66	17.98251	37	7.989888	2.250658	1.170347	0.0009	5.21E-05
OS11T0515000-	Armadillo-like helical domain containing protein.	1428	gi|77551093|gb|ABA93890.1| Armadillo/b	36	9.829248	13	2.813156	3.4494	1.804892	0.000664	3.65E-

00			eta-catenin-like repeat family protein, expressed					029			05
OS03T0690500-00	Similar to Oxidoreductase, 2OG-Fe oxygenase family protein, expressed.	1303	gi|50838953|gb|AAT81714.1| putative oxygenase	41	12.26833	16	3.794497	3.233319	1.692958	0.000476	2.51E-05
OS10T0	Hypothetical conserved gene.	16	unnamed protein	177	41.49798	93	17.28102	2.4	1.263853	1.28E-10	1.9

213800-01		63	product					01362			2E-12
OS03T0223000-02	Similar to Atypical receptor-like kinase MARK.	24450	gi|108706918|gb|ABF94713.1| Leucine Rich Repeat family protein,	89	14.16348	52	6.558671	2.15959504	1.1107	0.000148	6.663E-006

			expressed							
OS01T0935800-01	Similar to BRASSINOSTEROID INSENSITIVE 1-associated receptor kinase 1.	914	hypothetical protein OsJ_04683	17	7.251848	1	0.33809	21.449446	4.422869	0.000214	1.000E-005
OS01T0284500-01	Similar to Nectarin 1 precursor (EC 1.15.1.1) (Superoxide dismutase [Mn]).	925	gi|2655291|gb|AAC04835.1| germin-like protein 4	850	358.2805	342	114.2518	3.13358	1.648873	4.45E-76	4.24E-7

								861			9
OS04T0627900-01	Translation initiation factor SUI1 domain containing protein.	920	OSJNBa0089N06.3	19	8.052148	2	0.67177	1.986466	3.583334	0.000252	1.21E-05
OS10T0552600-	Similar to Tfm5 protein.	757	gi|10140658|gb|AAG13494.1|AC026758_31	183	94.25431	74	30.20748	3.1120	1.641653	3.73E-16	3.06E-

01			putative lipid transfer protein					231			18
OS04T0457000-01	Similar to Chlorophyll a/b-binding protein CP24, photosystem II (Fragment).	1141	gi|21740892|emb|CAD40888.1| OSJNBa0036B21.6	98	33.48778	47	12.7289	2.6308847	1.395527	6.06E-07	1.57E-08
OS02T0	Conserved hypothetical protein.	23	gi|46390154|dbj|BAD1	171	28.09599	104	13.54298	2.0	1.052819	1.00E-07	2.2

564200-01		73	5588.1| unknown protein					7458			4E-09
OS02T0606200-01	Zinc finger, B-box domain containing protein.	1078	gi|3618314|dbj|BAA33203.1| zinc finger protein	375	135.6309	177	50.73797	2.6773163	1.418548	1.03E-26	4.17E-29
OS01T0	CCT domain containing protein.	13	gi|56201945|dbj|BAD7	158	45.19675	62	14.05641	3.2	1.684991	1.74E-14	1.6

835700-01		63	3395.1| zinc finger CONSTANS-like protein					15384			6E-16
OS06T0159450-00	Similar to Histone H3.	867	hypothetical protein OsJ_20207	190	85.44379	98	34.92895	2.4446217	1.290552	9.39E-12	1.23E-13
OS0	Prephenate dehydratase domain	1	gi|1135642	103	24.16307	50	9.29646	2.	1.3780	3.76E-	9.

4T0406600-01	containing protein.	662	87|dbj|BAF14630.1|Os04g0406600					59917	51	07	41E-09
OS09T0528100-01	Similar to 30S ribosomal protein S31, chloroplast (Fragment).	576	gi|52077330|dbj|BAD46371.1|unknown protein	80	54.15187	45	24.14174	2.24308	1.165481	0.000195	9.01E-006
OS01T0	Hypothetical protein.	65	no hit	98	58.15763	47	22.10605	2.6	1.395527	6.06E-07	1.5

149600-00		7						30847			7E-08
OS01T0291733-00	Component of the CCR4-NOTcomplex, Deadenylase, Component of the plant P-body, Deadenylation (poly(A) tail shortening), Development and stress response	1168	gi|108709119|gb|ABF96914.1| hypothetical protein LOC_Os03g33200	75	25.03597	42	11.11182	2.2530394	1.171908	0.000315	1.58E-05
OS0	Uncharacterised conserved protein	1	gi|1255921	83	26.5473	42	10.64692	2.	1.3181	1.78E-	6.

4T0684900-02	UCP037471 domain containing protein.	219	23|gb|EAZ32473.1| hypothetical protein OsJ_16690					493424	28	05	33E-07
OS03T0194900-01	Lipoxygenase, LH2 domain containing protein.	15330	gi|24414270|gb|AAN59773.1| Putative membrane protein	161	41.02801	94	18.98519	2.1611053	1.111734	6.15E-08	1.33E-09

OS02T0753300-01	Conserved hypothetical protein.	897	gi|46390131|dbj|BAD15566.1| putative elicitor-inducible protein EIG-J7	215	93.45273	85	29.28229	3.1914412	1.674208	1.66E-19	1.04E-21
OS03T0360300-01	Similar to Peroxidase 56 precursor (EC 1.11.1.7) (Atperox P56) (ATP33).	836	gi|108708279|gb|ABF96074.1| expressed protein	88	41.04142	48	17.74245	2.31331	1.209876	4.20E-05	1.61E-0

								77			6
OS03T0563600-01	Tetraspanin domain containing protein.	14044	gi|55700965|tpe|CAH69291.1| TPA: class III peroxidase 49 precursor	121	33.60193	75	16.50718	2.035596	1.025451	2.33E-05	8.56E-07
OS08T0260	Similar to Ethylene responsive element binding factor3 (OsERF3).	1009	gi|37806167|dbj|BAC99671.1|	30	10.73101	9	2.551494	4.220	2.072372	0.000657	3.661

600-01		0	putative senescence-associated protein					5776			E-05
OS03T0182800-01	Allergen V5/Tpx-1 related family protein.	13321	gi|33087061|gb|AAP92744.1| ap2 domain containing protein	182	53.71735	99	23.15853	2.3319549	1.213844	2.93E-10	4.64E-112
OS07T0	Similar to Homeobox-leucine zipper protein HOX25.	67	gi|28201315|dbj|BAC5	240	138.2193	130	59.33805	2.3	1.219929	1.48E-13	1.5

126100-00		7	6823.1| putative pathogenesis-related protein					29353			4E-15 5
OS09T0379600-00	Conserved hypothetical protein.	1275	RecName: Full=Homeobox-leucine zipper protein HOX25; AltName: Full=HD-ZI	206	62.99456	109	26.41769	2.384559	1.253723	3.62E-12	4.43E-14

			P protein HOX25; AltName: Full=Homeodomain transcription factor HOX25; AltName: Full=OsHox25								
OS07T0	Conserved hypothetical protein.	84	gi|255677943|dbj|BAH	71	32.64438	29	10.56771	3.0	1.627173	2.42E-06	7.0

597400-01		8	94013.1| Os07g0597400, partial					8907			3E-08
OS01T0346250-00	Hypothetical protein.	1264	gi|255673202|dbj|BAH91053.1| Os01g0346501	45	13.8807	20	4.889467	2.8838899	1.505331	0.00087	5.02E-05
OS01T0	Hypothetical protein.	58	no hit	31	20.52071	9	4.721781	4.3	2.119678	0.000394	2.0

865900-00		9						459681			4E-05
OS03T0807150-00	Conserved hypothetical protein.	13005	no hit	41	12.24953	5	1.183963	0.346621	3.37103	1.79E-08	3.54E-10
OS0	Protein of unknown function	8	no hit	164	76.6697	76	28.15958	2.	1.4450	4.77E-	5.

3T0<br>575<br>500-<br>02	DUF1070 family protein.	3<br>4						7<br>2<br>2<br>6<br>8<br>7	31	12	9<br>6<br>E-<br>1<br>4
OS0<br>2T0<br>264<br>800-<br>01	Similar to Ammonium transporter 1 member 1.	5<br>1<br>9	gi|5025195<br>0|dbj|BAD2<br>7885.1| unknown<br>protein	82	61.60167	33	19.64831	3.<br>1<br>3<br>5<br>2<br>1<br>5	1.6485<br>64	2.13E-<br>07	4.<br>9<br>9<br>E-<br>0<br>9

OS04T0509600-01	Hypothetical gene.	20443	gi|32488298|emb|CAE03364.1| OSJNBb0065L13.7	59	11.25977	25	3.781379	2.977689	1.574193	4.18E-05	1.60E-06
OS04T0345100-01	Arm repeat protein.	5882	no hit	39	26.12688	9	4.778572	5.46750	2.450884	5.74E-06	1.82E-07

								9			
OS01T0823900-01	Similar to predicted protein.	2410	gi|21104594|dbj|BAB93187.1| putative arm repeat protein	58	9.383329	24	3.077321	3.0499188	1.608425	3.57E-05	1.35E-06
OS01T0919600-02	Conserved hypothetical protein.	10161	gi|57899847|dbj|BAD87631.1| lysine ketoglutarat	68	24.98846	29	8.446197	2.9585	1.564888	8.86E-06	2.92E-0

			e reductase trans-splicing related 1-like					46			7
OS06T0508800-01	Zinc finger, C2H2-like domain containing protein.	582	gi|222635659|gb|EEE65791.1| hypothetical protein OsJ_21496	34	22.77728	8	4.247619	5.362364	2.422869	3.45E-05	1.31E-06
OS06T0	Similar to predicted protein.	77	gi|53792608|dbj|BAD5	1050	530.2956	435	174.1208	3.0	1.606708	1.18E-90	8.7

708600-01		2	3623.1| zinc finger protein-like					45562			3E-94
OS02T0528100-02	Conserved hypothetical protein.	19551	unnamed protein product	26	5.195915	5	0.791938	6.56101	2.713918	0.000148	6.58E-06
OS01T0	Hypothetical conserved gene.	81	gi|55295964|dbj|BAD6	167	79.79438	80	30.29552	2.6	1.397183	1.13E-11	1.5

644 900- 01		6	8004.1| unknown protein					3 3 8 6 7			0 E- 1 3
OS1 0T0 418 300- 01	Hypothetical protein.	9 0 0	hypothetical protein OsI_33595	76	32.92434	39	13.39062	2. 4 5 8 8 7 6 1	1.2979 32	6.18E- 05	2. 4 8 E- 0 6
OS0	Kelch-type beta propeller domain	7	gi|2240317	22	11.60711	3	1.254456	9.	3.2098	0.0001	6.

1T0 270 501- 00	containing protein.	3 9	25|gb|ACN 34938.1| unknown					2 5 2 7 0 7	76	52	8 2 E- 0 6
OS1 1T0 246 200- 00	Protein of unknown function DUF640 domain containing protein.	1 2 3 2	gi|6270184 9|gb|AAX92 922.1| Kelch motif, putative	700	221.5304	424	106.3491	2. 0 8 3 0 4 9	1.0586 97	1.64E- 31	5. 4 9 E- 3 4

OS06T0672400-01	Hypothetical protein.	12224	gi|52077000|dbj|BAD46009.1| unknown protein	110	35.03945	64	16.15761	2.168660 3	1.116766	1.41E-05	4.888E-07
OS08T0113150-00	Similar to nodulin-like protein 5NG4.	8006	no hit	141	68.20717	73	27.98765	2.437704	1.285133	1.02E-08	1.94E-10

								5			
OS08T0561500-01	Conserved hypothetical protein.	1600	putative MtN21	143	34.84673	77	14.87131	2.343218	1.228491	2.94E-08	6.06E-10
OS03T0785100-01	Similar to atypical receptor-like kinase MARK.	7800	no hit	242	120.967	65	25.75119	4.6975	2.231902	3.07E-32	9.94E-3

								28			5
OS03T0223000-03	Protein of unknown function DUF247, plant family protein.	1123	gi|108706918|gb|ABF94713.1| Leucine Rich Repeat family protein, expressed	63	21.87292	25	6.879215	3.1795677	1.66883	7.22E-06	2.34E-07
OS09T0	ATP synthase delta' chain, mitochondrial precursor (EC	19	gi|113631050|dbj|BAF	63	12.28779	32	4.946702	2.4	1.312686	0.000305	1.55

<table>
<tr><td>300 800- 01</td><td>3.6.3.14).</td><td>9 9</td><td>24731.1| Os09g0300 800</td><td></td><td></td><td></td><td></td><td>8 4 0 3 6</td><td></td><td></td><td>2 E- 0 5</td></tr>
<tr><td>OS0 7T0 495 400- 00</td><td>Transferase family protein.</td><td>3 2 7</td><td>gi|5050919 9|dbj|BAD3 0403.1| unknown protein</td><td>28</td><td>33.38538</td><td>7</td><td>6.614985</td><td>5. 0 4 6 9 3 1</td><td>2.3354 06</td><td>0.0003 42</td><td>1. 7 3 E- 0 5</td></tr>
<tr><td>OS0</td><td>Hypothetical conserved gene.</td><td>1</td><td>gi|1103458</td><td>72</td><td>17.36075</td><td>35</td><td>6.688622</td><td>2.</td><td>1.3760</td><td>4.23E-</td><td>1.</td></tr>
</table>

1T0<br>615<br>200-<br>01		6<br>1<br>7	5|dbj|BAB1<br>7109.1| 10-deacetyl<br>baccatin<br>III-10-O-ace<br>tyl<br>transferase-<br>like					5<br>9<br>5<br>5<br>6<br>5	48	05	6<br>3<br>E-<br>0<br>6
OS0<br>1T0<br>104<br>900-<br>02	Hypothetical protein.	1<br>1<br>4<br>6	gi|1069720<br>7|dbj|BAB1<br>6338.1| hsr201 -like	69	23.47526	27	7.280442	3.<br>2<br>2<br>4<br>4	1.6890<br>43	1.71E-<br>06	4.<br>8<br>4<br>E-<br>0

								28			8
OS07T0448300-00	Similar to Peroxidase (EC 1.11.1.7).	776	gi|238014212|gb|ACR38141.1| unknown	121	60.79525	74	29.46786	2.063104	1.044816	1.64E-05	5.788E-07
OS05T0499300-	Similar to Peroxidase 1.	1347	gi|51038054|gb|AAT93858.1| peroxidase	208	60.20627	111	25.46443	2.3364	1.24143	4.24E-12	5.24E-

01								328			14
OS10T0536700-01	Similar to Histone H4.	14221	gi|18057099|gb|AAL58122.1|AC092697_10 putative peroxidase	79	21.67599	45	9.785816	2.2150442	1.147334	0.00027	1.322E-05
OS02T0684	Heavy metal transport/detoxification protein domain containing protein.	515	TPA: histone H4.3	219	165.7994	103	61.80286	2.68	1.423693	1.12E-15	9.443

500-00								2713			E-18
OS03T0152000-01	Curculin-like (mannose-binding) lectin domain containing protein.	1400	gi|108706224|gb|ABF94019.1| heavy metal-associated domain containing protein,	127	35.36891	80	17.65796	2.003001	1.002163	2.07E-05	7.49E-07

			expressed								
OS10T0155100-01	Similar to Pectin-glucuronyltransferase.	1436	gi|18542891|gb|AAL75733.1|AC091724_6 Unknown protein	222	60.27601	79	17.00009	3.545629	1.826042	9.93E-23	4.92E-25
OS01T0926400-03	Pollen Ole e 1 allergen and extensin domain containing protein.	16621	gi|20160726|dbj|BAB89668.1| P0482D04.15	28	6.734743	5	0.95316	7.0657	2.820833	4.67E-05	1.83E-0

								04			6
OS05T0531200-01	Amino acid transporter, transmembrane domain containing protein.	1050	gi|45642728|gb|AAS72356.1| unknown protein	56	20.79432	19	5.591688	3.718791	1.894834	3.40E-06	1.03E-07
OS01T0856500-	Similar to Light-harvesting complex I (Fragment).	1967	gi|56785353|dbj|BAD82311.1| putative	46	9.117997	19	2.984887	3.054	1.611041	0.000328	1.65E-

<table>
<tr><td>02</td><td></td><td></td><td>AUX1-like permease</td><td></td><td></td><td></td><td></td><td>721</td><td></td><td></td><td>05</td></tr>
<tr><td>OS06T0320500-01</td><td>Similar to Mitochondrial carnitine/acylcarnitine carrier-like protein (A BOUT DE SOUFFLE) (Carnitine/acylcarnitine translocase-like protein) (CAC-like protein).</td><td>1048</td><td>gi|3789954|gb|AAC67558.1| chlorophyll a/b-binding protein precursor</td><td>119</td><td>44.27226</td><td>72</td><td>21.22999</td><td>2.0855364</td><td>1.060299</td><td>1.50E-05</td><td>5.2550E-07</td></tr>
<tr><td>OS10T0573</td><td>Similar to Thaumatin-like protein.</td><td>1220</td><td>gi|12643054|gb|AAK00443.1|AC06</td><td>44</td><td>14.29609</td><td>15</td><td>3.862679</td><td>3.770</td><td>1.887947</td><td>6.28E-05</td><td>2.553</td></tr>
</table>

700- 02		0	0755_13 putative carnitine/ac ylcarnitine translocase					1 0 8 3			E- 0 6
OS0 3T0 244 200- 02	Similar to leucine-rich repeat protein kinase.	1 5 8 0	gi|1087071 32|gb|ABF9 4927.1| SCUTL1, putative, expressed	88	21.71559	30	5.86736	3. 7 0 1 0 8 3	1.8879 47	1.64E- 09	2. 8 3 E- 1 1
OS0	Hypothetical protein.	1	ERECTA-lik	51	10.7079	24	3.993723	2.	1.4228	0.0006	3.

9T0<br>436<br>500-<br>03		8<br>5<br>7	e kinase					6<br>8<br>1<br>1<br>8<br>2	69	39	4<br>9<br>E-<br>0<br>5
OS0<br>6T0<br>696<br>450-<br>00	Protein of unknown function DUF250 domain containing protein.	8<br>9<br>7	hypothetical protein OsI_24314	28	12.17059	8	2.755981	4.<br>4<br>1<br>6<br>0<br>6<br>5	2.1427<br>61	0.0008<br>04	4.<br>5<br>7<br>E-<br>0<br>5

OS05T0494500-02	Conserved hypothetical protein.	1311	gi|52353475|gb|AAU44041.1| unknown protein	144	42.82583	90	21.2138	2.018772	1.013478	3.47E-06	1.05E-07
OS06T0131300-00	Hypothetical protein.	2109	gi|6006369|dbj|BAA84799.1| arginine decarboxylase	432	79.86439	244	35.7513	2.23388	1.159557	1.80E-22	9.07E-25

								8			
OS11T0474100-01	Embryo-specific 3 family protein.	776	hypothetical protein OsJ_33864	83	41.70253	36	14.33572	2.908995	1.540521	8.05E-07	2.13E-08
OS08T0468200-01	WRKY transcription factor 68.	1811	no hit	66	14.20926	32	5.46022	2.60223	1.379801	9.91E-05	4.222E-0

								24			6
OS01T0172800-01	Hypothetical protein.	822	gi|15128219|dbj|BAB62547.1| unknown protein	35	16.6013	9	3.383368	4.906739	2.294764	5.05E-05	1.99E-06
OS04T0605100-	Pathogenesis-related transcriptional factor/ERF, DNA-binding domain containing protein.	1141	gi|38346908|emb|CAE03880.2| OSJNBb00	687	234.7562	423	114.5601	2.049	1.035059	8.58E-30	3.09E-

01			15N08.8					197			32
OS07T0176400-00	Conserved hypothetical protein.	969	TPA: hypothetical protein ZEAMMB73_001640	253	101.7988	117	37.31133	2.728362	1.448035	1.42E-18	9.54E-21
OS07T0227	Ubiquitin.	849	gi|50510280|dbj|BAD31677.1|	68	31.22822	26	9.463336	3.229	1.72243	1.42E-06	3.96

600-00			pathogenesis-related genes transcriptional activator Pti6-like protein					9916			E-08
OS02T0145600-01	Photosystem I protein-like protein.	1772	gi|113535406|dbj|BAF07789.1| Os02g0145600, partial	60	13.20181	28	4.882845	2.703737	1.434942	0.000141	6.24E-0

								13			6
OS09T0452700-00	HAD-superfamily hydrolase subfamily IIB protein.	790	gi|51535937|dbj|BAD38019.1| putative ubiquitin / ribosomal protein CEP52	87	42.93764	48	18.77555	2.2286891	1.193387	5.97E-05	2.38E-06
OS07T0148	Similar to Histone H4.	599	gi|29367389|gb|AAO72567.1|	65	42.30898	25	12.89709	3.228	1.713918	2.95E-06	8.772

900-<br>01			photosyste<br>m I<br>protein-like<br>protein					0<br>5<br>0<br>5			E-<br>0<br>8
OS0<br>3T0<br>224<br>300-<br>01	Hypothetical protein.	2<br>6<br>2<br>3	gi|1087069<br>33|gb|ABF9<br>4728.1| Trehalose-6<br>-phosphate<br>synthase,<br>putative,<br>expressed	70	10.40509	32	3.769904	2.<br>7<br>6<br>0<br>0<br>4	1.4646<br>89	2.00E-<br>05	7.<br>2<br>2<br>E-<br>0<br>7
OS1	Similar to CI2C.	5	TPA:	339	242.0767	165	93.38345	2.	1.3742	5.48E-	2.

0T0539500-01		46	histone H4.3					592287	26	23	65E-25
OS08T0557850-00	Serine/threonine protein kinase-related domain containing protein.	13116	no hit	120	35.5526	52	12.21029	2.9111691	1.541857	9.46E-10	1.59E-11

OS12T0548501-01	Protein of unknown function DUF3615 domain containing protein.	5060	gi|222617243|gb|EEE53375.1| hypothetical protein OsJ_36418	55	42.37973	28	17.09961	2.4788404	1.309411	0.000942	5.48E-05
OS01T0170300-01	Conserved hypothetical protein.	4008	gi|125569191|gb|EAZ10706.1| hypothetical protein OsJ_00540	108	10.50611	51	3.932068	2.6719	1.417869	8.59E-08	1.89E-09

OS10T0550200-01	Similar to Prep (Fragment).	1179	gi|113639848|dbj|BAF27153.1| Os10g0550200, partial	102	33.73124	63	16.51222	52.042805	1.030552	0.00013	5.69E-006
OS11T0141800-01	Hypothetical gene.	6772	no hit	167	96.89317	72	33.10868	2.9265	1.549186	1.44E-13	1.49E-1

											5
OS10T0534900-01	Curculin-like (mannose-binding) lectin domain containing protein.	23397	gi|22002143|gb|AAM88627.1| putative homeodomain protein	42	6.831676	13	1.675922	194.0763676	2.027284	3.78E-05	1.44E-06
OS05T0114800-	Protein kinase, core domain containing protein.	4722	no hit	29	23.95532	7	4.582839	5.227	2.386032	0.000203	9.46E-

01								179			06
OS09T0535400-00	Similar to Receptor protein kinase.	1372	gi|50726582|dbj|BAD34216.1| hypothetical protein	49	13.92477	22	4.95504	2.8102223	1.490685	0.0005	2.66E-05
OS04T0576	Ubiquitin-conjugating enzyme/RWD-like domain containing protein.	2337	gi|113565199|dbj|BAF15542.1|	70	11.49644	33	4.295481	2.67	1.420295	3.40E-05	1.28

900-01		4	Os04g0576900, partial					6403			E-06
OS07T0542400-01	Similar to Beta-expansin.	19974	gi|34395233|dbj|BAC83762.1| putative serine/threonine kinase -related protein	97	19.1589	38	5.948604	3.220739	1.687392	5.45E-09	1.01E-100
OS0	Conserved hypothetical protein.	1	gi|5025141	253	63.19222	148	29.29796	2.	1.1089	2.72E-	3.

2T0833300-01		561	5|dbj|BAD28453.1| putative human tumor susceptibility gene-like protein					156881	47	12	29E-14
OS10T0555900-01	Similar to ubiquitin-protein ligase.	10031	gi|8118423|gb|AAF72984.1|AF261271_2 beta-expan	72	27.22826	42	12.58836	2.16229	1.113014	0.000858	4.993E-0

			sin					7			5
OS10T0330000-01	Similar to Pentatricopeptide repeat protein PPR986-12.	749	gi|16905202|gb|AAL31072.1|AC091749_1 unknown protein	142	73.91839	58	23.92901	3.08907	1.627173	2.09E-12	2.47E-14
OS02T0548700-00	Bet v I allergen family protein.	1399	gi|46390762|dbj|BAD16270.1| putative immediate-early fungal	150	41.80416	87	19.21676	2.17540	1.121282	1.64E-07	3.77E-09

			elicitor protein CMPG1					1			
OS02T0581900-01	Hypothetical conserved gene.	941	gi|255671029|dbj|BAH91768.1| Os02g0582100, partial	75	31.07546	15	4.925839	6.3086664	2.657334	3.10E-12	3.77E-14
OS06T0562	Hypothetical protein.	880	gi|53791798|dbj|BAD53743.1| Bet	76	33.67262	45	15.80187	2.13	1.091481	0.000725	4.05

200-01			v l allergen-like					0926			E-05
OS10T0556200-01	Rapid ALkalinization Factor family protein.	1914	hypothetical protein OsI_34609	51	10.38901	22	3.551889	2.924926	1.5484	0.000222	1.05E-05
OS03T0	Similar to DnaJ protein.	14	gi|149360813|gb|EDM	128	34.13568	77	16.27504	2.0	1.06862	5.22E-06	1.6

305150-00		62	49264.1| hypothetical protein MDG893_07700					97426			4E-07
OS01T0257100-01	Similar to NADPH HC toxin reductase (Fragment).	691	gi|6016877|dbj|BAA85220.1| putative RALF precursor	70	39.49717	31	13.86316	2.849074	1.510493	1.18E-05	3.98E-07
OS0	Similar to HvB12D protein (B12Dg1	8	gi|2936735	447	195.1651	245	84.77996	2.	1.2028	1.16E-	5.

3T0648400-01	protein).	93	7|gb|AAO72551.1| DNAJ-like protein					302019	99	24	20E-27
OS07T0601000-01	Hypothetical protein.	1161	gi|33146534|dbj|BAC79711.1| putative NADPH HC toxin reductase	36	12.08972	8	2.129298	5.677797	2.505331	1.15E-05	3.86E-07

OS06T0246000-01	Similar to GTP-binding protein.	573	gi|2293568|gb|AAB65433.1| HvB12D homolog	6423	4370.482	3532	1904.779	2.294482	1.198168	0	0
OS02T0658150-00	FAS1 domain domain containing protein.	3334	gi|219886967|gb|ACL53858.1| unknown	49	57.19994	18	16.65347	3.43471	1.780191	4.68E-05	1.83E-06

								7			
OS01T0667600-01	Hypothetical protein.	977	gi|20161585|dbj|BAB90506.1| putative GTP-binding protein Rab11b	53	21.15082	24	7.590935	2.786327	1.478364	0.000286	1.41E-05
OS05T0563550-00	Plastocyanin, chloroplast precursor.	756	gi|50511473|gb|AAT77395.1| hypothetical protein	92	47.44736	23	9.401229	5.04469	2.335406	6.03E-13	6.72E-1

								31			5
OS01T0914200-01	Serine/threonine protein kinase-related domain containing protein.	2251	no hit	39	6.755152	12	1.647344	4.100632	2.035846	7.75E-05	3.21E-06
OS06T0101600-	Protein of unknown function DUF247, plant domain containing protein.	7002	gi|3885896|gb|AAC78108.1|plastocyani	74	41.09988	41	18.04784	2.2277	1.187308	0.000296	1.47E-

01			n precursor					274			05
OS01T0607900-01	Similar to Dirigent-like protein (Fragment).	2525	gi|53793399|dbj|BAD53058.1| receptor-like protein kinase 1-like	76	11.73541	30	3.671457	3.19639	1.676443	4.81E-07	1.22E-08
OS08T0337	Hypothetical protein.	1377	hypothetical protein	41	11.65983	13	2.930114	3.97	1.992519	6.22E-05	2.50

800-00		1						9311			E-06
OS07T0638500-01	Hypothetical protein.	10400	gi|27261086|dbj|BAC45199.1| putative disease resistance response protein	49	18.36998	18	5.348325	3.434717 17	1.780191	4.68E-05	1.833E-06
OS0	Thaumatin, pathogenesis-related	1	gi|1136389	30	7.398358	7	1.368185	5.	2.4349	0.0001	5.

2T0203200-01	family protein.	581	77|dbj|BAF26282.1|Os10g0331500					407426	42	18	15E-06
OS04T0402700-01	NPH3 domain containing protein.	564	gi|113564266|dbj|BAF14609.1|Os04g0402700	51	35.25633	20	10.95795	3.217419	1.685904	6.86E-05	2.79E-06

OS12T0569300-01	Similar to Photosystem I reaction centre subunit N, chloroplast precursor (PSI- N).	1003	gi|77556850|gb|ABA99646.1| thaumatin-like cytokinin-binding protein, putative, expressed	126	48.97964	51	15.71259	3.1172222	1.640261	3.65E-11	5.111E-113
OS03T0738	Similar to Cell wall beta-glucosidase.	197	gi|255674881|dbj|BAF13129.2|	26	5.143192	5	0.783902	6.556	2.713918	0.000148	6.558

900-00	1	Os03g0738900					1 0 1			E-0 6
OS12T0189400-01	665 6	gi|77553228|gb|ABA96024.1| Photosystem I reaction centre subunit N, chloroplast precursor, putative,	81	48.14234	37	17.42916	2.7621 72	1.465803	3.21E-06	9.63E-08

OS03T0749300-04		5524	expressed gi|18087674|gb|AAL58966.1|AC091811_15 putative exoglucanase precursor	63	46.87651	13	7.666386	6.1145551	2.612247	3.91E-10	6.31E-1122
OS02T0670900-	Similar to amino acid transport protein.	1697	gi|50251349|dbj|BAD28325.1| putative	101	23.20521	63	11.47195	2.0222	1.016338	0.000176	8.04E-

05			amino acid transport protein					778			06
OS07T0588900-01	Glycosyl transferase, family 43 protein.	918	gi|28564819|dbj|BAC57748.1| unknown protein	38	16.13938	15	5.049254	3.196639	1.676443	0.000961	5.62E-05
OS10T0418000-	Similar to Histone H2A.	799	gi|20042981|gb|AAM08789.1|AC016780_19	125	60.9971	63	24.36533	2.5503	1.323911	4.06E-08	8.60E-

01			Putative histone H2A					438			10
OS06T0544100-01	Tyrosine protein kinase domain containing protein.	2298	unnamed protein product	48	8.143989	15	2.017065	4.037545	2.013478	8.83E-06	2.91E-07
OS03T0183	Similar to AP2 domain containing protein, expressed.	749	gi|113547650|dbj|BAF11093.1|	219	114.0009	118	48.68316	2.34	1.22755	1.66E-12	1.94

200-01			Os03g0183200					169			E-14
OS02T0720450-00	Hypothetical protein.	1103	unknown	32	11.31151	7	1.961106	5.767921	2.528051	3.95E-05	1.51E-06
OS08T0129	Conserved hypothetical protein.	4998	gi|29467529|dbj|BAC66718.1|	23	18.00713	5	3.102553	5.80	2.53704	0.000776	4.39

900-00			hypothetical protein					3971			E-05
OS09T0397700-01	Glutathione-dependent formaldehyde-activating, GFA family protein.	782	gi|50252614|dbj|BAD28785.1| putative nuclear protein p30	44	21.93774	19	7.508021	2.921907	1.54691	0.000763	4.30E-05
OS10T0	TGF-beta receptor, type I/II extracellular region family protein.	19	gi|255679617|dbj|BAF	84	16.38372	49	7.574638	2.1	1.113014	0.000243	1.11

554 200- 01		9 9	27178.2| Os10g0554 200, partial					6 2 9 7			6 E- 0 5
OS0 5T0 427 400- 00	Similar to Phenylalanine ammonia-lyase.	2 1 5 1	gi|4639113 3|gb|AAS90 660.1| putative phenylalani ne ammonia-ly ase (EC 4.3.1.5)	93	16.85732	56	8.045003	2. 0 9 5 3 7 8	1.0672 1	0.0001 8	8. 2 5 E- 0 6

OS10T0376200-01	Leucine-rich repeat, typical subtype containing protein.	2521	gi|19920101|gb|AAM08533.1|AC079935_5 Putative protein with similarityto protein phosphatase PP1 regulatory subunit	106	16.39378	55	6.741685	2.4317033	1.281967	1.31E-06	3.61E-08
OS0	Conserved hypothetical protein.	4	gi|4938895	29	23.60524	4	2.580495	9.	3.1933	7.81E-	2.

2T0230000-01		79	0|dbj|BAD26170.1| unknown protein					147563	87	06	55E-07
OS04T0567800-01	Similar to OSIGBa0103M18.3 protein.	1556	hypothetical protein OsI_17020	120	30.06891	62	12.31291	2.4442063	1.288101	1.74E-07	4.02E-09

OS01T0223600-01	Similar to Pto kinase interactor 1-like protein.	17730	gi|113531977|dbj|BAF04360.1| Os01g0223600, partial	98	22.08645	59	10.53864	2.0957 6	1.067473	0.000109	4.70E-06
OS01T0726100-01	Pollen Ole e 1 allergen and extensin domain containing protein.	10005	gi|20161141|dbj|BAB90069.1| unknown protein	28	10.8627	7	2.152339	5.0469 31	2.335406	0.000342	1.73E-05

OS03T0687100-00	Hypothetical protein.	14994	unknown	46	12.00475	19	3.929901	3.05472 1	1.611041	0.000328	1.655E-005
OS03T0385400-01	Bifunctional inhibitor/plant lipid transfer protein/seed storage domain containing protein.	864	gi|50881461|gb|AAT85306.1| protease inhibitor/seed	81	36.55251	34	12.16029	3.00589	1.587794	5.97E-07	1.54E-008

			storage/LTP family protein					3			
OS03T0243900-01	Similar to Thaumatin-like protein.	1720	gi|108707129|gb|ABF94924.1| Thaumatin family protein, expressed	83	18.81463	52	9.342293	2.01392	1.010006	0.000968	5.67E-05
OS04T0565	Similar to OSIGBa0158F05.8 protein.	1550	gi|215678668|dbj|BAG92323.1|	62	16.1156	15	3.090143	5.21	2.382712	5.28E-09	9.74

500-01		0	unnamed protein product					5162			E-11
OS02T0729300-01	Zinc finger, CCHC retroviral-type domain containing protein.	37669	gi|113537536|dbj|BAF09919.1| Os02g0729300	50	5.172373	21	1.721757	3.0041266	1.586945	0.000193	8.89E-06
OS07T0	Similar to transcriptional regulatory protein algP.	63	hypothetical protein	477	291.0473	251	121.3812	2.3	1.261708	2.11E-28	8.0

590 700- 01		9	OsJ_24953					9 7 7 9 5			4 E- 3 1
OS0 4T0 688 100- 02	Peroxidase (EC 1.11.1.7).	1 3 4 9	gi|3834550 6|emb|CAE 01785.2| OSJNBa00 39K24.4	318	91.90966	175	40.08711	2. 2 9 2 7 4 9	1.1970 78	2.08E- 17	1. 5 3 E- 1 9
OS1	Similar to 40S ribosomal protein S16.	7	gi|1154871	664	363.6085	387	167.9614	2.	1.1142	1.77E-	5.

2T0124200-01		12	04|ref|NP_001066039.1|Os12g0124200					1648333	56	32	61E-35
OS10T0408700-02	Similar to GAMYB-binding protein (Fragment).	13116	hypothetical protein OsJ_05402	34	10.07324	12	2.81776	3.57491	1.837907	0.000849	4.86E-05
OS0	Hypothetical conserved gene.	1	unknown	234	52.85926	136	24.34875	2.	1.1183	1.52E-	2.

5T0208500-03		726	protein, contains 2OG-Fe(II) oxygenase superfamily					170923	08	11	05E-13
OS01T0756900-01	Hypothetical protein.	6640	gi|20161218|dbj|BAB90145.1| unknown protein	90	54.82877	40	19.31339	2.838899	1.505331	3.91E-07	9.79E-09

OS11T0297000-01	Conserved hypothetical protein.	11122	hypothetical protein OsJ_33690	57	19.80742	27	7.436173	2.66636658	1.413409	0.000273	1.34E-05
OS07T0677300-01	Peroxidase.	1244	gi|303851|dbj|BAA03911.1| peroxidase	76	23.81986	34	8.445729	2.82034	1.495871	5.00E-06	1.56E-07

OS03T0293100-01	Conserved hypothetical protein.	963	gi|113548280|dbj|BAF11723.1| Os03g0293100	27	10.93159	7	2.24621	44.866684	2.282939	0.000579	3.14E-05
OS07T0529700-01	Similar to Xyloglucan endotransglucosylase/hydrolase 1.	17706	gi|125558604|gb|EAZ04140.1| hypothetical protein	41	9.370242	12	2.173606	4.31009	2.107996	2.82E-05	1.05E-0

OS02T0670900-02	Similar to amino acid transport protein.	2055	OsI_26283 gi|50251349|dbj|BAD28325.1| putative amino acid transport protein	131	24.85452	72	10.82678	22.295653	1.198904	2.60E-07	66.23E-09
OS07T0287400-01	Plant lipid transfer protein/seed storage/trypsin-alpha amylase inhibitor domain containing protein.	730	gi|23237849|dbj|BAC16424.1| unknown protein	253	135.1275	115	48.68034	2.7758	1.47291	4.72E-19	3.06E-2

OS01T0153300-00	Similar to Histone H2B.1.	712	gi|13872950|dbj|BAB44055.1| putative histone H2B	261	142.9244	161	69.87543	122.04545418	1.032396	2.61E-11	13.61E-13
OS08T0300300-	Similar to Chloride channel-like protein Osclc.	2694	gi|34015377|gb|AAQ56565.1| putative	100	14.47266	37	4.244072	3.4410	1.769809	6.86E-10	1.14E-

01			chloride channel protein					089			11
OS01T0238500-01	Similar to Branched-chain-amino-acid aminotransferase-like protein 3, chloroplast precursor.	1518	gi|56784110|dbj|BAD81481.1| branched-chain amino acid aminotransferase -like	92	23.62991	50	10.17834	2.3215888	1.215112	2.34E-05	8.59E-07
OS04T0	Similar to OSIGBa0075F02.3 protein.	550	gi|32487809|emb|CAE	89	68.85024	53	32.49555	2.1	1.083219	0.000215	1.0

387600-01		4	05527.1|OSJNBa0053B21.1					18759			1E-05
OS06T0614400-01	Conserved hypothetical protein.	585	no hit	124	82.64409	26	13.73397	6.017495	2.589163	1.37E-19	8.41E-22
OS0	Similar to Gibberellin-stimulated	4	gibberellin-	111	99.9496	55	39.25124	2.	1.3484	1.97E-	4.

6T0266800-01	protein.	33	stimulated protein					546406	63	07	58E-09
OS08T0112950-00	Hypothetical protein.	888	unknown	612	268.7104	279	97.08896	2.767672	1.468673	3.95E-46	8.27E-49

OS0 3T0 288 000- 02	Similar to Metallothionein.	4 1 3	gi|1087075 76|gb|ABF9 5371.1| Metallothio nein-like protein 1, putative, expressed	861	812.8288	407	304.525	2. 6 6 9 1 6 9	1.4163 91	1.44E- 61	1. 9 5 E- 6 4
OS0 3T0 259 500- 00	Similar to Ubiquitin.	6 7 8	gi|1607068 14|gb|EAT8 6934.2| hypothetical protein	59	33.92878	10	4.557733	7. 4 4 4 2	2.8961 21	1.48E- 10	2. 2 3 E- 1

			SNOG_058 70					2 3			2
OS05T0541750-01	Hypothetical gene.	431	gi|162280751|gb|ABX83036.1| arabinogalactan peptide 3	242	218.9193	129	92.4892	2.366972	1.243042	4.49E-14	4.44E-16
OS03T0670700-	Similar to Glycine-rich RNA-binding, abscisic acid-inducible protein.	781	gi|108710321|gb|ABF98116.1| retrotransp	4227	2110.217	2063	816.2568	2.5585	1.370297	########	####

01			oson<br>protein,<br>putative,<br>Ty1-copia<br>subclass,<br>expressed					2<br>3<br>7			#<br>#<br>#<br>#
OS0<br>9T0<br>420<br>300-<br>01	Methyladenine glycosylase domain containing protein.	1<br>6<br>4<br>8	gi|5025249<br>1|dbj|BAD2<br>8669.1| putative<br>chain A,<br>solution<br>structure of	47	11.11954	18	3.375156	3.<br>2<br>9<br>4<br>5<br>2<br>4	1.7200<br>7	0.0001<br>17	5.<br>0<br>6<br>E-<br>0<br>6

			3-methyladenine DNA glycosylase I (Tag)								
OS08T0416000-02	Similar to Homeobox-leucine zipper protein HOX5.	1271	gi|5006855|gb|AAD37698.1|AF145729_1 homeodomain leucine zipper protein	77	23.62061	44	10.69758	2.208032	1.142761	0.000355	1.81E-05
OS0	Similar to laccase 90c.	2	gi|2556744	87	16.93496	42	6.479581	2.	1.3860	3.80E-	1.

3T0297900-00		003	36|dbj|BAF11755.2|Os03g0297900					613589	32	06	16E-07
OS02T0256200-02	Similar to Histidine-rich protein.	1302	gi|50251677|dbj|BAD29701.1|putative histidine-rich protein	456	136.5526	277	65.74268	2.077076	1.054554	2.43E-20	1.41E-22

OS05T0134400-02	Similar to peroxidase 1.	16600	gi|55700997|tpe|CAH69307.1| TPA: class III peroxidase 65 precursor	217	52.8793	111	21.43787	2.4666631	1.302542	1.42E-13	1.46E-15
OS05T0217000-01	Protein of unknown function DUF1070 family protein.	4590	gi|48843764|gb|AAT47023.1| unknown protein	61	51.81591	22	14.81114	3.449884	1.806712	2.59E-06	7.54E-40

OS04T0429450-01	Conserved hypothetical protein.	1859	hypothetical protein OsI_15922	405	84.94183	249	41.3903	412.052216	1.037183	1.37E-17	89.90E-20
OS12T0555100-	Similar to Probenazole-inducible protein PBZ1.	848	gi|108862810|gb|ABA99547.2| pathogenes	25	11.4945	6	2.186422	5.257	2.3943	0.000682	3.78E-

01			is-related protein 10, putative, expressed					22			05
OS11T0100300-00	Conserved hypothetical protein.	25996	gi|77548278|gb|ABA91075.1| expressed protein	139	20.87642	42	4.999461	4.17757355	2.06203	9.66E-17	7.770E-19
OS03T0	Similar to Histone H2A.	81	gi|22773254|gb|AAN0	117	56.11013	51	19.38466	2.8	1.533346	1.95E-09	3.4

162200-01		3	6860.1| Putative histone H2A					94563			1E-11
OS07T0126500-00	Allergen V5/Tpx-1 related family protein.	677	gi|28201315|dbj|BAC56823.1| putative pathogenesis-related protein	246	141.6747	117	53.40425	2.6552874	1.407556	2.60E-17	1.94E-19
OS0	Similar to loricrin.	5	gi|5790043	72	50.4898	39	21.67546	2.	1.2199	0.0002	1.

1T0916100-01		56	2|dbj|BAD87725.1| unknown protein					3293533	29	59	26E-005
OS09T0553900-01	Conserved hypothetical protein.	30224	gi|113632117|dbj|BAF25798.1| Os09g0553900	42	5.415187	16	1.634996	3.3312049	1.727724	0.000305	1.52E-005

OS07T0549900-00	Similar to Histone H4.	502	TPA: histone H4.3	139	107.9586	58	35.70285	3.023808	1.596366	7.89E-12	1.01E-13
OS03T0204366-00	Conserved hypothetical protein.	603	gi|255670716|dbj|BAH91584.1| Os02g0212975	91	58.83965	27	13.83646	4.252250	2.088314	3.44E-11	4.81E-13

								7			
OS04T0642000-02	Similar to H0423H10.7 protein.	1667	gi|38344910|emb|CAD41854.2| OSJNBb0079B02.13	98	22.92115	57	10.56618	2.1692 95	1.117226	5.15E-05	2.03E-06
OS06T0202400-01	Conserved hypothetical protein.	8664	gi|51091002|dbj|BAD35604.1| unknown protein	42	18.95316	17	6.080143	3.11172	1.640261	0.000564	3.05E-0

								22			5
OS05T0512600-01	X8 domain containing protein.	1016	gi|48475080|gb|AAT44149.1| unknown protein	131	50.2717	52	15.81569	3.178596	1.66839	7.10E-12	9.055E-14
OS04T0414700-	Photosystem I PsaO domain containing protein.	645	gi|32479727|emb|CAE01514.1| OJ991214_	92	55.61272	43	20.60095	2.6699	1.432704	8.58E-07	2.330E-

01			12.3					521			08
OS04T0250050-00	Conserved hypothetical protein.	1249	gi|113564001|dbj|BAF14344.1|Os04g0309500	29	9.052771	8	1.979275	4.5737781	2.193387	0.000482	2.56E-05
OS09T0369	Similar to DRE binding factor 2.	899	gi|49387678|dbj|BAD25924.1|	347	150.4928	185	63.59027	2.36	1.242817	3.85E-20	2.26

050-01			DRE binding factor 2					6601			E-22
OS11T0546750-00	Hypothetical gene.	762	gi|223974951|gb|ACN31663.1| unknown	46	23.53688	20	8.110612	2.9019855	1.53704	0.000579	3.14E-05
OS05T0	Hypothetical conserved gene.	24	gi|158937152|dbj|BAF	140	227.4379	75	96.56697	2.3	1.235871	3.63E-08	7.6

514800-00		0	91631.1| CLE family OsCLE509 protein					55235			3E-10
OS03T0286500-01	Similar to RNA-binding region containing protein 1 (HSRNASEB) (ssDNA binding protein SEB4) (CLL-associated antigen KW-5). Splice isoform 2.	1378	gi|108707565|gb|ABF95360.1| RNA recognition motif family protein, expressed	81	22.91827	29	6.503204	3.52415	1.817275	2.41E-08	4.91E-10

OS05T0563600-01	FAS1 domain domain containing protein.	11102	gi|50511474|gb|AAT77396.1| unknown protein	231	81.72904	105	29.44329	2.77758123	1.47291	2.11E-17	1.57E-19
OS06T0346201-00	Hypothetical conserved gene.	627	gi|255673370|dbj|BAH91152.1| Os01g0563500	25	15.54599	6	2.957075	5.257222	2.3943	0.000682	3.78E-05

OS03T0799000-01	Similar to Histone H1.	1386	gi|28209501|gb|AAO37519.1| histone-like protein	531	149.3748	261	58.19101	2.566974	1.360068	1.48E-35	4.16E-38
OS05T0477300-03	Ribosomal protein S26e domain containing protein.	946	putative 40S ribosomal protein S26	1004	413.7982	611	199.5854	2.07328	1.051921	7.34E-45	1.63E-47

								9			
OS05T0556900-02	Similar to 60S ribosomal protein l33-b (Fragment).	311	gi|49328003|gb|AAT58704.1| putative ribosomal protein L35A	102	127.875	59	58.6233	2.181301	1.125189	2.94E-05	1.099E-06
OS09T0319701-01	Conserved hypothetical protein.	922	gi|255678769|dbj|BAH94495.1| Os09g0319701	97	41.01916	53	17.7633	2.3092	1.207399	1.43E-05	4.996E-0

								09			7
OS02T0779200-01	Similar to Subtilisin-like protease (Fragment).	26552	gi|47497462|dbj|BAD19517.1| putative subtilisin-like proteinase	80	11.76149	42	4.893892	2.4033301	1.265017	5.49E-05	2.177E-06
OS02T0690000-	Conserved hypothetical protein.	5466	gi|24413950|dbj|BAC22202.1| hypothetical	45	32.13408	18	10.18729	3.154	1.657334	0.000279	1.337E-

01			protein					332			05
OS01T0878700-02	Amino acid transporter, transmembrane domain containing protein.	17554	gi|56784616|dbj|BAD81663.1| putative amino acid carrier	51	11.3367	18	3.171185	3.57491	1.837907	1.83E-05	6.52E-07
OS03T0120400-	Heavy metal transport/detoxification protein domain containing protein.	1130	gi|21426116|gb|AAM52313.1|AC105363_2	116	40.02446	69	18.86902	2.1121	1.084863	1.31E-05	4.50E-

01			Unknown protein					174			07
OS06T0160000-00	Hypothetical protein.	431	unknown	46	41.61276	13	9.320617	4.464593	2.158529	4.90E-06	1.53E-07
OS09T0483	Similar to Ubiquitin/ribosomal fusion protein (Fragment).	781	no hit	167	83.37031	14	5.539309	15.0	3.911756	3.22E-238	8.23

400-01								5067			E-41
OS03T0244950-00	Heat shock protein DnaJ, N-terminal domain containing protein.	516	gi|255674362|dbj|BAH92072.1|Os03g0244950	33	24.93505	11	6.587515	3.7855198	1.920369	0.000679	3.75E-05
OS12T0	Conserved hypothetical protein.	47	gi|255670288|dbj|BAH	390	323.5286	144	94.67673	3.4	1.772812	1.78E-38	4.5

466200-01		0	95668.1|Os12g0466550, partial					17193			0E-41
OS04T0550866-00	Hypothetical protein.	623	unknown	60	37.54994	24	11.90424	3.1543332	1.657334	1.48E-05	5.14E-07
OS0	Similar to Peroxidase (EC 1.11.1.7).	1	gi|2226376	115	36.48312	49	12.32034	2.	1.5661	1.46E-	2.

7T0676900-01		229	81|gb|EEE67813.1| hypothetical protein OsJ_25567					96121	87	09	50E-11
OS12T0292301-00	Hypothetical gene.	736	no hit	56	29.66581	25	10.49641	2.826281	1.498905	0.000146	6.51E-06
OS0	Hypothetical conserved gene.	8	gi|2556770	68	30.30029	24	8.475821	3.	1.8379	3.69E-	9.

<table>
<tr><td>3T0
789
433-
01</td><td></td><td>7
5</td><td>85|dbj|BAH
93540.1|
Os06g0507
101, partial</td><td></td><td></td><td></td><td></td><td>5
7
4
9
1</td><td>07</td><td>07</td><td>2
0
E-
0
9</td></tr>
<tr><td>OS1
0T0
330
400-
01</td><td>Protein of unknown function DUF179
family protein.</td><td>1
1
2
6</td><td>gi|1690521
0|gb|AAL31
080.1|AC09
1749_9
unknown
protein</td><td>47</td><td>16.27442</td><td>15</td><td>4.116532</td><td>3.
9
5
3
4
2
9</td><td>1.9831
05</td><td>1.44E-
05</td><td>5.
0
2
E-
0
7</td></tr>
<tr><td>OS0</td><td>Similar to Type III chlorophyll</td><td>9</td><td>gi|3439338</td><td>33</td><td>13.23712</td><td>7</td><td>2.225412</td><td>5.</td><td>2.5724</td><td>2.23E-</td><td>8.</td></tr>
</table>

7T0	a/b-binding protein (Fragment).	7	4|dbj|BAC8					9	46	05	1
562		2	3393.1|					4			4
700-			putative					8			E-
02			chlorophyll					1			0
			A-B binding					6			7
			protein of					9			
			LHCII type								
			III,								
			chloroplast								
			precursor								
			(CAB)								
OS0	Similar to Tubulin alpha-1 chain	1	gi|1044101					2.	1.2464	2.50E-	5.
				739	165.2129	393	69.63453				
3T0	(Alpha-1 tubulin).	7	6|gb|AAG1					3	51	43	8

726<br>100-<br>01		4<br>4	6905.1|AF1<br>82523_1<br>alpha-tubuli<br>n					7<br>2<br>5<br>7<br>1			4<br>E-<br>4<br>6
OS0<br>5T0<br>423<br>500-<br>01	Protein kinase, core domain containing protein.	2<br>1<br>6<br>2	gi|5398085<br>0|gb|AAV24<br>771.1| unknow<br>protein	46	8.295606	21	3.001527	2.<br>7<br>6<br>3<br>7<br>9<br>6	1.4666<br>51	0.0009<br>85	5.<br>7<br>9<br>E-<br>0<br>5
OS0	Kelch related domain containing	1	gi|3834525	131	32.84633	63	12.51955	2.	1.3915	3.79E-	6.

4T0482300-01	protein.	555	7|emb|CAD41101.2| OSJNBb0011N17.18					623603	49	09	90E-11
OS04T0465100-01	Haem peroxidase, plant/fungal/bacterial family protein.	13551	gi|32489883|emb|CAE04363.1| OSJNBa0060P14.16	63	18.18156	31	7.090632	2.564167	1.35849	0.000194	8.94E-06

OS10T0554800-01	Plant lipid transfer protein and hydrophobic protein, helical domain containing protein.	800	predicted protein	125	60.92086	73	28.19756	2.16050501	1.111366	3.08E-06	9.20E-08
OS08T0155550-00	Hypothetical protein.	16998	unknown	64	14.69563	27	4.913655	2.99077	1.580519	1.54E-05	5.40E-07

OS08T0389700-02	Protein of unknown function DUF819 family protein.	1922	gi|40253412|dbj|BAD05341.1| membrane protein-like	78	15.82294	43	6.913432	42.288872254	1.194544	0.000172	7.800E-006
OS01T0866200-00	Similar to Histone H3.	585	unnamed protein product	234	155.9574	134	70.78277	2.22033	1.139682	6.90E-12	8.775E-1

								2<br>4			4
OS0<br>3T0<br>670<br>700-<br>03	Similar to Glycine rich RNA binding protein.	7<br>0<br>2	gi|1087103<br>20|gb|ABF9<br>8115.1| retrotransp<br>oson<br>protein,<br>putative,<br>Ty1-copia<br>subclass,<br>expressed	4203	2334.362	2008	883.9042	2.<br>6<br>4<br>0<br>9<br>6<br>8	1.4010<br>67	#####<br>###	#<br>#<br>#<br>#<br>#<br>#<br>#<br>#
OS0	Similar to predicted protein.	1	gi|1135482	75	16.73842	40	7.075314	2.	1.2422	0.0001	6.

3T0291800-02		747	71|dbj|BAF11714.1| Os03g0291800, partial					365749	97	39	14E-06
OS01T0874300-01	Similar to MybHv5 (Fragment).	1033	gi|19386839|dbj|BAB86217.1| putative myb-related protein	73	27.55298	30	8.974278	3.070216	1.61834	1.83E-06	5.19E-08

OS05T0395400-00	Hypothetical protein.	5992	gi|113579014|dbj|BAF17377.1| Os05g0395400	64	42.15065	10	5.219836	8.0750 9	3.013478	7.09E-12	9.006E-14
OS03T0223000-01	Similar to atypical receptor-like kinase MARK.	15565	hypothetical protein OsI_10572	90	22.42199	52	10.26757	2.1837768	1.12682	0.000106	4.57E-06

OS05T0429100-02	Hypothetical protein.	15996	gi|215712294|dbj|BAG94421.1| unnamed protein product	99	24.18512	27	5.227686	4.6263553	2.209876	5.01E-13	5.52E-15
OS04T0384000-01	Hypothetical conserved gene.	4551	hypothetical protein OsJ_14542	30	25.93527	7	4.796231	5.40742	2.434942	0.000118	5.15E-06

OS05T0192700-01	Conserved hypothetical protein.	541	gi|255676104|dbj|BAH92986.1| Os05g0192700	184	132.607	56	31.98669	64.145693	2.051613	5.37E-22	2.83E-24
OS02T0198500-02	Hypothetical conserved gene.	617	gi|215686577|dbj|BAG88830.1| unnamed protein	67	42.33852	37	18.53084	2.2847	1.192042	0.000644	3.53E-0

			product					59			5
OS02T0332700-01	Conserved hypothetical protein.	1018	gi|113536258|dbj|BAF08641.1| Os02g0332700, partial	69	26.42697	27	8.195861	3.224428	1.689043	1.71E-06	4.84E-08
OS08T0469600-	Conserved hypothetical protein.	621	gi|42407414|dbj|BAD09572.1| unknown	30	18.83543	7	3.483253	5.4407	2.434942	0.000118	5.115E-

01			protein					426			06
OS10T0412700-01	Thaumatin, pathogenesis-related family protein.	1007	gi|31432058|gb|AAP53743.1| Thaumatin family protein, expressed	35	13.55141	12	3.682395	3.6880054	1.879727	0.000532	2.85E-05
OS08T0112	Lipase, GDSL domain containing protein.	1132	gi|42408361|dbj|BAD09513.1|	41	12.05553	11	2.563467	4.70	2.233527	1.22E-05	4.15

900-01		6	putative GDSL-motif lipase/hydrolase protein					2822			E-07
OS05T0462700-01	Similar to Histone H4.	5590	TPA: histone H4.3	89	58.81444	26	13.61758	4.3190008	2.1107	4.28E-11	6.005E-13
OS0	Hypothetical protein.	1	no hit	84	18.08451	30	5.118956	3.	1.8208	1.16E-	2.

8T0468801-01		811						5328552	33	08	23E-10
OS12T0603700-00	Similar to CBL-interacting protein kinase 4.	13220	gi|113649721|dbj|BAF30233.1|Os12g0603700	41	12.11033	15	3.511526	3.4487336	1.786068	0.000253	1.222E-05

OS07T0638400-01	Similar to 1-Cys peroxiredoxin.	1047	gi|27261085|dbj|BAC45198.1| putative RAB24 protein	220	81.92604	130	38.36854	2.1352524	1.094398	1.70E-10	2.57E-122
OS04T0497700-01	Similar to CONSTANS-like protein.	13394	gi|38345390|emb|CAE03116.2| OSJNBa0067K08.19	70	19.57858	21	4.655165	4.2205776	2.072372	1.41E-08	2.78E-10

OS04T0320700-02	UDP-glucuronosyl/UDP-glucosyltransferase domain containing protein.	1639	gi|38344780|emb|CAE01506.2|OSJNBb0026L04.11	68	16.17618	34	6.410303	2.523466	1.335406	0.000121	5.26E-06
OS10T0413101-01	Similar to H0815C01.6 protein.	1842	hypothetical protein OsI_16109	117	24.76522	46	7.71697	3.20919	1.682209	9.43E-11	1.38E-12

OS01T0280500-01	Similar to Eukaryotic translation initiation factor 6 (eIF-6) (B4 integrin interactor) (CAB) (p27(BBP)) (B(2)GCN homolog).	1161	gi|56783682|dbj|BAD81094.1| putative integrin beta 4 binding protein	21	7.052337	0.5	0.133081	52.99927 8	5.727724	1.17E-05	3.977E-077
OS02T0173000-01	Similar to UreD.	27745	unnamed protein product	105	14.91396	59	6.641838	2.22454	1.167009	1.06E-05	3.555E-0

								57			7
OS06T0160100-00	Similar to Histones H3 and H4 (ISS).	890	PREDICTED: uncharacterized protein LOC100903667	145	63.52197	68	23.61008	2.69046	1.427853	1.72E-10	2.61E-12
OS06T0240001-01	Conserved hypothetical protein.	1954	gi|215769111|dbj|BAH01340.1| unnamed protein	65	12.96984	28	4.428046	2.9290	1.550419	1.79E-05	6.35E-0

			product					23			7
OS03T0241600-01	Serine/threonine protein kinase-related domain containing protein.	2787	gi|125585562|gb|EAZ26226.1| hypothetical protein OsJ_10094	90	12.59075	52	5.765606	2.183768	1.12682	0.000106	4.57E-06
OS11T0247350-	Hypothetical gene.	482	gi|7268241|emb|CAB78537.1| hypothetical	251	203.0358	133	85.26744	2.381	1.251668	9.38E-15	8.75E-

00			protein					165			17
OS06T0156600-01	Lipase, GDSL domain containing protein.	1429	gi|55296702|dbj|BAD69420.1| putative lipase	136	37.10673	64	13.83969	2.6811182	1.422869	8.29E-10	1.39E-11
OS05T0510	Similar to Cellulose synthase-like family C1 protein.	2556	gi|48475069|gb|AAT44138.1|	82	12.48878	45	5.431892	2.229	1.201105	9.75E-05	4.115

800-01		0	putative glucosyltransferase					9157			E-06
OS04T0208200-01	Cysteine proteinase.	13447	putative cysteine protease	95	27.49806	24	5.505823	4.994359	2.320299	2.96E-13	3.14E-15
OS02T0	Hypothetical gene.	11	no hit	30	10.58534	8	2.237208	4.7	2.242297	0.000288	1.4

326<br>100-<br>01		0<br>5						3<br>1<br>4<br>9<br>8			2<br>E-<br>0<br>5
OS1<br>1T0<br>707<br>000-<br>03	Similar to Ribulose bisphosphate carboxylase/oxygenase activase, chloroplastic.	1<br>4<br>7<br>0	gi|6273316<br>9|gb|AAX95<br>286.1| RuBisCO<br>activase<br>small<br>isoform<br>precursor	166	44.02879	97	20.39074	2.<br>1<br>5<br>9<br>2<br>5<br>4	1.1105<br>33	3.63E-<br>08	7.<br>6<br>4<br>E-<br>1<br>0

OS04T0450300-00	Similar to H0818E04.18 protein.	10200	gi|116310080|emb|CAH67101.1| H0818E04.18	40	15.28994	15	4.544328	3.364621	1.750444	0.000394	2.04E-05
OS03T0861400-00	Hypothetical conserved gene.	11180	gi|31193908|gb|AAP44743.1| putative heavy-metal-associated	102	33.70266	43	11.26069	2.99294	1.581567	1.26E-08	2.46E-10

			protein					8			
OS03T0288000-01	Similar to Metallothionein.	751	gi|108707576|gb|ABF95371.1| Metallothionein-like protein 1, putative, expressed	1468	762.1353	902	371.1464	2.053463	1.038059	2.67E-64	3.28E-67
OS07T0154300-	Conserved hypothetical protein.	583	gi|50508821|dbj|BAD31594.1| unknown	85	56.84553	40	21.20167	2.6681	1.422869	3.06E-06	9.12E-

01			protein					182			08
OS07T0498400-00	Leucine-rich repeat, typical subtype domain containing protein.	3828	gi|255677788|dbj|BAF21624.2|Os07g0498400	82	8.35195	21	1.69522	4.926766	2.300641	2.28E-11	3.14E-13
OS04T0495	DOMON domain containing protein.	1886	gi|38345432|emb|CAD41554.2|	69	14.43275	35	5.802307	2.448	1.314648	0.000133	5.884

400-01		4	OSJNBb0091E11.23					7416			E-06
OS10T0493300-01	Hypothetical protein.	1502	gi|10140703|gb|AAG13537.1|AC023240_10 unknown protein	230	59.70406	131	26.95132	2.215256	1.147473	8.09E-12	1.05E-13
OS03T0	Phospholipase A2 family protein.	89	gi|4585708|emb|CAB4	77	33.58143	39	13.48049	2.4	1.316791	4.24E-05	1.6

261100-01		4	0842.1| putative phospholipase A2					91113			4E-06
OS04T0252850-00	Hypothetical gene.	687	no hit	179	101.588	87	39.13282	2.5959579	1.376279	3.27E-12	4.00E-14
OS0	Harpin-induced 1 domain containing	1	gi|3834403	173	64.11747	52	15.27447	4.	2.0695	6.60E-	3.

4T0416700-01	protein.	052	6|emb|CAE01528.2|OJ991214_12.17					197688	95	21	68E-23
OS11T0256900-01	SAM dependent carboxyl methyltransferase family protein.	13667	gi|255679967|dbj|BAH95192.1|Os11g0256900	69	19.68007	33	7.459746	2.638169	1.399537	5.05E-05	1.99E-06

OS12T0425800-01	Hypothetical protein.	9200	gi|77555147|gb|ABA97943.1| expressed protein	210	88.99743	122	40.97799	2.17718835	1.118915	2.16E-10	3.336E-112
OS01T0856500-01	Auxin transporter, Primary root and root hair elongation, Cd stress response	2405	gi|56785353|dbj|BAD82311.1| putative AUX1-like permease	82	13.29367	30	3.854648	3.444873	1.786068	2.99E-08	6.17E-110

								6			
OS03T0722200-01	Hypothetical protein.	612	gi|113549580|dbj|BAF13023.1| Os03g0722200, partial	38	24.20907	15	7.57388	3.19639	1.676443	0.000961	5.62E-05
OS05T0531200-02	Pollen Ole e 1 allergen and extensin domain containing protein.	9937	gi|45642728|gb|AAS72356.1| unknown protein	65	27.04704	24	7.914988	3.41719	1.772812	1.53E-06	4.27E-08

OS02T0575000-01	Conserved hypothetical protein.	627	gi|50725820|dbj|BAD33350.1| unknown protein	250	155.4599	157	77.37679	32.009129	1.00657	2.08E-10	3.22E-12
OS07T0545300-01	Similar to Histone H2A.	822	gi|34393263|dbj|BAC83133.1| histone H2A	108	51.22688	65	24.43544	2.0964	1.067926	3.99E-05	1.53E-0

								18			6
OS06T0565100-00	Cytochrome P450 family protein.	1662	gi|53793263|dbj|BAD54486.1| putative thromboxane-A synthase	101	23.69389	50	9.29646	2.54487	1.349762	8.27E-07	2.20E-08
OS03T0749401-	Hypothetical protein.	1812	no hit	60	12.91038	32	5.457207	2.3655	1.242297	0.000906	5.25E-

00								749			05
OS03T0239300-01	Zinc finger, C2H2-type domain containing protein.	1266	gi|108707079|gb|ABF94874.1| Zinc finger, C2H2 type family protein, expressed	59	18.17039	28	6.83444	2.658651	1.410695	0.000209	9.74E-06
OS01T0	Harpin-induced 1 domain containing protein.	10	gi|20521395|dbj|BAB9	80	30.40105	40	12.04734	2.5	1.335406	2.16E-05	7.8

574800-01		26	1906.1| hypothetical protein					23466			3E-07
OS01T0186950-00	Hypothetical protein.	1074	hypothetical protein ZEAMMB73_325160	24	8.712704	4	1.150891	7.570397	2.920369	0.000155	6.94E-06
OS0	Similar to Histone H4.	7	TPA:	275	151.4417	137	59.79514	2.	1.3406	5.84E-	4.

1T0835900-00		08	histone H4.3					532675	62	18	08E-220
OS10T0554200-02	Nitrate transporter, Nitrate uptake, Nitrate transport	21448	gi|215687052|dbj|BAG90898.1| unnamed protein product	40	7.260586	16	2.301783	3.154332	1.657334	0.000739	4.14E-05

OS11T0475500-01	Conserved hypothetical protein.	904	expressed protein	61	26.30918	30	10.2549	2.565523	1.359253	0.000255	1.23E-05
OS08T0503000-01	Hypothetical protein.	8227	gi|218201408|gb|EEC83835.1| hypothetical protein OsI_29786	343	161.7092	125	46.70712	3.46219	1.791687	2.55E-34	7.68E-37

OS12T0171500-01	Conserved hypothetical protein.	10190	no hit	17	6.080908	2	0.566999	510.72473	3.422869	0.000841	4.81E-05
OS08T0524400-00	Protein of unknown function DUF568, DOMON-like domain containing protein.	9953	gi|29647484|dbj|BAC75413.1| putative auxin-induc	76	31.09329	26	8.430611	3.68881	1.882894	3.20E-08	6.67E-1

			ed protein					42			0
OS06T0319366-00	Hypothetical genes.	2225	unknown protein	87	150.7588	46	63.17626	2.386321	1.254788	2.50E-05	9.19E-07
OS04T0459200-	Hypothetical protein.	7077	no hit	113	62.31678	47	20.54268	3.033	1.600996	1.06E-09	1.80E-

01								528			11
OS05T0429100-01	Hypothetical protein.	1645	gi|215712294|dbj|BAG94421.1| unnamed protein product	97	22.99068	40	7.514026	3.059702	1.613391	1.91E-08	3.80E-100
OS07T0589	GRAS transcription factor domain containing protein.	217	hypothetical protein OsJ_24947	58	10.42112	29	4.129684	2.52	1.335406	0.00049	2.60

200-00		0						3466			E-05
OS02T0538700-01	Harpin-induced 1 domain containing protein.	10076	gi|50251988|dbj|BAD27922.1| harpin-induced protein-like	47	17.03066	20	5.743761	2.965072	1.568067	0.000381	1.96E-05
OS01T0	Hypothetical protein.	20	no hit	484	90.63806	159	23.59908	3.8	1.941387	4.03E-54	6.0

357150-00		82						40746			8E-57
OS07T0639000-01	Similar to Class III peroxidase 46.	1230	gi|27261094|dbj|BAC45207.1| putative peroxidase	140	44.37812	61	15.3251	2.89578	1.533952	2.96E-11	4.11E-13
OS01T0	TGF-beta receptor, type I/II extracellular region family protein.	17	gi|56784227|dbj|BAD8	78	16.98029	44	7.591642	2.2	1.161377	0.000255	1.2

871500-01		91	1722.1| putative oligopeptide transporter					36708			3E-05
OS03T0364400-02	Similar to Phytosulfokine receptor-like protein.	17667	gi|108708318|gb|ABF96113.1| Protein kinase APK1A, chloroplast precursor,	50	11.03264	23	4.022258	2.742897	1.455701	0.000565	3.06E-05

			putative, expressed								
OS05T0555800-00	Similar to 60S ribosomal protein L35a-3.	339	gi|49328003|gb|AAT58704.1| putative ribosomal protein L35A	174	200.1223	98	89.33157	2.2402219	1.16364	3.42E-09	6.17E-111
OS09T0369400-	Similar to predicted protein.	1549	RecName: Full=Probable trehalose-p	35	8.80973	11	2.194421	4.01214	2.005258	0.000258	1.25E-

01			hosphate<br>phosphatas<br>e 7;<br>Short=OsT<br>PP7;<br>AltName:<br>Full=Trehal<br>ose<br>6-phosphat<br>e<br>phosphatas<br>e					6<br>0<br>4			0<br>5
OS0	DREPP plasma membrane	1	gi|4784771	38	13.71847	14	4.005741	3.	1.7759	0.0005	2.

2T0285300-01	polypeptide family protein.	080	2|dbj|BAD21491.1| putative DREPP2 protein					424703	79	12	73E-05
OS03T0809000-01	Plant disease resistance response protein family protein.	862	gi|50540750|gb|AAT77906.1| putative dirigent-like protein	25	11.30782	5	1.792426	6.308664	2.657334	0.000257	1.24E-05

OS01T0763900-01	X8 domain containing protein.	9954	gi|57899559|dbj|BAD87138.1| glycosyl hydrolase family protein 17-like	78	31.87808	33	10.68917	2.982277	1.576414	1.22E-06	3.35E-08
OS11T0703900-01	Heat shock protein 70.	23779	gi|62733235|gb|AAX95352.1| dnaK-type molecular	8673	1421.415	5413	703.1082	2.0216	1.015509	0	0

			chaperone hsp70 - rice (fragment)					16			
OS06T0257450-00		1926	gi|52077275|dbj|BAD46317.1| putative ribonucleotide reductase R2	77	15.58764	40	6.417743	2.428836	1.280265	6.72E-05	2.73E-006
OS03T0	Similar to Photosystem II type II chlorophyll a/b binding protein	11	gi|50839002|gb|AAT81	88	31.05034	25	6.991274	4.4	2.150982	3.12E-11	4.3

592500-01	(Fragment).	05	763.1| chlorophyll a/b binding protein					41299			3E-13
OS08T0510700-01	Homeodomain-like containing protein.	2518	gi|28411872|dbj|BAC57402.1| DNA-binding protein family-like	147	22.76185	74	9.081437	2.506415	1.325625	1.61E-09	2.78E-11
OS0	Similar to 30S ribosomal protein S16.	6	gi|1136317	80	47.76643	42	19.87535	2.	1.2650	5.49E-	2.

9T0498100-01		53	97|dbj|BAF25478.1| Os09g0498100					403301	17	05	17E-06
OS10T0552700-01	Similar to Tumor-related protein (Fragment).	627	gi|10140655|gb|AAG13491.1|AC026758_28 putative lipid transfer	27	16.78967	6	2.957075	5.677797	2.505331	0.000233	1.10E-05

			protein								
OS11T0171300-01	Fructose-bisphosphate aldolase, chloroplast precursor (EC 4.1.2.13) (ALDP).	1492	gi|62732953|gb|AAX95072.1| Fructose-bisphosphate aldolase class-I	508	132.7519	251	51.98565	2.55536266	1.352548	1.00E-33	3.08E-36
OS04T0496300-01	Similar to H0306B06.4 protein.	914	gi|38345379|emb|CAE03111.2| OSJNBa0067K08.8	91	38.81872	55	18.59495	2.08875	1.061841	0.000236	1.12E-0

								9<br>4			5
OS0<br>8T0<br>253<br>800-<br>01	Similar to Cellulose synthase-like family C3 protein (Fragment).	1<br>7<br>9<br>4	gi|1136233<br>48|dbj|BAF<br>23293.1| Os08g0253<br>800, partial	56	12.17059	27	4.650717	2.<br>6<br>1<br>6<br>9<br>2<br>7	1.3878<br>74	0.0004<br>01	2.<br>0<br>8<br>E-<br>0<br>5
OS0<br>2T0<br>798<br>200-	Zinc finger, RING/FYVE/PHD-type domain containing protein.	1<br>3<br>5<br>5	gi|5109131<br>9|dbj|BAD3<br>6054.1| RING-H2	456	131.2114	280	63.85536	2.<br>0<br>5<br>4	1.0390<br>13	7.51E-<br>20	4.<br>4<br>8<br>E-

01			zinc finger protein-like					822			22
OS11T01178800-01	Plant disease resistance response protein family protein.	756	gi|62734201|gb|AAX96310.1| At3g13650	92	47.44736	56	22.88995	2.072847	1.051613	0.000245	1.118E-05
OS01T0270	Similar to Cationic peroxidase isozyme 40K precursor.	139	gi|6721554|dbj|BAA89584.1|	50	14.02495	24	5.335499	2.62	1.3943	0.000935	5.44

300-02		0	putative cationic peroxidase isozyme 40K precursor					861			E-05
OS08T0532350-00	Hypothetical gene.	499	no hit	183	142.987	102	63.16525	2.2636697	1.178681	7.44E-10	1.24E-11

OS07T0617700-01	Cyclin-like F-box domain containing protein.	13992	gi|22831164|dbj|BAC16024.1| F-box protein family, AtFBX13-like protein	51	14.28489	23	5.10584	2.797775	1.48427	0.000379	1.94E-05
OS03T0345901-00	Hypothetical gene.	10333	no hit	211	79.63943	111	33.20483	2.3984	1.26209	1.38E-12	1.61E-1

								29			4
OS09T0470500-01	Homeodomain leucine zipper protein.	1461	gi|5006853|gb|AAD37697.1|AF145728_1 homeodomain leucine zipper protein	83	22.15001	29	6.133754	3.611166	1.852465	9.27E-09	1.766E-110
OS07T0573	Hypothetical conserved gene.	517	gi|255677903|dbj|BAF21985.2|	157	118.4009	51	30.48304	3.888	1.957602	1.18E-17	8.447

900-00			Os07g0573900, partial					4158			E-20
OS04T0539601-00	Hypothetical gene.	1448	no hit	54	14.54023	24	5.121784	2.838899	1.505331	0.000193	8.92E-06
OS10T0	Similar to Expansin-B4.	18	gi|14165334|gb|AAK55	59	12.21652	21	3.446256	3.5	1.825732	3.31E-06	9.9

556100-03		83	466.1|AC069300_21 beta-expansin (EXPB4)					44868			9E-008
OS11T0220700-01	Hypothetical protein.	773	gi|108864150|gb|ABA92100.2| expressed protein	103	51.95217	63	25.18487	2.0628833	1.044627	9.49E-05	4.01E-006
OS0	Similar to Exoglucanase precursor.	3	gi|1808767	145	18.27823	67	6.693812	2.	1.4492	1.01E-	1.

3T0749300-01		093	4|gb|AAL58966.1|AC091811_15 putative exoglucanase precursor					730616	26	10	49E-12
OS04T0495900-01	Hypothetical protein.	9667	gi|113564768|dbj|BAF15111.1| Os04g0495900	41	16.53116	16	5.112957	3.233319	1.692958	0.000476	2.541E-05

OS03T0119900-01	Similar to Histone H4.	526	TPA: histone H4.3	186	137.8711	73	42.88602	3.214826	1.684741	4.83E-17	3.76E-19
OS01T0127600-01	Similar to Bowman-Birk type proteinase inhibitor D-II precursor (IV).	878	gi|11275526|dbj|BAB18291.1| putative Bowman Birk trypsin	412	182.9569	190	66.87098	2.73596	1.452051	1.95E-30	6.89E-33

<table>
<tr><td></td><td></td><td></td><td>inhibitor</td><td></td><td></td><td></td><td></td><td>8</td><td></td><td></td><td></td></tr>
<tr><td>OS10T0452500-00</td><td>Similar to Glycine-rich protein precursor.</td><td>813</td><td>gi|31432427|gb|AAP54057.1| transposon protein, putative, CACTA, En/Spm sub-class, expressed</td><td>144</td><td>69.05863</td><td>49</td><td>18.62448</td><td>3.707949</td><td>1.890622</td><td>1.84E-15</td><td>1.57E-17</td></tr>
<tr><td>OS11T0</td><td>Alfa-tubulin.</td><td>17</td><td>gi|62734653|gb|AAX96</td><td>2647</td><td>588.0616</td><td>1184</td><td>208.4746</td><td>2.8</td><td>1.496096</td><td>########</td><td>##</td></tr>
</table>

247300-01		55	762.1| Tubulin/FtsZ family, GTPase domain, putative					20783			######
OS03T0755700-00	Similar to Ribosomal protein L13a.	687	putative ribosomal protein L13a	53	30.07912	19	8.546248	3.51957	1.815399	1.42E-05	4.92E-07
OS0	Similar to Histone H2A.	7	gi|1135780	120	59.00028	56	21.82194	2.	1.4349	8.84E-	1.

5T0113900-01		93	09|dbj|BAF16372.1| Os05g0113900					703713	42	09	67E-10
OS02T0832150-00	Similar to zinc finger (C3HC4-type RING finger) family protein.	592	RING zinc finger protein-like	34	22.39253	5	2.609918	8.5797783	3.100941	1.33E-06	3.666E-08

OS02T0258250-00	Hypothetical conserved gene.	6228	gi|50252043|dbj|BAD27975.1| putative embryogenic callus protein 98b	48	29.80078	19	9.349159	3.18753355	1.672441	0.00014	6.19E-06
OS01T0786500-00	Similar to early nodulin 20.	9004	gi|20804920|dbj|BAB92600.1| putative uclacyanin 3	66	28.46568	28	9.57124	2.97408	1.572446	1.18E-05	3.97E-07

OS02T0662000-01	RCc3 protein.	833	gi|786132|gb|AAA65513.1| RCc3	544	254.6243	290	107.58	42.3668337	1.24296	1.15E-31	3.82E-334
OS02T0583700-00	Conserved hypothetical protein.	630	gi|50253080|dbj|BAD29328.1| unknown protein	2057	1273.033	1043	511.5904	2.4883	1.315209	########	#####

								84			###
OS05T0474400-01	Prenylated rab acceptor PRA1 family protein.	10997	gi|52353676|gb|AAU44242.1| unknown protein	77	27.36718	40	11.26761	2.4288836	1.280265	6.72E-05	2.73E-06
OS03T0194	Hypothetical protein.	1400	predicted protein	24	6.655365	2	0.439565	15.1	3.920369	1.15E-05	3.888

350-<br>00		6						4<br>0<br>7<br>9			E-<br>0<br>7
OS0<br>1T0<br>738<br>000-<br>01	Conserved hypothetical protein.	5<br>0<br>9	gi|5790057<br>3|dbj|BAD8<br>7025.1| unknown<br>protein	84	64.34392	52	31.56924	2.<br>0<br>3<br>8<br>1<br>8<br>4	1.0272<br>84	0.0007<br>21	4.<br>0<br>1<br>E-<br>0<br>5
OS0<br>5T0	Similar to nodulin-like protein.	1<br>5	gi|5008032<br>0|gb|AAT69	234	58.78549	116	23.09643	2.<br>5	1.3477<br>9	2.03E-<br>15	1.<br>7

493800-01		52	654.1| putative nodulin MtN21 protein					4522			7E-17
OS02T0765900-00	Similar to Ferredoxin-nitrite reductase.	2060	putative ferredoxin--nitrite reductase, chloroplast precursor	34	6.435135	2	0.300014	21.44946	4.422869	2.10E-08	4.21E-10
OS0	Lipase, GDSL domain containing	1	gi|5379301	157	38.84091	90	17.64676	2.	1.1381	4.87E-	1.

6T0531900-01	protein.	576	8|dbj|BAD54230.1| putative lipase					201023	74	08	04E-09
OS05T0320750-00	Hypothetical gene.	601	PREDICTED: uncharacterized protein LOC100833688	282	182.945	115	59.1292	3.093988	1.629468	1.26E-24	5.67E-27

OS10T0411800-01	Similar to 40S ribosomal protein S17-3.	7336	gi|31432043|gb|AAP53735.1| 40S ribosomal protein S17-4, putative, expressed	499	264.3436	271	113.7811	2.3232264	1.216153	4.78E-28	1.87E-30
OS06T0567433-00	Hypothetical protein.	1010	no hit	37	14.28323	10	3.059548	4.66684	2.222932	4.23E-05	1.63E-0

								11			6
OS06T0498000-00	Conserved hypothetical protein.	685	gi|52076842|dbj|BAD45784.1| tyrosine specific protein phosphatase-like	21	11.95294	3	1.353347	8.832129	3.142761	0.000273	1.333E-05
OS01T0282	Hypothetical protein.	133	PREDICTED: uncharacter	136	39.71949	84	19.4436	2.04	1.030552	5.13E-06	1.61

866-00		5	ized protein LOC100947132					2805			E-07
OS03T0360200-00	Hypothetical protein.	816	no hit	46	21.97929	13	4.923022	4.464593	2.158529	4.90E-06	1.53E-07
OS11T0	Conserved hypothetical protein.	63	hypothetical protein	55	33.55891	27	13.05694	2.5	1.361879	0.000592	3.2

636600-02		9						70196			2E-05
OS04T0462900-01	Similar to prohibitin2.	1219	B1358B12.15	90	28.78623	43	10.90042	2.6640836	1.400995	1.93E-06	5.550E-08
OS1	Similar to ASCAB9-A (ASCAB9-B)	1	gi|6273386	285	93.77185	174	45.37425	2.	1.0472	1.30E-	1.

1T0<br>242<br>800-<br>01	(Fragment).	1<br>8<br>5	9|gb|AAX95<br>978.1| chlorophyll<br>a/b-binding<br>protein<br>CP26<br>precursor -<br>maize					0<br>6<br>6<br>6<br>3<br>1	81	12	5<br>2<br>E-<br>1<br>4
OS1<br>2T0<br>616<br>500-<br>00	Similar to cation proton exchanger.	1<br>9<br>2<br>3	gi|1088629<br>67|gb|ABA9<br>9860.2| cation/hydr<br>ogen	67	13.58443	26	4.178041	3.<br>2<br>5<br>1<br>3	1.7010<br>56	2.26E-<br>06	6.<br>5<br>0<br>E-<br>0

			exchanger, putative, expressed					8 8		8
OS03T0103200-01	Similar to Physical impedance induced protein.	7 3 6	gi|108705708|gb|ABF93503.1| Protease inhibitor/seed storage/LTP family protein, expressed	43	22.7791	8	3.358851	6.7818814	2.761671	2.11E-07	4.94E-0 09

OS02T0734400-00	Similar to ABA-induced protein.	786	putative caleosin	123	61.01387	64	25.16147	2.424893	1.277921	1.42E-07	3.222E-09
OS01T0502700-00	Similar to Histone H2A.	931	gi|195605364|gb|ACG24512.1| histone H2A	99	41.46021	48	15.93199	2.660232	1.379801	6.73E-07	1.776E-08

OS07T0294300-01	Conserved hypothetical protein.	490	hypothetical protein OsJ_23894	106	84.34431	54	34.05464	42.476735	1.308439	8.24E-07	2.18E-08
OS09T0485050-00	Hypothetical protein.	1604	no hit	28	6.806121	7	1.348566	5.0469	2.335406	0.000342	1.73E-0

								31			5
OS02T0585700-00	Quinonprotein alcohol dehydrogenase-like domain containing protein.	267	gi|46806267|dbj|BAD17475.1| hypothetical protein	151	220.5016	34	39.35014	5.6035578	2.486348	8.47E-23	4.17E-25
OS01T0668100-	Similar to Arabinogalactan-like protein.	1040	gi|56202178|dbj|BAD73656.1| arabinogala	293	109.845	146	43.38086	2.532	1.340339	3.95E-19	2.54E-

01			ctan protein-like					108			21
OS04T0517500-01	Similar to Phosphoenolpyruvate carboxylase kinase.	1225	gi|38568060|emb|CAE05449.3| OSJNBa0073E02.9	59	18.77854	29	7.315441	2.5666974	1.360068	0.000336	1.69E-05
OS02T0585	Hypothetical gene.	695	no hit	1532	859.4487	878	390.3807	2.220	1.13853	4.68E-78	4.32

650-01								1566			E-81
OS09T0293601-01	Conserved hypothetical protein.	652	hypothetical protein	79	47.2417	38	18.01004	2.6623076	1.39126	1.21E-05	4.09E-007
OS02T0	Similar to high mobility group family.	10	gi|113536005|dbj|BAF	124	46.89311	57	17.08421	2.7	1.456713	2.85E-09	5.0

<table>
<tr><td>258
200-
01</td><td></td><td>3
1</td><td>08388.1|
Os02g0258
200, partial</td><td></td><td></td><td></td><td></td><td>4
4
8
2
2</td><td></td><td></td><td>8
E-
1
1</td></tr>
<tr><td>OS0
6T0
115
700-
02</td><td>Hypothetical conserved gene.</td><td>9
5
5</td><td>unknown
protein</td><td>35</td><td>14.28929</td><td>10</td><td>3.235752</td><td>4.
4
1
6
0
6
5</td><td>2.1427
61</td><td>0.0001
19</td><td>5.
1
7
E-
0
6</td></tr>
<tr><td>OS0</td><td>Photosystem I reaction center subunit</td><td>6</td><td>gi|3885894|</td><td>69</td><td>39.79682</td><td>39</td><td>17.82775</td><td>2.</td><td>1.1585</td><td>0.0007</td><td>4.</td></tr>
</table>

5T0560000-01	VI, chloroplast precursor (PSI- H) (Light-harvesting complex I 11 kDa protein) (GOS5 protein).	76	gb|AAC78107.1| photosystem-1 H subunit GOS5					232296	29	28	07E-05
OS03T0670700-04	Similar to Glycine-rich protein.	746	gi|108710321|gb|ABF98116.1| retrotransposon protein, putative,	4258	2225.424	1960	811.8875	2.741054	1.454729	0	0

OS02T0175600-01	Similar to 60S ribosomal protein L27a (Fragment).	7226	Ty1-copia subclass, expressed gi|50251205|dbj|BAD27612.1| putative 60S ribosomal protein L27a	578	310.4111	311	132.3739	2.3449574	1.229561	4.40E-33	1.37E-335
OS03T0	Phospholipase A2 family protein.	12	gi|1393729 1|gb|AAK50	46	14.31373	21	5.179011	2.7	1.466651	0.000985	5.7

<table>
<tr><td>708000-01</td><td></td><td>53</td><td>122.1|AC087797_7 putative phospholipase</td><td></td><td></td><td></td><td></td><td>63796</td><td></td><td></td><td>9E-05</td></tr>
<tr><td>OS05T0472000-00</td><td>Similar to LSH6 (LIGHT SENSITIVE HYPOCOTYLS 6).</td><td>717</td><td>gi|51038215|gb|AAT94018.1| hypothetical protein</td><td>31</td><td>16.85732</td><td>9</td><td>3.878841</td><td>4.3459968</td><td>2.119678</td><td>0.000394</td><td>2.04E-05</td></tr>
<tr><td>OS0</td><td>Conserved hypothetical protein.</td><td>6</td><td>PREDICTE</td><td>361</td><td>220.2685</td><td>171</td><td>82.69397</td><td>2.</td><td>1.4134</td><td>1.42E-</td><td>6.</td></tr>
</table>

6T0 131 001- 00		3 9	D: uncharacter ized protein LOC67999 4					6 6 3 6 5 8	09	25	1 6 E- 2 8
OS0 7T0 184 800- 01	Similar to Variant of histone H1.	1 1 7 7	gi|2555362 7|dbj|BAC2 4887.1| putative histone H1	226	74.86485	122	32.03037	2. 3 3 7 3 0 8	1.2248 48	7.33E- 13	8. 3 5 E- 1 5

OS12T0166700-01	Conserved hypothetical protein.	17441	hypothetical protein OsI_15922	44	9.853713	18	3.194864	3.0844236	1.624913	0.000429	2.24E-05
OS01T0644500-00	Hypothetical conserved gene.	10558	gi|20161567|dbj|BAB90489.1| hypothetical protein	45	16.58337	12	3.504888	4.73149	2.242297	3.50E-06	1.06E-07

								8			
OS09T0481200-01	Similar to photosystem I reaction center subunit V.	668	gi|50725867|dbj|BAD33396.1| putative Photosystem I reaction center subunit V	203	118.4856	101	46.72222	2.53595958	1.342531	2.63E-13	2.77E-15
OS01T0652800-	Protein of unknown function DUF231, plant domain containing protein.	1741	gi|19571153|dbj|BAB86576.1| leaf senescence	97	21.72296	55	9.762084	2.2225	1.15396	3.29E-05	1.23E-

01			protein-like					238			06
OS08T0163500-01	Protein of unknown function DUF1005 family protein.	19928	gi|37806298|dbj|BAC99812.1| unknown protein	94	19.00933	54	8.654965	2.196335	1.135108	6.13E-05	2.45E-06
OS01T0287600-	Similar to Chitinase 10.	1055	gi|56783904|dbj|BAD81341.1| putative	76	28.08711	33	9.665851	2.905	1.53894	2.88E-06	8.51E-

01			chitinase					8 0 9		0 8
OS0 1T0 580 800- 01	Similar to submergence induced protein SI397.	2 5 2 7	gi|5379342 6|dbj|BAD5 3149.1| alpha-mann osidase-like	92	14.19478	39	4.769117	2. 9 7 6 3 9 5	1.5735 66	9.52E- 08	2. 1 1 E- 0 9
OS0 7T0 125	Allergen V5/Tpx-1 related family protein.	6 7 7	gi|2820131 5|dbj|BAC5 6823.1|	309	177.9573	128	58.42516	3. 0 4	1.6068 69	1.85E- 26	7. 6 1

201-00			putative pathogenesis-related protein					5902			E-29
OS01T0799500-01	Methyladenine glycosylase domain containing protein.	1490	gi|55297687|dbj|BAD68277.1| putative DNA-3-methyladenine glycosylase I	36	9.420245	13	2.696098	3.494029	1.804892	0.000664	3.65E-05
OS0	Conserved hypothetical protein.	1	gi|2556748	121	31.96281	52	10.88668	2.	1.5538	6.10E-	1.

3T0743650-01		476	86|dbj|BAH92367.1|Os03g0743650					935955	3	10	00E-11
OS03T0795300-01	Similar to Extensin protein-like.	14408	gi|50400028|gb|AAT76416.1|putative leucine-rich receptor-like protein	38	10.52269	14	3.072586	3.424703	1.775979	0.000512	2.73E-05

			kinase								
OS02T0186900-01	Cytochrome P450 family protein.	17009	gi|46390074|dbj|BAD15449.1| putative cytochrome P450	68	15.51361	33	5.966924	2.599934	1.378475	7.48E-05	3.07E-06
OS05T0556400-01	DOMON related domain containing protein.	14457	gi|49328007|gb|AAT58708.1| unknown protein	36	9.633607	13	2.757163	3.49940	1.804892	0.000664	3.65E-0

								29			5
OS03T0209000-01	Similar to MFP1 attachment factor 1.	940	gi|26006500|gb|AAN77309.1| Unknown protein	63	26.13116	24	7.889727	3.3120449	1.727724	3.82E-06	1.17E-07
OS05T0533500-	Similar to Serine acetyltransferase.	1333	RecName: Full=Probable serine acetyltransf	98	28.66434	51	11.82275	2.4224	1.277691	4.12E-06	1.27E-

01			erase 5; AltName: Full=OsSERAT1;2					506			07
OS01T0750100-02	Similar to WRKY13 transcription factor.	10068	hypothetical protein OsJ_03461	54	19.71372	24	6.944142	2.8388899	1.505331	0.000193	8.92E-06
OS03T0		10	gi|108707225|gb|ABF9	45	16.12611	20	5.68041	2.8	1.505331	0.00087	5.0

254400-00		88	5020.1| Patatin-like phospholipase family protein, expressed					38899			2E-05
OS11T0108800-00	Similar to Protein kinase domain containing protein, expressed.	23355	Protein kinase domain containing protein, expressed	81	13.41035	47	6.167165	2.1744476	1.120668	0.000301	1.50E-05

OS01T0582600-01	Phospholipase A2, active site domain containing protein.	21147	unknown protein	160	29.05587	83	11.94606	2.4322256	1.282295	7.73E-10	1.29E-11
OS08T0407600-01	Protein of unknown function DUF581 family protein.	11108	gi|37572957|dbj|BAC98607.1| unknown protein	62	21.81714	29	8.08792	2.69749	1.431622	0.000106	4.55E-06

OS03T0575500-01	Conserved hypothetical protein.	698	unknown protein	197	110.0416	72	31.8754	83.4552241	1.787533	1.37E-19	8.50E-22
OS05T0134400-01	Haem peroxidase, plant/fungal/bacterial family protein.	11166	gi|113578118|dbj|BAF16481.1| Os05g0134400	216	72.22727	127	33.65765	2.14459	1.101609	2.02E-10	3.12E-1

								39			2

<table>
<tr><th>#Gene</th><th>RAP-DB Description</th><th>Length</th><th>Function</th><th>read_A</th><th>RPKM_A</th><th>read_B</th><th>RPKM_B</th><th>Ration</th><th>log2(Fold_change) normalized</th><th>q-value(Benjamini et al. 1995)</th><th>p_value</th></tr>
<tr><td>OS12T0635400-01</td><td>VQ domain containing protein.</td><td>815</td><td>gi|255670515|dbj|BAH95805.1| Os12g0635400</td><td>1</td><td>0.478397</td><td>28</td><td>10.61644</td><td>0.045062</td><td>-4.47195</td><td>1.24E-05</td><td>4.22E-07</td></tr>
<tr><td>OS03T0161900-01</td><td>Similar to Isoform 2 of Heat stress transcription factor A-2d.</td><td>2271</td><td>predicted protein</td><td>31</td><td>5.322192</td><td>90</td><td>12.24627</td><td>0.434597</td><td>-1.20225</td><td>0.000342</td><td>1.73E-05</td></tr>
<tr><td>OS12T0</td><td></td><td>16</td><td>gi|113644331|dbj|BAF</td><td>60</td><td>14.32554</td><td>153</td><td>28.95235</td><td>0.4</td><td>-1.01509</td><td>2.77E-05</td><td>1.0</td></tr>
</table>

123700-00		33	27472.1|Os11g0126900					94797			3E-06
OS03T0211500-01	Hypothetical conserved gene.	14000	gi|125585366|gb|EAZ26030.1| hypothetical protein OsJ_09883	35	9.747337	121	26.70767	0.364964	-1.45417	3.25E-07	7.97E-09
OS05T0369300-01	Exo70 exocyst complex subunit domain containing protein.	1836	gi|47777390|gb|AAT38024.1| unknown protein	30	6.370809	93	15.65269	0.4070111	-1.29686	8.52E-05	3.57E-06
OS02T0743800-02	CS domain containing protein.	1518	gi|46390631|dbj|BAD16114.1| putative salt tolerance protein 5	8	2.054775	46	9.36407	0.2194322	-2.18816	6.09E-05	2.44E-06

OS04T0651000-01	Similar to Peroxidase.	1364	gi|38345909|emb|CAE04507.2|OSJNBb0059K02.17	23	6.57445	92	20.84261	0.315433	-1.66459	1.04E-06	2.82E-08
OS08T0500800-01	WLM domain containing protein.	2573	gi|42407752|dbj|BAD08898.1|zinc metalloproteinase-like	97	14.69867	445	53.44398	0.275029	-1.86234	3.20E-37	8.57E-40
OS06T0548000-01	Aspartate aminotransferase (EC 2.6.1.1).	1670	gi|113595865|dbj|BAF19739.1|Os06g0548000	108	25.21467	286	52.92102	0.476459	-1.06958	2.01E-10	3.09E-12
OS11T0406945-00	Similar to Phenazine biosynthesis-like protein, expressed.	715	gi|255674616|dbj|BAF12347.2|Os03g0434400	13	7.088973	60	25.93127	0.27333	-1.87104	3.10E-05	1.16E-0

								75			6
OS02T0139000-01	Transcription factor, Regulation of Pi signaling and homeostasis, Tolerance to low-Pi stress	1856	gi|42409277|dbj|BAD10540.1| putative transfactor	137	28.77985	487	81.08296	0.3549443	-1.49434	9.37E-30	3.40E-32
OS02T0261100-01	Ubiquitin-conjugating enzyme E2, Elicitor-induced defense response	727	gi|20152205|dbj|BAB89355.1| ubiquitin-conjugating enzyme OsUBC5b	199	106.7246	555	235.905	0.452405	-1.14431	2.11E-22	1.07E-24
OS08T0500700-01	Similar to Heat shock protein 82.	3146	gi|113624128|dbj|BAF24073.1| Os08g0500700, partial	1295	160.4933	7073	694.742	0.231011	-2.11396	0	0
OS07T0182	Basic leucine zipper transcriptional activator, Grain filling	178	gi|13365770|dbj|BAB39173.1|	24	5.248146	116	20.10413	0.26	-1.93761	2.83E-10	4.46

000-01		3	RISBZ1					1048			E-12
OS10T0177200-02	EF-HAND 2 domain containing protein.	1643	gi|78707963|gb|ABB46938.1| 39 kDa EF-Hand containing protein, putative, expressed	39	9.254928	368	69.21319	0.133716	-2.90275	2.80E-52	4.92E-55
OS03T0284150-00	Hypothetical protein.	2471	no hit	37	5.838146	104	13.00586	0.448886	-1.15558	0.000168	7.61E-06
OS06T0538400-01	Conserved hypothetical protein.	920	gi|53793118|dbj|BAD54327.1| unknown protein	106	44.92251	302	101.4373	0.44286	-1.17508	9.31E-13	1.07E-14

OS06T0114500-01	Similar to ATOZI1 protein (Stress-induced protein OZI1) (AT0ZI1 protein).	637	gi|7248405|dbj|BAA92728.1| ozone-responsive stress-related protein-like	41	25.09519	109	52.87686	0.4745997	-1.07523	0.000308	1.54E-05
OS12T0111800-00	Similar to Class III peroxidase 136.	1142	TPA: class III peroxidase 136 precursor	13	4.438367	56	15.15307	0.2929022	-1.77151	0.000137	6.03E-06
OS11T0502700-01	Glycosyl transferase, family 31 domain containing protein.	968	expressed protein	46	18.528	169	53.94981	0.343433	-1.54191	7.44E-11	1.08E-12
OS08T0296900-01	Tetratricopeptide-like helical domain containing protein.	3288	gi|50508716|dbj|BAD31284.1| putative octicosapep	97	11.50233	249	23.40163	0.49915	-1.02468	1.69E-08	3.34E-1

			tide/Phox/Bem1p (PB1) domain-/tetratricopeptide repeat (TPR)-containing protein					18			0
OS01T0624500-01	Similar to Sgt1.	582	gi|113533151|dbj|BAF05534.1| Os01g0624500	402	269.3079	1171	621.7453	0.43331 48	-1.20707	8.80E-52	1.57E-54
OS03T0266300-03	Class I low-molecular-weight heat shock protein 17.9.	851	gi|18031727|gb|AAK54445.1| class I low-molecular-weight heat shock protein 17.9	53	24.28244	338	122.7342	0.19784 6	-2.33755	8.88E-38	2.32E-40
OS01T0524	Armadillo-like helical domain containing protein.	354	gi|56202358|dbj|BAD73838.1|	45	4.954874	118	10.29757	0.48	-1.05538	0.000202	9.40

700-01		1	unknown protein					1169			E-06
OS02T0150900-02	Protein of unknown function DUF1644 family protein.	1478	gi|113535436|dbj|BAF07819.1|Os02g0150900	34	8.969133	93	19.44407	0.461279	-1.11629	0.000693	3.84E-05
OS06T0129900-02	Similar to cytochrome P450.	1751	gi|52075627|dbj|BAD44798.1|putative cytochrome P450	60	13.36014	197	34.76632	0.3842884	-1.37975	1.01E-10	1.49E-12
OS07T0661600-01	Similar to RNA-binding protein-like protein.	1717	gi|38175742|dbj|BAC84316.2|zinc finger protein family-like	37	8.401898	173	31.1354	0.269985	-1.88977	9.92E-15	9.34E-17
OS04T0	Similar to Syn-copalyl diphosphate synthase.	27	gi|113563742|dbj|BAF	9	1.267717	48	5.35863	0.2	-2.07963	7.58E-05	3.1

178300-02		68	14085.1|Os04g0178300					36575			2E-06
OS08T0433350-00	Similar to photosystem II protein I.	254	photosystem II protein I, partial	34	52.19047	108	131.3919	0.3972112	-1.33202	1.03E-05	3.41E-07
OS02T0209300-01	Hypothetical conserved gene.	566	hypothetical protein OsJ_05853	14	9.644009	60	32.7577	0.2944404	-1.76413	7.33E-05	3.00E-06
OS06T0143700-01	Similar to Sulfate transporter 2.	2494	gi|55296351|dbj|BAD68396.1|putative sulfate transporter Sultr3;4	148	23.13722	430	53.27833	0.4342271	-1.20333	8.23E-19	5.47E-21

OS01T0667700-04	Zinc finger, RING/FYVE/PHD-type domain containing protein.	2234	gi|56202174|dbj|BAD73652.1| C-terminal zinc-finger-like	36	6.282975	121	16.73712	0.37539 2	-1.41353	6.25E-07	1.63E-08
OS03T0748700-01	Iron hydrogenase domain containing protein.	1801	gi|18087682|gb|AAL58974.1|AC091811_23 putative hydrogenase	211	45.6788	595	102.0897	0.447438	-1.16024	1.44E-24	6.53E-27
OS11T0453900-01	Dehydrin RAB 16D.	852	gi|62734023|gb|AAX96132.1| dehydrin rab 16d	2	0.915243	122	44.24853	0.020684	-5.59533	3.45E-25	1.51E-27
OS08T0331000-01	Conserved hypothetical protein.	460	gi|255678363|dbj|BAF23477.2| Os08g0331000, partial	171	144.9387	457	306.999	0.47721	-1.08279	6.83E-17	5.40E-1

								14			9
OS02T0139100-01	Activator of Hsp90 ATPase, N-terminal domain containing protein.	1087	gi|42409278|dbj|BAD10541.1| unknown protein	106	38.02089	555	157.7764	0.240098	-2.05302	6.39E-53	1.02E-55
OS02T0788800-02	Similar to amino acid transporter family protein.	1212	hypothetical protein OsJ_08666	21	6.75558	99	25.24127	0.267764	-1.90163	1.38E-08	2.70E-10
OS07T0150700-01	Serine/threonine protein kinase, Pollination and drought stress responses, Reguration of potassium uptake by CBL1-CIPK23 complex	1891	gi|34393400|dbj|BAC82911.1| putative CBL-interacting protein kinase 23	804	165.7717	2079	339.736	0.4879443	-1.03522	6.62E-72	6.93E-75
OS06T0701700-02	Similar to Cation transporter HKT1.	768	sodium transporter	16	8.122781	116	46.67404	0.17740	-2.52257	3.95E-14	3.87E-1

								32			6
OS01T0933500-00	Similar to FLP1.	442	gi|21104655|dbj|BAB93246.1| FPF1 protein-like	17	14.9959	73	51.0363	0.293828	-1.76696	7.67E-06	2.50E-07
OS08T0335600-01	Protein of unknown function DUF568, DOMON-like domain containing protein.	925	gi|38424013|dbj|BAD01770.1| auxin-induced protein-like	43	18.12478	120	40.08834	0.452121	-1.14522	4.64E-05	1.81E-06
OS06T0154200-02	Similar to F-box/LRR-repeat MAX2 homolog.	1803	gi|55297437|dbj|BAD69288.1| F-box protein ORE9-like	59	12.75858	165	28.27918	0.451165	-1.14827	7.72E-07	2.03E-08
OS01T0239	Heat shock protein DnaJ family protein.	172	gi|11034711|dbj|BAB17212.1|	36	8.122781	114	20.38636	0.39	-1.32756	5.51E-06	1.74

100-01		8	putative DnaJ-like protein					8442			E-07
OS01T0316600-01	Similar to pre-mRNA-splicing factor SF2.	2194	hypothetical protein OsI_01613	83	14.74984	248	34.9296	0.42222273	-1.24375	1.74E-11	2.36E-13
OS06T0668250-00	Hypothetical gene.	1172	no hit	10	3.326736	45	11.86488	0.28038 5	-1.83452	0.000597	3.25E-05
OS10T0569400-01	RIR1a protein precursor.	631	gi|2584803|emb|CAA75103.1|RIR1a protein	1253	774.2259	3818	1869.757	0.4140788	-1.27202	5.51E-185	2.71E-188
OS0	Hypothetical conserved gene.	1	gi|4025329	121	32.24683	309	65.26687	0.	-1.0171	2.23E-	3.

8T0175200-02		463	0|dbj|BAD05225.1| unknown protein					494077	9	10	49E-12
OS03T0724700-03	Maf-like protein family protein.	17778	gi|108710827|gb|ABF98622.1| Maf-like protein, expressed	123	26.97238	402	69.86713	0.38605 3	-1.37313	1.39E-21	7.49E-24
OS03T0326000-01	Phospholipid-transporting ATPase 1 (EC 3.6.3.1) (Aminophospholipid flippase 1).	880	hypothetical protein OsJ_10682	65	28.79895	193	67.77246	0.4249 36	-1.23468	7.00E-09	1.31E-10
OS01T0719100-01	Similar to RING finger and CHY zinc finger domain-containing protein 1.	2344	gi|57899891|dbj|BAD87761.1| zinc finger protein ZFP-like	165	27.44557	578	76.19892	0.36018	-1.4732	1.32E-34	3.90E-37

OS09T0440851-01	Hypothetical gene.	2991	no hit	73	9.515956	196	20.24968	30.469931	-1.08948	1.99E-07	4.63E-09
OS06T0682900-01	Heat stress associated protein, Long-term acquired thermotolerance	1283	gi|52076646|dbj|BAD45546.1| (2R)-phospho-3-sulfolactate synthase-like	59	17.92963	207	49.85656	0.3596624	-1.47544	2.02E-12	2.38E-14
OS05T0405000-02	Similar to Isoform 2 of Pyruvate, phosphate dikinase 1, chloroplastic.	2966	gi|50878307|gb|AAT85082.1| putative pyruvate orthophosphate dikinase	154	20.24396	390	40.63236	0.498223	-1.00514	8.75E-13	1.00E-14
OS03T0	Class I low-molecular-weight heat shock protein 17.9.	99	gi|18031727|gb|AAK54	47	18.5101	340	106.1261	0.1	-2.5194	1.67E-41	4.1

266300-01		0	445.1| class I low-molecular-weight heat shock protein 17.9					74416			2E-44
OS12T0516200-00	Conserved hypothetical protein.	423	gi|77555915|gb|ABA98711.1| expressed protein	65	59.91271	408	298.0564	0.2010111	-2.31465	3.81E-45	8.21E-48
OS01T0184100-01	Similar to 17.5 kDa class II heat shock protein.	807	gi|55295926|dbj|BAD67794.1| putative 18kDa heat shock protein	19	9.179649	278	106.451	0.08862334	-3.53561	5.18E-47	1.04E-49
OS11T0697600-00	Hypothetical gene.	816	legume+lectins+beta+domain+containing+protein	30	14.33432	90	34.08246	0.420557	-1.24956	0.000203	9.44E-06

								8			
OS02T0226300-00	Mitochondrial carrier protein domain containing protein.	1074	hypothetical protein OsI_06431	19	6.897557	81	23.30555	0.2959962	-1.75652	2.18E-06	6.28E-08
OS05T0456800-01	Hypothetical gene.	2467	no hit	25	3.951089	119	14.90584	0.26507	-1.91556	2.22E-10	3.47E-12
OS01T0642200-01	Conserved hypothetical protein.	458	gi|55297100|dbj|BAD68744.1| unknown protein	916	779.787	3648	2461.319	0.316817	-1.65828	3.66E-263	1.58E-266
OS03T0267000-00	Low molecular mass heat shock protein Oshsp18.0.	708	gi|29893629|gb|AAP06883.1| unknown protein	0.5	0.275349	50	21.82305	0.0126	-6.30845	2.54E-10	4.00E-1

								17			2
OS10T0492900-01	Similar to alpha-galactosidase.	979	gi|31432821|gb|AAP54408.1| Alpha-galactosidase precursor, putative, expressed	9	3.584312	95	29.98607	0.1195333	-3.06452	3.75E-14	3.65E-16
OS01T0734800-02	UDP-glucuronosyl/UDP-glucosyltransferase family protein.	1664	gi|15624028|dbj|BAB68082.1| putative glucosyltransferase	3	0.702933	29	5.385466	0.1305524	-2.93761	0.000218	1.02E-05
OS02T0543000-03	Similar to Abscisic stress ripening protein 1.	474	unknown protein	120	98.70721	331	215.7885	0.4574426	-1.12839	4.33E-13	4.71E-15
OS09T0	Protein of unknown function DUF125, transmembrane family protein.	93	gi|50252604|dbj|BAD2	221	92.15664	628	207.5519	0.4	-1.17131	2.42E-26	1.0

396900-01		5	8775.1| integral membrane-like protein					440170		1E-28
OS10T0524400-01	Similar to Phospholipase D beta 2.	3898	gi|27311294|gb|AAO00720.1| putative phospholipase	46	4.601103	120	9.513011	0.4836664	-1.04792	0.000193	8.91E-06
OS05T0542500-01	Similar to Isoform 2 of Late embryogenesis abundant protein, group 3.	1276	gi|1486503|gb|AAC03364.1| LEA-like protein	3	0.916677	41	9.929143	0.09232322	-3.43718	6.90E-07	1.81E-08
OS08T0530033-00	Hypothetical protein.	1016	no hit	23	8.826329	73	22.2028	0.39753532	-1.33086	0.000522	2.79E-05

OS06T0674100-01	Conserved hypothetical protein.	806	gi|255677315|dbj|BAF20256.2| Os06g0674100, partial	16	7.739821	57	21.85337	0.3541771	-1.49748	0.000897	5.19E-05
OS10T0569800-00	Similar to RIR1b protein.	564	expressed protein	108	74.66046	315	172.5878	0.4325994	-1.20891	6.75E-14	6.77E-16
OS07T0418600-01	Proline-rich glycoprotein, ABA-dependent inhibition of root growth	1057	gi|22830951|dbj|BAC15815.1| unknown protein	660	243.4529	2258	660.1271	0.3687797	-1.4391	7.33E-132	4.74E-135
OS08T0412700-01	Protein of unknown function DUF1262 family protein.	1491	gi|37806193|dbj|BAC99696.1| unknown protein	21	5.491458	67	13.88596	0.39954	-1.33837	0.000951	5.55E-0

								68			5
OS06T0297800-01	Similar to protein binding / zinc ion binding.	4207	gi|53791982|dbj|BAD54435.1| putative Osnop	54	5.004575	140	10.28334	0.486668	-1.03899	4.81E-05	1.89E-06
OS09T0400600-01	Hypothetical protein.	1505	no hit	14	3.626916	56	11.49821	0.315433	-1.66459	0.000298	1.48E-05
OS02T0773700-01	Hypothetical gene.	6455	no hit	52	3.140893	143	6.845708	0.458812	-1.12403	8.68E-06	2.85E-07
OS05T0142	Conserved hypothetical protein.	615	gi|46485810|gb|AAS98435.1|	113	71.63897	315	158.2756	0.45	-1.14362	9.64E-13	1.12

100-01			unknown protein					2622			E-14
OS03T0293000-01	Similar to DnaJ domain containing protein, expressed.	1817	hypothetical protein OsJ_10460	59	12.66027	177	30.10211	0.420578	-1.24956	2.56E-08	5.23E-10
OS11T0609600-01	Similar to 14-3-3-like protein.	2438	gi|77551936|gb|ABA94733.1| 14-3-3, putative, expressed	14	2.238929	55	6.971201	0.321168	-1.6386	0.000419	2.19E-05
OS01T0918400-00	Conserved hypothetical protein.	890	hypothetical protein OsJ_04556	7	3.066578	38	13.19387	0.232424	-2.10517	0.000539	2.90E-05
OS0	Similar to Branched chain alpha-keto	1	gi|5050973	48	14.29709	135	31.86931	0.	-1.1564	1.01E-	3.

7T0170100-02	acid dehydrogenase E1 beta subunit.	309	9|dbj|BAD31791.1| putative branched-chain alpha-keto acid decarboxylase E1 beta subunit					448616	5	05	35E-07
OS12T0594950-01	Hypothetical conserved gene.	751	gi|77556385|gb|ABA99181.1| expressed protein	2	1.038332	65	26.74558	0.038823	-4.68696	6.33E-13	7.08E-15
OS03T0381300-01	CS domain domain containing protein.	1138	gi|40882701|gb|AAR96242.1| putative ripening regulated protein	161	55.16068	462	125.4522	0.439695	-1.18543	9.99E-20	6.09E-22
OS04T0	Similar to Suppressor of presenilin 5 (P110b homolog).	20	gi|32488409|emb|CAE	65	12.29053	203	30.42188	0.4	-1.30756	3.46E-10	5.5

671<br>200-<br>01		6<br>2	02834.1| OSJNBa00<br>43A12.39					0<br>4<br>0<br>0<br>3			0<br>E-<br>1<br>2
OS0<br>3T0<br>724<br>700-<br>02	Similar to cDNA clone:002-144-A12, full insert sequence.	1<br>3<br>1<br>3	gi|2157412<br>37|dbj|BAG<br>97732.1| unnamed<br>protein<br>product	110	32.66434	349	82.13709	0.<br>3<br>9<br>7<br>6<br>8<br>1	-1.3303<br>2	7.47E-<br>18	5.<br>2<br>9<br>E-<br>2<br>0
OS0<br>4T0<br>533<br>900-<br>01	Heavy metal transport/detoxification protein domain containing protein.	2<br>9<br>3<br>4	gi|1255911<br>01|gb|EAZ3<br>1451.1| hypothetical<br>protein<br>OsJ_15587	27	3.587977	91	9.584289	0.<br>3<br>7<br>4<br>3<br>6	-1.4175	2.50E-<br>05	9.<br>2<br>0<br>E-<br>0<br>7
OS0<br>1T0<br>719<br>100-<br>02	Similar to PGPD14 protein.	1<br>6<br>9<br>2	gi|5789989<br>1|dbj|BAD8<br>7761.1| zinc finger<br>protein<br>ZFP-like	121	27.88245	390	71.2267	0.<br>3<br>9<br>1<br>4<br>6<br>1	-1.3530<br>6	1.82E-<br>20	1.<br>0<br>4<br>E-<br>2<br>2
OS0	Similar to ICT protein (Fragment).	6	unknown	467	292.7335	1333	662.2445	0.	-1.1777	1.11E-	1.

5T0138300-03		22	protein					442032	8	56	57E-59
OS07T0186000-02	H-type thioredoxin, Regulation of the apoplastic reactive oxygen species, Stress response	730	gi|426442|dbj|BAA04864.1| thioredoxin h	618	330.0742	2176	921.1167	0.3583341	-1.48059	1.52E-132	8.87E-136
OS08T0143700-01	Protein of unknown function DUF676, hydrolase-like domain containing protein.	1486	gi|46805616|dbj|BAD17029.1| unknown protein	93	24.40114	256	53.23531	0.4583364	-1.12543	3.84E-10	6.19E-12
OS11T0622800-00	Putative cinnamyl alcohol dehydrogenase 4.	1065	gi|218186132|gb|EEC68559.1| hypothetical protein OsI_36879	34	12.4473	94	27.2745	0.456637	-1.13172	0.000532	2.85E-05

OS01T0159800-01	Similar to DNA binding protein.	1473	gi|9757687|dbj|BAB08206.1| unnamed protein product	30	7.940804	87	18.25135	10.4350 8	-1.20065	0.000463	2.44E-05
OS02T0281200-01	Similar to NBS-LRR protein (Fragment).	4146	gi|47848558|dbj|BAD22409.1| putative NBS-LRR type disease resistance protein RPG1-B	42	3.949717	117	8.720375	0.452 93	-1.14264	6.31E-05	2.54E-06
OS07T0633200-01	Similar to SC35-like splicing factor SCL30a, 30a kD.	973	hypothetical protein OsJ_25252	19	7.613542	65	20.6433	0.368 814	-1.43903	0.000511	2.72E-05
OS01T0	Protein of unknown function DUF1666 family protein.	22	gi|9558467|dbj|BAB033	38	6.532607	103	14.03372	0.4	-1.10317	0.00035	1.7

129500-02		68	88.1|unknown protein					65494			8E-05
OS05T0596200-03	Similar to cDNA clone:001-104-D04, full insert sequence.	934	gi|57900670|gb|AAW57795.1|unknown protein	48	20.03735	132	43.67226	0.45881 2	-1.12403	2.23E-05	8.15E-07
OS02T0761600-01	Conserved hypothetical protein.	1614	gi|46805892|dbj|BAD17205.1|unknown protein	59	14.25261	161	30.82485	0.4623 74	-1.11287	2.27E-06	6.55E-08
OS04T0671300-01	Similar to Suppressor of presenilin 5 (P110b homolog).	2112	gi|90265249|emb|CAH67702.1|H0624F09.10	709	130.8875	2560	374.5628	0.3494 41	-1.51688	7.43E-162	3.89E-165

OS08T0105400-00	Similar to P450.	16055	gi|42407790|dbj|BAD08935.1| putative P450	24	5.830183	163	31.38276	0.1857777	-2.42836	5.43E-19	3.56E-21
OS01T0840100-01	Heat shock protein Hsp70 family protein.	22557	gi|15623835|dbj|BAB67894.1| putative HSP70	260	44.91462	1066	145.9501	0.307774	-1.70022	5.54E-79	4.78E-82
OS03T0820500-01	Similar to WCOR719.	744	gi|29124120|gb|AAO65861.1| putative actin-binding protein	7	3.668353	105	43.61089	0.0841116	-3.57148	8.36E-18	5.95E-20
OS12T0488900-01	Armadillo-like helical domain containing protein.	2603	gi|108862690|gb|ABA98278.2| Pumilio-family RNA binding	13	1.947221	54	6.410593	0.303375	-1.71904	0.000276	1.35E-05

			repeat containing protein, expressed								
OS01T0719100-03	Hypothetical gene.	952	no hit	50	20.4776	183	59.40086	0.34447336	-1.53644	1.11E-11	1.47E-13
OS02T0740500-01	Conserved hypothetical protein.	722	hypothetical protein OsI_08879	79	42.66148	203	86.88353	0.49101019	-1.02615	5.30E-07	1.35E-08
OS01T0120400-02	Alanyl-tRNA synthetase, class IIc family protein.	1182	gi|53791243|dbj|BAD52448.1| unknown protein	35	11.54507	105	27.45051	0.42057578	-1.24956	4.41E-05	1.71E-06
OS0	Similar to Low-temperature induced	4	gi|2747606	36	28.881	127	80.75066	0.	-1.4833	9.02E-	1.

3T0286900-01	protein lt101.2.	86	0|gb|AAO16991.1| Putative low-temperature induced protein					3576657	5	08	99E-09
OS08T0525600-01	Similar to Peptidylprolyl isomerase; FK506-binding protein.	2099	gi|42761405|dbj|BAD11570.1| putative 70 kDa peptidylprolyl isomerase	716	132.9984	2275	334.925	0.39700 99	-1.33243	4.47E-118	3.03E-121
OS06T0493100-01	Conserved hypothetical protein.	529	gi|52076427|dbj|BAD45256.1| brown planthopper-inducible protein-like	368	271.2303	1208	705.6508	0.38843 69	-1.37944	8.57E-66	1.03E-68
OS04T0191500-	Conserved hypothetical protein.	619	OSJNBa0059D20.12	158	99.52047	434	216.6595	0.459	-1.12236	5.31E-17	4.17E-

01								341			19
OS02T0758000-01	Similar to Low molecular weight heat shock protein precursor (Mitochondrial small heat shock protein 22).	1110	gi|46805691|dbj|BAD17092.1| putative low molecular weight heat shock protein	45	15.80649	335	93.26108	0.16694486	-2.56076	1.23E-41	2.95E-44
OS03T0113750-00	Hypothetical protein.	1970	gi|68485928|ref|XP_713152.1| hypothetical protein CaO19.1895	43	8.510366	124	19.45065	0.43753366	-1.19253	1.54E-05	5.40E-07
OS10T0419300-00	Similar to Heat shock transcription factor 31 (Fragment).	1336	gi|31432122|gb|AAP53792.1| HSF-type DNA-binding domain containing protein,	43	12.54897	133	30.76265	0.40797929	-1.29361	1.15E-06	3.16E-08

			expressed								
OS09T0563700-01	Kelch related domain containing protein.	2653	gi|52076115|dbj|BAD46628.1| unknown protein	30	4.408897	95	11.06534	0.3984442	-1.32756	4.69E-05	1.84E-06
OS01T0348900-01	SalT gene product (Salt-induced protein).	684	gi|15076931|gb|AAK82986.1|AF285163_1 salt-induced protein	432	246.2485	1305	589.5668	0.4176777	-1.25954	9.68E-62	1.25E-64
OS11T0255500-01	Similar to Allyl alcohol dehydrogenase; NADP-dependent oxidoreductase-like protein.	1278	gi|62732671|gb|AAX94790.1| oxidoreductase, zinc-binding dehydrogenase family	34	10.37275	114	27.56466	0.3763006	-1.41002	1.60E-06	4.46E-08
OS09T0412	Conserved hypothetical protein.	590	gi|50251466|dbj|BAD28531.1|	52	34.36349	153	80.13422	0.42	-1.22154	5.46E-07	1.40

700-01			unknown protein					88824			E-08
OS07T0452500-02	ORMDL family protein.	940	gi|34394897|dbj|BAC84413.1| putative ORMDL2	88	36.50067	226	74.29493	0.4912924	-1.02534	9.57E-08	2.12E-09
OS08T0130100-01	Zinc finger, LSD1-type domain containing protein.	863	gi|29467531|dbj|BAC66720.1| putative zinc-finger protein Lsd1	112	50.60031	353	126.3987	0.4003233	-1.32076	7.41E-18	5.22E-20
OS10T0506100-01	Heavy metal transport/detoxification protein domain containing protein.	641	gi|31432950|gb|AAP54521.1| heavy-metal-associated domain-containing protein,	915	556.5562	2882	1389.359	0.400585	-1.31982	1.26E-147	6.98E-151

			putative, expressed								
OS12T0478200-01	Hypothetical protein.	456	no hit	6	5.130178	60	40.65978	0.1261 73	-2.98652	8.39E-09	1.58E-10
OS05T0405000-01	Orthophosphate dikinase precursor (EC 2.7.9.1).	3238	gi|50878307|gb|AAT85082.1| putative pyruvate orthophosphate dikinase	152	18.3026	405	38.65065	0.473539	-1.07844	7.19E-15	6.58E-17
OS07T0418700-01	Proline-rich glycoprotein, ABA-dependent inhibition of root growth	1088	gi|22830955|dbj|BAC15819.1| unknown protein	320	114.6746	1168	331.736	0.345668	-1.53249	1.65E-74	1.62E-77
OS11T0197	Transcriptional factor B3 family protein.	188	gi|108864092|gb|ABA91888.2| B3	42	8.696509	121	19.857	0.43	-1.19114	2.09E-05	7.58

<table>
<tr><td>600-01</td><td></td><td>3</td><td>DNA binding domain containing protein, expressed</td><td></td><td></td><td></td><td></td><td>7957</td><td></td><td></td><td>E-07</td></tr>
<tr><td>OS05T0182900-01</td><td>Conserved hypothetical protein.</td><td>1555</td><td>gi|222630435|gb|EEE62567.1| hypothetical protein OsJ_17366</td><td>13</td><td>3.25956</td><td>55</td><td>10.92977</td><td>0.2982228</td><td>-1.74551</td><td>0.000195</td><td>9.04E-06</td></tr>
<tr><td>OS03T0277300-01</td><td>Heat shock protein 70.</td><td>2485</td><td>gi|125585785|gb|EAZ26449.1| hypothetical protein OsJ_10337</td><td>11</td><td>1.725887</td><td>46</td><td>5.720185</td><td>0.3017719</td><td>-1.72872</td><td>0.000947</td><td>5.53E-05</td></tr>
<tr><td>OS03T0745000-02</td><td>Similar to Heat stress transcription factor A-2a.</td><td>2217</td><td>gi|30017583|gb|AAP13005.1| putative heat shock factor</td><td>7</td><td>1.231057</td><td>261</td><td>36.37922</td><td>0.033384</td><td>-4.88514</td><td>8.17E-53</td><td>1.36E-55</td></tr>
</table>

OS04T0671500-00	Hypothetical protein.	2021	no hit	316	60.96306	1136	173.6963	0.35097 5	-1.51056	6.25E-71	6.733E-74
OS04T0638700-01	Conserved hypothetical protein.	794	gi|113565599|dbj|BAF15942.1| Os04g0638700	46	22.58829	127	49.42672	0.457006	-1.12972	3.18E-05	1.19E-06
OS03T0776900-01	Similar to mitochondrial import inner membrane translocase subunit TIM14.	689	gi|108711345|gb|ABF99140.1| DNAJ protein, putative, expressed	22	12.44943	81	36.32824	0.342693	-1.54501	2.18E-05	7.95E-07
OS02T0788800-01	Amino acid transporter, transmembrane domain containing protein.	2140	gi|47497045|dbj|BAD19097.1| amino acid transporter-	36	6.558956	96	13.86232	0.47731	-1.07963	0.000822	4.68E-0

			like					5			5
OS02T0731200-01	Transcription factor MADS57.	1384	gi|30313689|gb|AAO47712.1| transcription factor MADS57	13	3.662294	91	20.31814	0.18024 8	-2.47195	6.99E-11	1.01E-12
OS08T0485400-02	Similar to 2-nitropropane dioxygenase-like protein.	1970	gi|113624056|dbj|BAF24001.1| Os08g0485400	50	9.895774	172	26.97993	0.3667 83	-1.447	4.44E-10	7.24E-12
OS01T0719300-01	Similar to Sulfate transporter 3.1 (AST12) (AtST1).	2806	gi|15624010|dbj|BAB68064.1| putative plasma membrane sulphate transporter	82	11.39389	426	46.91379	0.24286 9	-2.04175	2.78E-40	6.93E-43
OS01T0719	RING zinc-finger protein, Stomata opening	184	gi|57899891|dbj|BAD87761.1|	161	34.09715	461	77.37947	0.44	-1.1823	1.34E-19	8.20

100-04		1	zinc finger protein ZFP-like					0649			E-22
OS06T0115300-01	Acyl-CoA-binding protein , Stress response	522	gi|55295897|dbj|BAD67765.1| putative Acyl-CoA-binding protein	46	34.35843	120	71.03777	0.483664	-1.04792	0.000193	8.91E-06
OS12T0183100-01	Similar to Branched chain alpha-keto acid dehydrogenase E1-alpha subunit (Fragment).	1696	gi|77553175|gb|ABA95971.1| 2-oxoisovalerate dehydrogenase alpha subunit, mitochondrial precursor, putative, expressed	23	5.287471	76	13.84734	0.381884	-1.38896	0.000214	1.00E-05
OS04T0	Similar to Glutamate dehydrogenase 2.	18	gi|125549210|gb|EAY9	145	30.00773	428	70.20071	0.4	-1.22615	2.77E-19	1.7

543900-02		84	5032.1| hypothetical protein OsI_16842					27456			7E-21
OS04T0508500-01	Similar to OSIGBa0101P20.9 protein.	1372	gi|125548977|gb|EAY94799.1| hypothetical protein OsI_16582	7	1.989253	57	12.83806	0.154495	-2.69013	1.47E-07	3.36E-09
OS11T0116300-01	Chalcone isomerase domain containing protein.	890	gi|108863935|gb|ABA91231.2| Chalcone-flavanone isomerase family protein, expressed	20	8.761652	77	26.73495	0.327723	-1.60945	1.98E-05	7.11E-07
OS11T0112200-01	Similar to Cationic peroxidase 1 precursor (EC 1.11.1.7) (PNPC1).	1140	gi|55701129|tpe|CAH69373.1| TPA: class III peroxidase	15	5.130178	82	22.22735	0.230880	-2.11526	3.47E-08	7.25E-10

			131 precursor					5			
OS03T0360000-00	Similar to Arginine decarboxylase.	981	no hit	3	1.192335	33	10.39498	0.1147033	-3.12403	3.34E-05	1.26E-06
OS09T0362800-01	Hypothetical conserved gene.	2424	gi|113631221|dbj|BAF24902.1| Os09g0362800, partial	215	34.58214	611	77.89099	0.4439981	-1.17143	1.26E-25	5.43E-28
OS06T0268800-01	Uncharacterised protein family UPF0005 domain containing protein.	1993	gi|53791768|dbj|BAD53533.1| aluminum-activated malate transporter-like	33	6.455838	105	16.28023	0.3965545	-1.33445	1.39E-05	4.78E-07
OS08T0	Similar to Indole-3-glycerol phosphate synthase-like.	15	gi|42409162|dbj|BAD1	56	13.69764	146	28.3037	0.4	-1.04706	2.65E-05	9.7

320400-01		94	0429.1| putative indole-3-glycerol phosphate synthase					83952			5E-07
OS08T0464000-01	Activator of Hsp90 ATPase homologue 1-like family protein.	1462	gi|42409065|dbj|BAD10317.1| putative activator of 90 kDa heat shock protein ATPase homolog 1	73	19.46801	190	40.15918	0.48477 1	-1.04462	9.20E-07	2.47E-08
OS04T0352400-01	Peptidyl-prolyl cis-trans isomerase, FKBP-type domain containing protein.	21161	gi|66153725|gb|AAD29708.2|AF140495_1 FK506-binding protein	42	7.577754	2143	306.4404	0.02472 28	-5.33769	0	0
OS09T0464	Similar to Carbonate dehydratase-like protein.	174	gi|50725202|dbj|BAD33953.1|	65	14.49833	183	32.35104	0.44	-1.15793	1.21E-07	2.73

000-01		8	putative carbonic anhydrase					8156			E-09
OS03T0316200-01	Similar to Galactinol synthase (Fragment).	1402	gi|108707826|gb|ABF95621.1| galactinol synthase 3, putative, expressed	4	1.112392	41	9.036795	0.1230996	-3.02215	3.46E-06	1.05E-07
OS05T0462301-00	Similar to predicted protein.	647	hypothetical protein OsJ_18826	67	40.37537	235	112.2386	0.3597288	-1.47502	4.48E-14	4.44E-16
OS08T0270900-01	Conserved hypothetical protein.	949	gi|37806366|dbj|BAC99878.1| unknown protein	40	16.43387	158	51.44812	0.3194266	-1.64645	3.71E-11	5.22E-13
OS0	Allergen V5/Tpx-1 related family	1	gi|1135800	56	16.20938	171	39.22899	0.	-1.2750	2.69E-	5.

5T0595200-01	protein.	347	64|dbj|BAF18427.1|Os05g0595200					413199	9	08	53E-10
OS01T0184050-00	Hypothetical protein.	751	gi|223974127|gb|ACN31251.1|unknown	8	4.153326	214	88.05468	0.04471668	-4.40606	1.53E-41	3.72E-44
OS12T0478200-02	GRAM domain containing protein.	623	ABA-responsive protein, putative, expressed	8	5.006658	58	28.76859	0.17740032	-2.52257	3.44E-07	8.53E-09
OS12T0448900-01	Fatty acid alpha-dioxygenase family, Enzyme that oxygenates fatty acids into 2R-hydroperoxides	2252	gi|133761837|gb|AAF64042.2|AF229813_1 fatty acid alpha-oxida	60	10.38793	171	23.46423	0.442271	-1.17556	2.49E-07	5.94E-09

			se					3			
OS06T0506600-01	Similar to Ubiquitin carrier protein.	2140	gi|355508240|gb|AES89382.1| Ubiquitin carrier protein	137	24.96047	515	74.3656	0.33356645	-1.57499	5.10E-34	1.55E-36
OS11T0428800-01	Cupredoxin domain containing protein.	762	gi|62734521|gb|AAX96630.1| basic blue copper protein	2246	1149.214	9563	3878.089	0.29633 35	-1.7547	0	0
OS07T0245100-01	Similar to Cytosine deaminase (EC 3.5.4.1).	2119	gi|113610817|dbj|BAF21195.1| Os07g0245100, partial	41	7.543951	131	19.10376	0.3948933	-1.34046	6.39E-07	1.67E-08
OS01T0283300-	Conserved hypothetical protein.	1105	gi|13486722|dbj|BAB39957.1| GABA-A	91	32.10888	232	64.87902	0.494	-1.01478	8.17E-08	1.79E-

02			receptor epsilon-like subunit					904		09
OS05T0562300-01	Similar to DnaJ-like protein.	1453	gi|51854270|gb|AAU10651.1| putative heat shock protein, hsp40	30	8.050107	103	21.90535	0.367494 95	-1.4442	3.87E-06	1.19E-07
OS01T0571300-01	Similar to Heat shock transcription factor 31 (Fragment).	1527	gi|52076304|dbj|BAD45089.1| heat shock transcription factor HSF8-like	2	0.510666	26	5.26154	0.0970 56	-3.36503	0.000193	8.83E-06
OS01T0106900-02	Similar to 1-deoxy-D-xylulose 5-phosphate reductoisomerase (Fragment).	1696	gi|10945254|dbj|BAB16915.1| putative 1-deoxy-D-xylulose 5-phosphate reductoiso	33	7.586371	106	19.31339	0.392804	-1.34812	1.03E-05	3.42E-07

			merase								
OS04T0538000-01	Tetratricopeptide TPR-1 domain containing protein.	1963	gi|222629282|gb|EEE61414.1| hypothetical protein OsJ_15608	9	1.787591	163	25.65937	0.0696666	-3.8434	3.49E-29	1.31E-31
OS03T0322800-02	Similar to predicted protein.	785	gi|108707888|gb|ABF95683.1| phagocytosis and cell motility protein ELMO1, putative, expressed	23	11.42363	84	33.0665	0.3454474	-1.53335	1.63E-05	5.75E-07
OS07T0267200-01	Conserved hypothetical protein.	1713	gi|255675258|dbj|BAH92545.1| Os04g0266330, partial	6	1.365651	34	6.133384	0.2222659	-2.16709	0.000961	5.63E-05
OS0	Similar to RNA binding protein.	1	gi|5109087	16	3.846052	58	11.04983	0.	-1.5225	0.0006	3.

6T0600700-02		622	3|dbj|BAD35446.1| agenet domain-containing protein-like					3480664	7	53	58E-05
OS10T0415900-01	Similar to General control of amino-acid synthesis 5-like 2 (Fragment).	1691	gi|78708640|gb|ABB47615.1| acetyltransferase, GNAT family protein, expressed	48	11.06735	151	27.59383	0.4010081	-1.31804	9.96E-08	2.222E-09
OS03T0218500-01	Similar to 70kD heat shock protein.	2225	gi|108706874|gb|ABF94669.1| dnaK protein, expressed	13	2.278029	55	7.638556	0.2982228	-1.74551	0.000195	9.04E-06
OS12T0476200-	Sucrose transporter, TAL effector PthXo2-dependent disease susceptibility to bacterial pathogen	1653	gi|77555420|gb|ABA98216.1| N3 like protein,	48	11.32177	148	27.66734	0.409	-1.28908	2.47E-07	5.88E-

01			putative, expressed					211			09
OS06T0591400-01	Conserved hypothetical protein.	774	gi|50725379|dbj|BAD32853.1| unknown protein	435	219.1262	1391	555.3474	0.3945757 5	-1.34163	1.19E-72	1.21E-75
OS09T0548400-01	Dimethylaniline monooxygenase, N-oxide-forming domain containing protein.	1851	gi|50725113|dbj|BAD33730.1| flavin containing monooxygenase 3 -like	59	12.42772	159	26.54418	0.468819	-1.09483	3.81E-06	1.16E-07
OS02T0773732-01	Hypothetical conserved gene.	2713	gi|46805768|dbj|BAD17136.1| hypothetical protein	40	5.748522	116	13.21255	0.435508	-1.20065	2.95E-05	1.10E-06
OS08T0205	Hypothetical conserved gene.	937	hypothetical protein OsJ_26407	50	20.80542	254	83.76696	0.24	-2.00942	2.02E-23	9.53

800-01								8373			E-26
OS01T0187900-01	Similar to Transcription factor MYBS2.	14332	gi|55771329|dbj|BAD72254.1| putative MybSt1	31	8.440432	108	23.30555	0.36216 4	-1.46528	1.51E-06	4.22E-08
OS06T0694200-01	Esterase, SGNH hydrolase-type domain containing protein.	1384	gi|53792843|dbj|BAD53876.1| putative lipase	2	0.56343	26	5.805182	0.0970566	-3.36503	0.000193	8.83E-06
OS06T0143100-01	Similar to Cadmium tolerant 1.	491	gi|55295808|dbj|BAD67659.1| unknown protein	131	104.0245	400	251.7428	0.4132177	-1.27503	3.70E-19	2.37E-21
OS0	Similar to Heat stress transcription	2	gi|3001758	8	1.528245	272	41.18172	0.	-4.7520	1.60E-	2.

3T0745000-01	factor A-2a.	041	3|gb|AAP13005.1| putative heat shock factor					03711	6	54	37E-57
OS03T0187300-01	Similar to transducin family protein / WD-40 repeat family protein.	3529	expressed protein	78	8.617652	203	17.77555	0.48484804	-1.04453	3.36E-07	8.29E-09
OS01T0627550-00	Hypothetical protein.	1631	no hit	71	16.97268	191	36.18745	0.46690221	-1.09228	2.80E-07	6.76E-09
OS04T0671200-02	Similar to H0624F09.9 protein.	1322	gi|32488409|emb|CAE02834.1|OSJNBa0043A12.39	35	10.32244	97	22.67352	0.45552664	-1.13522	0.000393	2.03E-05

OS02T0259900-01	Conserved hypothetical protein.	1555	hypothetical protein OsJ_06145	7	1.755148	150	29.80846	0.0588881	-4.08606	6.80E-28	2.68E-30
OS03T0796000-02	Similar to Ripening-associated protein (Fragment).	1095	gi|50400030|gb|AAT76418.1| expressed protein	66	23.50043	296	83.53264	0.2813332	-1.82965	4.29E-24	1.98E-26
OS08T0478000-01	Similar to mucin-2.	741	gi|125603761|gb|EAZ43086.1| hypothetical protein OsJ_27677	18	9.471097	84	35.02996	0.2703771	-1.88699	2.95E-07	7.18E-09
OS05T0220600-01	Peptidase S54, rhomboid domain containing protein.	1333	gi|46981237|gb|AAT07555.1| unknown protein	12	3.509919	54	12.51821	0.28803	-1.83452	0.000122	5.32E-0

								85			6
OS12T0514500-03	Similar to ATP binding.	793	hypothetical protein OsI_38512	71	34.9085	184	71.70067	0.486864	-1.03841	1.69E-06	4.77E-08
OS01T0959200-01	Similar to Ci21A protein.	657	gi|222619909|gb|EEE56041.1| hypothetical protein OsJ_04835	6	3.560671	54	25.39844	0.140193	-2.83452	1.46E-07	3.34E-09
OS09T0570100-01	Protein kinase, catalytic domain domain containing protein.	1352	hypothetical protein OsJ_30416	27	7.786335	80	18.28487	0.425835	-1.23163	0.000662	3.64E-05
OS06T0198	RmlC-like jelly roll fold domain containing protein.	698	gi|51091823|dbj|BAD36637.1|	7	3.910107	38	16.82313	0.23	-2.10517	0.000539	2.90

500-00			unknown protein					2424			E-05
OS05T0408300-01	Similar to Lipase.	1997	gi|49328153|gb|AAT58849.1| unknown protein	167	32.60501	432	66.84736	0.48775 3	-1.03578	7.53E-15	6.91E-17
OS01T0963600-01	ABA/WDS induced protein family protein.	952	gi|15289937|dbj|BAB63632.1| putative bundle sheath cell specific protein 1	53	21.70626	407	132.1101	0.1643044	-2.60556	1.21E-51	2.20E-54
OS07T0661600-03	Similar to RNA-binding protein.	2952	gi|38175742|dbj|BAC84316.2| zinc finger protein family-like	44	5.811421	121	12.66624	0.4588812	-1.12403	5.86E-05	2.34E-06

OS05T0105200-01	Similar to Ras-related protein Rab-1B.	600	gi|222629897|gb|EEE62029.1| hypothetical protein OsJ_16811	40	25.9929	106	54.59253	0.47612 6	-1.07059	0.000413	2.15E-05
OS05T0543700-02	Similar to Chaperone protein dnaJ.	1390	gi|52353408|gb|AAU43976.1| putative DnaJ	63	17.67143	161	35.79231	0.49372 22	-1.01823	1.45E-05	5.02E-07
OS01T0248500-01	Similar to Pathogen-related protein.	1312	gi|5042456|gb|AAD38293.1|AC007789_19 putative pathogenesis related protein	174	51.70844	533	125.5371	0.41118 98	-1.27964	1.08E-25	4.63E-28
OS01T0740651-	Hypothetical protein.	1651	no hit	7	1.653092	39	7.299551	0.2266	-2.14264	0.000362	1.84E-

00								465			05
OS02T0686100-00	Similar to RING-H2 finger protein ATL1B.	1051	gi|41052716|dbj|BAD07573.1| putative ring finger protein	138	51.19439	435	127.8984	0.40002744	-1.32094	5.22E-22	2.73E-24
OS09T0526600-02	Similar to Heat shock factor protein 3 (HSF 3) (Heat shock transcription factor 3) (HSTF 3).	1756	gi|52077316|dbj|BAD46357.1| putative heat shock factor	13	2.886455	97	17.0697	0.1690098	-2.56407	4.60E-12	5.72E-14
OS06T0143750-00	Hypothetical protein.	2212	no hit	31	5.464149	99	13.83021	0.3950888	-1.33975	2.55E-05	9.39E-07
OS06T0	Similar to DNAJ heat shock N-terminal domain-containing protein.	82	hypothetical protein	6	2.825315	56	20.89952	0.1	-2.88699	5.65E-08	1.2

<table>
<tr><td>195800-01</td><td></td><td>8</td><td>OsI_22007</td><td></td><td></td><td></td><td></td><td>35186</td><td></td><td></td><td>1E-09</td></tr>
<tr><td>OS08T0368000-01</td><td>Similar to Coatomer delta subunit (Delta-coat protein) (Delta-COP).</td><td>2579</td><td>gi|255678395|dbj|BAH94268.1| Os08g0368000</td><td>258</td><td>39.00447</td><td>661</td><td>79.20065</td><td>0.492477</td><td>-1.02187</td><td>3.37E-22</td><td>1.73E-24</td></tr>
<tr><td>OS09T0555500-01</td><td>Similar to Chloroplast phytoene synthase 3.</td><td>1485</td><td>chloroplast phytoene synthase 3 precursor</td><td>42</td><td>11.02729</td><td>115</td><td>23.9304</td><td>0.460807</td><td>-1.11777</td><td>0.000106</td><td>4.59E-06</td></tr>
<tr><td>OS01T0767600-01</td><td>Conserved hypothetical protein.</td><td>1526</td><td>gi|22535590|dbj|BAC10765.1| unknown protein</td><td>35</td><td>8.942511</td><td>122</td><td>24.70495</td><td>0.361973</td><td>-1.46605</td><td>2.37E-07</td><td>5.60E-09</td></tr>
</table>

OS08T0296900-02	Hypothetical gene.	1331	no hit	33	9.666781	117	27.16354	0.3558873	-1.49056	3.08E-07	7.54E-09
OS01T0971000-02	Conserved hypothetical protein.	532	gi|15289973|dbj|BAB63668.1| unknown protein	187	137.049	830	482.1088	0.284227	-1.81467	2.92E-67	3.24E-70
OS02T0644100-01	Similar to Heat shock protein STI (Stress inducible protein) (GmSTI).	2112	gi|49388654|dbj|BAD25789.1| putative stress-induced protein sti1	406	74.95112	1037	151.7272	0.4939986	-1.01746	1.10E-34	3.23E-37
OS01T0693300-01	Similar to Lipid phosphate phosphatase 2 (EC 3.1.3.-) (AtLPP2) (Phosphatidic acid phosphatase 2) (AtPAP2) (Prenyl diphosphate phosphatase).	2055	gi|56784972|dbj|BAD82502.1| phosphatidic acid phosphatas	51	9.676189	130	19.54835	0.494498	-1.01454	0.00015	6.72E-06

			e beta-like					7			
OS03T0283100-01	Similar to In2-1 protein.	1250	IN2-1 protein, putative, expressed	46	14.34808	123	30.40701	0.47718 68	-1.08355	9.04E-05	3.81E-06
OS10T0538200-01	Peptidase aspartic, catalytic domain containing protein.	1401	gi|21717168|gb|AAM76361.1|AC074196_19 putative nucleoid DNA binding protein	43	11.96675	129	28.45314	0.420578	-1.24956	3.73E-06	1.14E-07
OS01T0132000-01	Similar to Wound-induced protease inhibitor (WIP1).	564	gi|9049425|dbj|BAA99380.1| putative wound-induced protease inhibitor	32	22.12162	237	129.8518	0.170361	-2.55334	2.78E-29	1.03E-31
OS1	Similar to Oxidoreductase, 2OG-Fe	2	gi|1416532	11	2.062929	49	7.283166	0.	-1.8198	0.0003	1.

0T0558700-01	oxygenase family protein, expressed.	079	2|gb|AAK55454.1|AC069300_9 putative dioxygenase					2832246	7	22	62E-05
OS07T0164800-01	Conserved hypothetical protein.	1293	gi|22202765|dbj|BAC07421.1| Neurofilament triplet M protein-like protein	1334	402.2567	4853	1159.819	0.34682 7	-1.52771	0	0
OS05T0542500-02	LEA-like protein.	968	gi|1486503|gb|AAC03364.1| LEA-like protein	6	2.416695	46	14.68456	0.1645744	-2.60319	5.71E-06	1.81E-07
OS08T0331800-01	Conserved hypothetical protein.	562	gi|38424015|dbj|BAD01675.1| hypothetical protein	12	8.325128	50	27.49238	0.302281	-1.72349	0.000511	2.73E-05

OS05T0460000-01	Similar to 70 kDa heat shock cognate protein 1.	2295	gi|47900318|gb|AAT39165.1| putative hsp70	137	23.27469	608	81.86523	60.28843053	-1.81449	3.45E-49	6.48E-52
OS08T0546800-01	Similar to Heat stress transcription factor B-2b.	1498	gi|42408097|dbj|BAD09238.1| putative heat shock factor RHSF2	3	0.780828	41	8.457668	0.09232322	-3.43718	6.90E-07	1.81E-08
OS08T0196700-01	HAP2 subunit of HAP complex, Nuclear Factor Y (NF-YA) transcription factor, Drought stress tolerance	927	gi|38637163|dbj|BAD03416.1| putative CCAAT box binding factor/transcription factor Hap2a	19	7.991345	94	31.33479	0.2550331	-1.97125	1.49E-08	2.93E-10
OS0	Similar to Isoform 2 of Heat stress	1	putative	13	3.436349	90	18.85511	0.	-2.4560	1.09E-	1.

9T0526600-03	transcription factor B-2c.	475	heat shock factor					18225	1	10	62E-12
OS09T0425200-00	Conserved hypothetical protein.	694	gi|50725911|dbj|BAD33439.1| fibroin heavy chain precursor (Fib-H) (H-fibroin)-like protein	137	76.96745	399	177.661	0.4332227	-1.20681	1.57E-17	1.14E-19
OS04T0504000-01	Similar to Adenine phosphoribosyltransferase form 2.	19552	OSJNBa0014K14.11	63	12.58365	165	26.12058	0.4817553	-1.05364	5.33E-06	1.68E-07
OS05T0142400-01	Conserved hypothetical protein.	706	gi|46485813|gb|AAS98438.1| unknown protein	139	76.76373	382	167.2004	0.45591	-1.12308	5.65E-15	5.10E-1

								12			7
OS05T0278500-01	Transferase family protein.	1551	gi|113578633|dbj|BAF16996.1|Os05g0278500	103	25.89235	286	56.98136	0.45444	-1.13796	1.68E-11	2.27E-13
OS07T0170100-01	Similar to Branched chain alpha-keto acid dehydrogenase E1 beta subunit.	1383	gi|50509739|dbj|BAD31791.1|putative branched-chain alpha-keto acid decarboxylase E1 beta subunit	48	13.5321	122	27.2594	0.4964419	-1.01037	0.000281	1.38E-05
OS06T0159900-01	Similar to Arm repeat containing protein.	1315	gi|55296059|dbj|BAD67621.1|protein kinase-like	58	17.19682	431	101.2815	0.169792	-2.55816	1.46E-53	2.29E-56

OS01T0155800-01	Conserved hypothetical protein.	687	gi|15528626|dbj|BAB64647.1| unknown protein	33	18.72851	191	85.91228	0.2179996	-2.19763	9.86E-20	5.98E-22
OS03T0266900-02	Low molecular mass heat shock protein Oshsp17.3.	753	gi|1815660|gb|AAC78392.1| low molecular mass heat shock protein Oshsp17.3	7	3.624508	165	67.7123	0.0535528	-4.22356	2.78E-31	9.49E-34
OS04T0142400-01	Conserved hypothetical protein.	1005	gi|38346965|emb|CAE02262.2| OSJNBb0049I21.1	203	78.75461	571	175.5693	0.4485667	-1.1566	1.93E-23	9.03E-26
OS11T0471200-	Conserved hypothetical protein.	755	gi|113645098|dbj|BAF28239.1| Os11g0471	82	42.34605	216	88.40675	0.478	-1.06193	7.76E-08	1.69E-

01			200, partial					991		09
OS10T0535800-01	Protein of unknown function Cys-rich domain containing protein.	983	gi|31433269|gb|AAP54807.1| Protein of unknown function, DUF614 containing protein, expressed	26	10.31254	78	24.51996	0.4205 78	-1.24956	0.000675	3.733E-05
OS04T0149400-01	Conserved hypothetical protein.	396	hypothetical protein OsJ_13694	142	139.8103	692	539.9947	0.258 891	-1.94947	9.47E-62	1.255E-64
OS12T0133100-02	Similar to cDNA clone:J023128K07, full insert sequence.	2157	transporter, putative, expressed	63	11.38771	165	23.63809	0.4817 53	-1.05364	5.33E-06	1.688E-07

OS01T0837000-01	Ankyrin repeat containing protein.	20339	gi|125528302|gb|EAY76416.1| hypothetical protein OsI_04347	101	19.31302	262	39.7066	0.4863933	-1.03981	3.80E-09	6.93E-11
OS09T0109600-01	Conserved hypothetical protein.	604	gi|46806423|dbj|BAD17580.1| unknown protein	10	6.45519	97	49.62647	0.1300776	-2.94258	6.69E-14	6.69E-16
OS01T0717000-02	Similar to Choline kinase.	16770	choline kinase-like	114	26.61548	308	56.99186	0.4670005	-1.09849	1.07E-11	1.41E-13
OS02T0668000-01	Membrane protein, At2g17000, predicted domain containing protein.	3347	gi|50252134|dbj|BAD28130.1| mechanosensitive ion	34	3.960675	101	9.324902	0.4247	-1.23534	7.97E-05	3.31E-0

			channel domain-containing protein-like					42			6
OS10T0450900-02	Similar to Glycine-rich cell wall structural protein 2 precursor.	1065	gi|31432403|gb|AAP54040.1| transposon protein, putative, CACTA, En/Spm sub-class, expressed	80	29.28777	208	60.35209	0.48528282	-1.04311	2.39E-07	5.66E-09
OS04T0136700-01	Cystathionine beta-synthase, core domain containing protein.	962	gi|38347506|emb|CAE02417.2| OSJNBa0095E20.4	36	14.59061	141	45.29212	0.32221145	-1.63422	7.04E-10	1.17E-11
OS01T0283500-02	Peptidase S54, rhomboid domain containing protein.	1445	gi|13486724|dbj|BAB39959.1| OSJNBa0004B13.13	37	9.983432	106	22.66818	0.44404	-1.18306	9.74E-05	4.14E-0

								16			6
OS08T0485400-01	Similar to Oxidoreductase.	1577	gi|42409409|dbj|BAD10722.1| 2-nitropropane dioxygenase-related protein-like	45	11.12569	139	27.23715	0.40084775	-1.29168	6.20E-07	1.61E-08
OS06T0660700-02	Similar to Ubiquitin carrier protein.	13442	gi|125598111|gb|EAZ37891.1| hypothetical protein OsJ_22240	67	19.46562	172	39.60541	0.49148 9	-1.02477	5.61E-06	1.77E-07
OS09T0409950-01	Hypothetical conserved gene.	745	hypothetical protein OsI_31331	119	62.27829	301	124.8501	0.49888 25	-1.0034	6.84E-10	1.13E-11
OS06T0	Similar to mechanosensitive ion channel domain-containing protein.	24	gi|51091457|dbj|BAD3	29	4.573993	83	10.37548	0.4	-1.18165	0.000823	4.6

205 600-00		72	6198.1| mechanosensitive ion channel domain-containing protein-like					40846			9E-05
OS03T0216766-01	Hypothetical gene.	5476	no hit	132	9.398455	387	21.83867	0.43030358	-1.21639	3.15E-17	2.39E-19
OS06T0533700-00	Hypothetical conserved gene.	222	CLE family OsCLE602 protein	0.5	0.878138	20	27.83913	0.03315443	-4.98652	0.000266	1.30E-05
OS04T0578550-00	Hypothetical conserved gene.	798	hypothetical protein OsI_17083	9	4.397295	119	46.08108	0.09554	-3.38949	2.35E-19	1.49E-2

OS11T0699532-00	Hypothetical gene.	815	transposon protein, putative, CACTA, En/Spm sub-class	39	18.65748	117	44.36156	250.42057 8	-1.24956	1.29E-05	14.40E-07
OS03T0197100-01	Similar to Sugar transporter protein.	2253	gi|108706669|gb|ABF94464.1| mannitol transporter, putative, expressed	13	2.249718	142	19.47627	0.1155111	-3.1139	1.89E-21	1.04E-23
OS01T0757200-01	GA 2-oxidase3, GA metabolism	1748	gi|20161223|dbj|BAB90150.1| gibberellin 2-oxidase	330	73.6069	984	173.9531	0.42231422	-1.24079	2.12E-45	4.51E-48
OS06T0116	Similar to GFA2.	168	gi|55296201|dbj|BAD67919.1|	56	12.98872	151	27.75798	0.446	-1.09564	7.32E-06	2.38

800-01		1	putative GFA2					7927			E-07
OS11T0592200-01	Similar to Chitin-binding allergen Bra r 2 (Fragments).	655	gi|16024932|gb|AAL11444.1| pathogenesis-related protein	18	10.71463	80	37.74221	0.283389	-1.8166	1.35E-06	3.73E-08
OS08T0296900-03	Similar to Heat shock protein 70.	2346	gi|50508716|dbj|BAD31284.1| putative octicosapeptide/Phox/Bem1p (PB1) domain-/tetratricopeptide repeat (TPR)-containing protein	78	12.96321	211	27.79285	0.4666423	-1.10029	4.17E-08	8.85E-10
OS09T0445	Similar to oxidoreductase/ transition metal ion binding protein.	745	gi|51536148|dbj|BAD38322.1|	9	4.710123	60	24.88706	0.18	-2.40156	4.91E-07	1.25

600-01			unknown protein					926			E-08
OS02T0622500-01	Conserved hypothetical protein.	23822	hypothetical protein OsJ_07575	47	7.693113	187	24.25931	0.317121	-1.6569	2.86E-13	3.03E-15
OS01T0719125-00	Hypothetical protein.	2638	no hit	134	19.80505	433	50.72146	0.390467	-1.35673	8.05E-23	3.94E-25
OS06T0125800-01	Zinc finger, RING/FYVE/PHD-type domain containing protein.	10557	gi|6983871|dbj|BAA90806.1| zinc finger protein-like	238	87.79059	696	203.4758	0.431455	-1.21272	6.88E-31	2.39E-33
OS07T0633	Similar to SC35-like splicing factor SCL30a, 30a kD.	103	hypothetical protein OsJ_25252	26	9.756719	85	25.28029	0.38	-1.37354	8.66E-05	3.63

200-02		9						5942			E-06
OS02T0782500-01	Similar to Small heat stress protein class CIII.	829	gi|47497479|dbj|BAD19533.1| putative 17.8 kDa class II heat shock protein	4	1.881271	36	13.4192	0.1401930	-2.83452	3.62E-05	1.37E-06
OS04T0209200-01	Similar to MRP-like ABC transporter.	4081	gi|38346012|emb|CAE01891.2| OSJNBa0035O13.14	29	2.770623	97	7.344864	0.3777219	-1.40653	1.37E-05	4.72E-07
OS05T0407100-01	Four F5 protein family protein.	468	gi|113579069|dbj|BAF17432.1| Os05g0407100	18	14.9959	91	60.08612	0.2495574	-2.00246	1.88E-08	3.73E-10

OS03T0835400-01	Similar to Uvs101.	1368	gi|40714678|gb|AAR88584.1| putative flavoprotein alpha-subunit, having alternative splicing products	42	11.97041	111	25.07353	0.4777412	-1.06669	0.000295	1.46E-05
OS07T0679300-01	Similar to Alpha-galactosidase precursor (EC 3.2.1.22) (Melibiase) (Alpha-D- galactoside galactohydrolase).	1627	gi|33146441|dbj|BAC79549.1| putative alpha-galactosidase	52	12.46125	135	25.6404	0.4860001	-1.04097	6.85E-05	2.79E-06
OS01T0740600-01	Transferrin receptor-like, dimerisation domain containing protein.	468	hypothetical protein OsI_03680	11	9.164163	48	31.69378	0.2891447	-1.79012	0.000463	2.44E-05
OS03T0	Similar to Dihydroxyacetone/glycerone	21	gi|50540688|gb|AAT77	58	10.31652	183	25.79818	0.3	-1.32231	2.33E-09	4.0

719300-01	kinase-like protein.	92	845.1| putative DAK2 domain containing protein					99893			9E-11
OS04T0404400-01	Similar to H0502B11.4 protein.	11137	gi|38569173|emb|CAE05363.3|OJ000315_02.8	49	16.8028	145	39.40816	0.4263779	-1.22979	1.04E-06	2.81E-08
OS10T0482900-01	Thioredoxin fold domain containing protein.	2529	gi|22094367|gb|AAM91894.1| hypothetical protein	48	7.400114	128	15.64011	0.473115	-1.07963	6.43E-05	2.59E-06
OS03T0575200-01	Similar to Potassium transporter 1 (OsHAK1).	2904	gi|30017547|gb|AAP12969.1| putative potassium transporter	37	4.967651	100	10.64099	0.4668441	-1.099	0.000473	2.49E-05

OS01T0757500-02	Similar to Calcyclin-binding protein.	450	gi|57899263|dbj|BAD87508.1| putative calcyclin-binding protein	15	12.99645	78	53.56248	0.24226441	-2.04311	1.81E-07	4.19E-09
OS05T0456900-01	Conserved hypothetical protein.	984	gi|53749364|gb|AAU90223.1| unknown protein	29	11.49076	87	27.32139	0.42057 8	-1.24956	0.000272	1.33E-05
OS03T0113700-01	Similar to Heat shock 70 kDa protein, mitochondrial precursor.	2484	gi|108705828|gb|ABF93623.1| Heat shock 70 kDa protein, mitochondrial precursor, putative, expressed	79	12.39999	246	30.60287	0.4051 91	-1.30333	3.11E-12	3.79E-14
OS1	Similar to root hair defective 3	2	gi|7755175	8	1.120384	63	6.992781	0.	-2.6418	3.56E-	7.

1T0582300-01	GTP-binding (RHD3) family protein.	784	8|gb|ABA94555.1| Root hair defective 3 GTP-binding protein containing protein, expressed					160 22	7	08	46E-10
OS02T0181900-01	Similar to ClpB.	3300	RecName: Full=Chaperone protein ClpB3, mitochondrial; AltName: Full=ATP-dependent Clp protease ATP-binding subunit ClpB homolog 3; AltName: Full=Casein	37	4.371533	266	24.90843	0.1755504	-2.51042	2.94E-32	9.40E-35

OS02T0491400-01	Similar to Peptidylprolyl isomerase.	2340	lytic proteinase B3; Flags: Precursor gi|47848114|dbj|BAD21897.1| putative peptidylprolyl isomerase	256	42.65501	785	103.6651	0.411147	-1.28114	5.18E-38	1.34E-40

Table S7 List of primers used in QPCR analysis

Gene	Forward primer	Reverse primer	Probe	Tm	Length
LOC_Os02g52730	ACATCACCAGCAACTTCCAG	GTACGCGAGATCGTTGATGT	CCACCATCACCAACCTGCCG	60	119
LOC_Os06g23775	CCACCACCACCCTCATCT	TCCCGTACCACCAGATCC	CGTCACTCTCCCACGACGGG	60	113
LOC_Os02g11020	GATGTGCGTAGGCCAGAAC	ATTGCGGGTATAGGAGCATC	ATGGCCATCCTCCTCCAGCG	60	134
LOC_Os07g31500	CCAGATCAATGGAACAGTGC	TACAGGTTGATCAGCTTCGC	CTCCGGCGAGATTCCGGCTA	60	123
LOC_Os07g38630	CCGGTCTCAGGATGAAGG	CGATACTCATGTTCTTTAAACGC	ATCGCCGCGTCCCTCGTC	60	133
LOC_Os10g31500	AGAGCGGCTCGTCGATAG	GTAGCTCGGAGATGCTGCT	CTACAACGGCGGCTACGCCG	60	137
LOC_Os12g42200	ATCATCCTCCAGATCTGCCT	CCAAGAAGGATACCACCGAT	ACCCGCGTCCTCGCCTACCT	60	113
LOC_Os03g48430	GTCCTGGAGCTCCTCTCGT	GCACGGCGGGTAGTAGTT	CTCACCACGCTGACGCCGTC	60	105
LOC_Os01g06010	GCCATGTCCATCATGAACTC	TGGTGGGCTTCTTGTTGTAG	TCTTCGAGAAGCTCGCCGCC	60	94
LOC_Os06g30250	GTGTTCAGGAGGTCGTTCC	AAGATCGTCTCTGCGACCTC	CCTCTTCGCCTTCGCCTCCA	60	140
LOC_Os02g58540	CACCGTCGGGACTAGTACATT	TAGGGAGGTGTGTTGTGGTG	CTGCCCACGCTGCACGATTT	60	129
LOC_Os05g3286	AGGAGGGAGGGCGACTAC	AGCTGCAGGAGGACCTTG	CCTCCTGCAGCTCCTGCGG	60	56

0					
LOC_Os03g23960	CACACTGTCGAAGGAGGAGA	CGACCTTCTCGCTCTGGT	TCCTCCCTGCTCCGGCTCAT	60	110
LOC_Os06g34280	CCGTCTTCCTCCTAGTCGTT	TCTTCGTCCCGTTGTACG	CCGGCGCTGGCTACAGGACT	60	150
LOC_Os08g01450	TAGCAAGCCTTGTGTATCGC	AGATGTGGGACAAGGAGGAG	CGATGCCTCCATTTGCTGCTTG	60	130
LOC_Os12g02060	GGTCTCATGTGCTGACATCC	GTAGGACCTCCTAGCGCAAC	AACAGATTCGCGTGCCGCC	60	66
LOC_Os01g70730	GGTGGTCACCTCCTACGC	TTGTGGAACTGGAGCATCAT	AGTAGCGCTCCCAGCCGAGC	60	117
LOC_Os08g33650	TGGAGCATATGATTGGAGGA	GACAGCATCCCACTTCTCAA	CCATTCCGATCGCAGCCTCA	60	118
LOC_Os02g46100	ATGGCGAGAATGCTCCTT	GAGGTCCGAGTTGAGTGAGTC	CTCCACCGAGCCGGAGGC	60	66
LOC_Os10g28340	TTGTGAAGGAGGAGATGCTG	GTCGTACGTCTTCGTCAGGA	ATCCTCCTGCCGCTGCTGCT	60	125
LOC_Os11g47441	CCCTATTGGGAATTCTTGGA	AGCAGGTTTACGCAAAGGTT	CGTCCGCTACGCAATCGATGTG	60	77
LOC_Os09g25690	CTTAAGCATCGACCTTGGTG	AACCTGACCCTGAACCAGAC	CACCGGCGCCACCACCTAT	60	113
LOC_Os06g10410	ACAAGGAGGAGGTGGATTTG	ATGCCTTTACCACCCAGTTC	TTCCTGGTCTCAAATGCTCCCTCA	60	99
LOC_Os12g03040	AGCTAACAACACCACCACCA	GTCGTTGCTCTTCTTGTGGA	CAGCACCCAGTCGTCCAGCC	60	100

Table S8 Pfam ID of differentially expressed genes

Gene	Pfam ID	result
OS01T0104900-02	PF02458;	up
OS01T0106900-02	PF02670;PF13288;PF08436;	down
OS01T0120400-02	PF01411;PF07973;	down
OS01T0127600-01	PF00228;	up
OS01T0129500-02	PF07891;	down
OS01T0132000-01	PF00228;	down
OS01T0140500-01		up
OS01T0149600-00		up
OS01T0153300-00	PF00125;	up
OS01T0155800-01		down
OS01T0159800-01	PF00010;	down
OS01T0170300-01	PF13855;PF12799;PF00560;PF08263;PF00069;	up
OS01T0172800-01	PF06232;	up
OS01T0179600-01	PF00201;	up
OS01T0184050-00		down
OS01T0184100-01	PF00011;	down
OS01T0186950-00		up
OS01T0187900-01	PF00249;	down
OS01T0223600-01	PF07714;	up
OS01T0238500-01	PF01063;	up
OS01T0239100-01	PF01556;PF00226;	down
OS01T0248500-01		down
OS01T0256500-02	PF03242;	up
OS01T0257100-01	PF05498;	up
OS01T0270300-02	PF00141;	up
OS01T0270501-00		up
OS01T0278950-00	PF00082;PF05922;PF02225;	up
OS01T0280500-01	PF01912;	up
OS01T0282866-00		up
OS01T0283300-02		down
OS01T0283500-02	PF01694;PF00641;	down
OS01T0284500-01	PF00190;	up
OS01T0287600-01	PF00182;	up
OS01T0291733-00		up
OS01T0294700-01	PF00141;	up
OS01T0299400-01	PF09597;	up
OS01T0316600-01	PF00076;	down
OS01T0346250-00		up
OS01T0348900-01	PF01419;	down
OS01T0357100-01	PF01077;PF03460;	up
OS01T0357100-02	PF01077;PF03460;	up
OS01T0357150-00		up

OS01T0502700-00	PF00125;	up
OS01T0510200-01		up
OS01T0524700-01	PF00514;	down
OS01T0571300-01	PF00447;	down
OS01T0574800-01	PF03168;	up
OS01T0580800-01		up
OS01T0582600-01	PF13359;	up
OS01T0607900-01	PF13855;PF08263;PF00069;	up
OS01T0615200-01	PF02458;	up
OS01T0624500-01	PF05002;	down
OS01T0627550-00		down
OS01T0642200-01		down
OS01T0644500-00		up
OS01T0644900-01		up
OS01T0652800-01	PF13839;PF14416;	up
OS01T0667600-01	PF00071;	up
OS01T0667700-04	PF13639;	down
OS01T0668100-01	PF02469;	up
OS01T0693300-01	PF01569;	down
OS01T0717000-02	PF01633;PF04428;	down
OS01T0719100-01	PF05495;	down
OS01T0719100-02	PF14599;PF13639;PF05495;	down
OS01T0719100-03		down
OS01T0719100-04	PF14599;PF13639;PF05495;	down
OS01T0719125-00		down
OS01T0719300-01	PF00916;PF01740;PF13792;	down
OS01T0726100-01	PF01190;	up
OS01T0734800-02	PF00201;	down
OS01T0738000-01		up
OS01T0740600-01	PF04253;	down
OS01T0740651-00		down
OS01T0750100-02	PF03106;	up
OS01T0756900-01		up
OS01T0757200-01	PF14226;PF03171;	down
OS01T0757500-02	PF04969;PF05002;	down
OS01T0763900-01	PF07983;	up
OS01T0765600-01	PF13833;PF00036;	up
OS01T0767600-01		down
OS01T0786500-00	PF02298;	up
OS01T0797600-01	PF00847;	up
OS01T0799500-01	PF03352;	up
OS01T0823900-01	PF04564;PF00514;	up
OS01T0835700-01	PF06203;	up
OS01T0835900-00	PF00125;	up

OS01T0837000-01	PF13606;PF13962;PF12796;	down
OS01T0839900-01	PF00314;	up
OS01T0840100-01	PF00012;	down
OS01T0841000-01	PF01417;	up
OS01T0851600-01	PF02544;	up
OS01T0856500-01	PF01490;	up
OS01T0856500-02	PF01490;	up
OS01T0860601-01	PF00111;	up
OS01T0865900-00		up
OS01T0866200-00		up
OS01T0871500-01	PF00854;	up
OS01T0874300-01	PF00249;	up
OS01T0878700-02	PF01490;	up
OS01T0878800-01	PF02900;	up
OS01T0914200-01		up
OS01T0914300-01	PF14368;	up
OS01T0916100-01		up
OS01T0918400-00		down
OS01T0919600-02	PF05212;	up
OS01T0926400-03	PF03016;	up
OS01T0933075-01		up
OS01T0933500-00		down
OS01T0935800-01	PF07714;	up
OS01T0950300-01		up
OS01T0959200-01	PF02496;	down
OS01T0963600-01	PF02496;	down
OS01T0971000-02		down
OS02T0116750-00		up
OS02T0139000-01	PF14379;PF00249;	down
OS02T0139100-01	PF09229;	down
OS02T0145600-01		up
OS02T0150900-02	PF07800;	down
OS02T0173000-01		up
OS02T0175600-01	PF00828;	up
OS02T0181900-01	PF07724;PF00004;PF02861;PF10431;	down
OS02T0186900-01	PF00067;	up
OS02T0198500-02		up
OS02T0203200-01		up
OS02T0204700-00	PF00067;	up
OS02T0209300-01	PF06376;	down
OS02T0226300-00	PF00153;	down
OS02T0230000-01		up
OS02T0236600-01	PF00141;	up
OS02T0256200-02		up

OS02T0258200-01	PF00505;	up
OS02T0258250-00		up
OS02T0259900-01		down
OS02T0261100-01	PF00179;	down
OS02T0264800-01	PF06376;	up
OS02T0281200-01	PF00931;	down
OS02T0285300-01	PF05558;	up
OS02T0326100-01		up
OS02T0332700-01		up
OS02T0491400-01	PF13414;PF00254;	down
OS02T0528100-02	PF04949;	up
OS02T0538700-01	PF03168;	up
OS02T0543000-03		down
OS02T0548700-00	PF04564;	up
OS02T0564200-01		up
OS02T0575000-01		up
OS02T0581900-01		up
OS02T0583700-00		up
OS02T0585650-01		up
OS02T0585700-00		up
OS02T0606200-01	PF00643;	up
OS02T0611800-01	PF02458;	up
OS02T0620566-00		up
OS02T0620600-01	PF00909;	up
OS02T0622500-01		down
OS02T0625300-00	PF00069;	up
OS02T0629800-01	PF00304;	up
OS02T0644100-01	PF07719;PF00515;PF13414;	down
OS02T0653200-01	PF02298;	up
OS02T0653900-03	PF01764;	up
OS02T0658150-00		up
OS02T0662000-01	PF14547;	up
OS02T0668000-01	PF00924;	down
OS02T0669000-01	PF00657;	up
OS02T0670900-02	PF01490;	up
OS02T0670900-05	PF01490;	up
OS02T0684500-00	PF00125;	up
OS02T0686100-00	PF13639;	down
OS02T0689900-01	PF00854;	up
OS02T0690000-01		up
OS02T0720450-00		up
OS02T0729300-01		up
OS02T0731200-01	PF01486;PF00319;	down
OS02T0734400-00	PF05042;	up

OS02T0740500-01		down
OS02T0743800-02	PF04969;	down
OS02T0749400-01		up
OS02T0753300-01	PF01477;	up
OS02T0758000-01	PF00011;	down
OS02T0761600-01	PF14295;	down
OS02T0765900-00	PF01077;PF03460;	up
OS02T0770800-01	PF00970;PF00174;PF00175;PF00173;PF03404;	up
OS02T0773700-01		down
OS02T0773732-01	PF13839;PF14416;	down
OS02T0779200-01	PF00082;PF05922;PF02225;	up
OS02T0782500-01	PF00011;	down
OS02T0788800-01	PF01490;	down
OS02T0788800-02	PF01490;	down
OS02T0793700-01	PF00173;	up
OS02T0798200-01	PF13639;	up
OS02T0814300-01		up
OS02T0832150-00	PF13639;	up
OS02T0833300-01	PF05743;PF09454;	up
OS03T0101000-01	PF00501;	up
OS03T0102500-01	PF03330;PF01357;	up
OS03T0103100-01	PF14547;	up
OS03T0103200-01	PF14547;	up
OS03T0105633-00		up
OS03T0109500-00	PF00833;	up
OS03T0113000-01	PF00069;	up
OS03T0113700-01	PF00012;	down
OS03T0113750-00		down
OS03T0115000-01	PF02298;	up
OS03T0119900-01	PF00125;	up
OS03T0120400-01	PF00403;	up
OS03T0126600-01		up
OS03T0152000-01	PF00403;	up
OS03T0161900-01		down
OS03T0162200-01	PF00125;	up
OS03T0182800-01	PF00847;	up
OS03T0183200-01		up
OS03T0183300-01	PF00847;	up
OS03T0187300-01	PF00400;	down
OS03T0188500-01		up
OS03T0194350-00		up
OS03T0194900-01	PF04526;PF03188;	up
OS03T0197100-01	PF00083;	down
OS03T0204300-01		up

OS03T0204366-00		up
OS03T0209000-01	PF13943;	up
OS03T0211500-01	PF03763;	down
OS03T0216766-01		down
OS03T0218500-01	PF00012;	down
OS03T0223000-01	PF00069;	up
OS03T0223000-02	PF13855;PF00560;PF08263;PF00069;	up
OS03T0223000-03	PF00069;	up
OS03T0224300-01	PF00982;PF02358;	up
OS03T0239300-01		up
OS03T0241600-01	PF00582;PF00069;	up
OS03T0243900-01	PF00314;	up
OS03T0244200-02	PF00314;	up
OS03T0244950-00	PF00226;	up
OS03T0254400-00		up
OS03T0259500-00	PF01020;	up
OS03T0261100-01		up
OS03T0265666-00		up
OS03T0266300-01	PF00011;	down
OS03T0266300-03	PF00011;	down
OS03T0266900-02	PF00011;	down
OS03T0267000-00	PF00011;	down
OS03T0277300-01	PF00012;	down
OS03T0279200-01	PF00125;	up
OS03T0283100-01	PF13410;PF13417;	down
OS03T0284150-00		down
OS03T0286500-01	PF00076;	up
OS03T0286900-01	PF01679;	down
OS03T0288000-01	PF01439;	up
OS03T0288000-02	PF01439;	up
OS03T0291800-02	PF13839;PF14416;	up
OS03T0293000-01		down
OS03T0293100-01		up
OS03T0297900-00	PF07732;PF00394;	up
OS03T0305150-00		up
OS03T0306800-01	PF02672;	up
OS03T0316200-01	PF01501;	down
OS03T0322800-02	PF04727;	down
OS03T0326000-01		down
OS03T0345901-00		up
OS03T0355700-00	PF00612;PF13178;	up
OS03T0360000-00		down
OS03T0360200-00		up
OS03T0360300-01		up

OS03T0364400-02	PF07714;	up
OS03T0381300-01	PF04969;	down
OS03T0385400-01	PF14368;	up
OS03T0439800-02	PF04419;	up
OS03T0563600-01	PF00141;	up
OS03T0575200-01	PF02705;	down
OS03T0575500-01		up
OS03T0575500-02		up
OS03T0592500-01	PF00504;	up
OS03T0634400-01	PF03822;PF00069;	up
OS03T0640800-02	PF08670;PF01852;	up
OS03T0648400-01	PF00684;PF00226;	up
OS03T0670700-01	PF00076;	up
OS03T0670700-02	PF00076;	up
OS03T0670700-03	PF00076;	up
OS03T0670700-04	PF00076;	up
OS03T0673700-01		up
OS03T0687100-00		up
OS03T0690500-00	PF14226;PF03171;	up
OS03T0692700-05	PF04398;	up
OS03T0694700-01	PF01165;	up
OS03T0708000-01	PF00068;	up
OS03T0708250-00		up
OS03T0719300-01	PF02733;PF02734;	down
OS03T0722200-01		up
OS03T0724700-02	PF02545;	down
OS03T0724700-03	PF02545;	down
OS03T0726100-01	PF00091;PF03953;	up
OS03T0738600-01	PF00305;PF01477;	up
OS03T0738900-00	PF03000;	up
OS03T0743650-01		up
OS03T0745000-01	PF00447;	down
OS03T0745000-02	PF00447;	down
OS03T0748700-01	PF02906;PF02256;	down
OS03T0749300-01	PF01915;PF00933;	up
OS03T0749300-02	PF01915;PF00933;	up
OS03T0749300-03	PF01915;PF00933;	up
OS03T0749300-04	PF01915;	up
OS03T0749401-00		up
OS03T0755700-00	PF00572;	up
OS03T0757950-00		up
OS03T0776900-01	PF00226;	down
OS03T0785100-01		up
OS03T0789433-01		up

OS03T0795300-01	PF00560;	up
OS03T0796000-02	PF12481;	down
OS03T0799000-01	PF00538;	up
OS03T0799050-00		up
OS03T0807150-00		up
OS03T0809000-01	PF03018;	up
OS03T0820500-01	PF00241;	down
OS03T0835400-01	PF01012;PF00766;	down
OS03T0861400-00	PF00403;	up
OS04T0136700-01	PF00571;	down
OS04T0142400-01		down
OS04T0149400-01		down
OS04T0178300-02	PF03936;PF01397;	down
OS04T0191500-01		down
OS04T0208200-01	PF08246;PF00112;	up
OS04T0209200-01	PF00664;PF00005;	down
OS04T0250050-00		up
OS04T0252850-00		up
OS04T0253000-01	PF00538;	up
OS04T0290400-01		up
OS04T0309500-01		up
OS04T0320700-01	PF00201;	up
OS04T0320700-02	PF00201;	up
OS04T0322100-01	PF01657;	up
OS04T0345100-01		up
OS04T0352400-01	PF07719;PF13414;PF00254;	down
OS04T0384000-01		up
OS04T0387600-01	PF11820;	up
OS04T0401751-00		up
OS04T0402700-01		up
OS04T0404400-01	PF13668;	down
OS04T0406600-01	PF00800;	up
OS04T0412100-01		up
OS04T0414700-01		up
OS04T0416700-01	PF03168;	up
OS04T0429450-01		up
OS04T0450300-00	PF13639;	up
OS04T0457000-01	PF00504;	up
OS04T0459200-01		up
OS04T0462900-01	PF01145;	up
OS04T0462900-05	PF01145;	up
OS04T0465100-01	PF00141;	up
OS04T0482300-01	PF02135;PF00651;	up
OS04T0495400-01	PF03351;PF03188;	up

OS04T0495900-01		up
OS04T0496300-01		up
OS04T0497700-01	PF00643;PF06203;	up
OS04T0504000-01	PF00156;	down
OS04T0508500-01	PF00249;	down
OS04T0509600-01	PF00909;	up
OS04T0517000-01		up
OS04T0517500-01	PF00069;	up
OS04T0533900-01	PF00403;	down
OS04T0538000-01	PF07719;PF00515;PF13414;	down
OS04T0539601-00		up
OS04T0543900-02	PF02812;PF00208;	down
OS04T0550866-00		up
OS04T0565500-01	PF01490;	up
OS04T0567800-01	PF02701;	up
OS04T0576900-01	PF13855;PF00560;PF13516;PF00069;	up
OS04T0578550-00		down
OS04T0583600-01	PF00125;	up
OS04T0605100-01	PF03106;PF10533;	up
OS04T0627900-01	PF01253;	up
OS04T0638700-01		down
OS04T0642000-02	PF00664;PF00005;	up
OS04T0651000-01	PF00141;	down
OS04T0659200-01		up
OS04T0671200-01	PF01593;	down
OS04T0671200-02	PF01593;	down
OS04T0671300-01	PF01593;	down
OS04T0671500-00		down
OS04T0677033-01		up
OS04T0684900-01	PF04857;	up
OS04T0684900-02	PF04857;	up
OS04T0688100-01	PF00141;	up
OS04T0688100-02	PF00141;	up
OS04T0689000-01	PF00141;	up
OS05T0105200-01	PF00071;	down
OS05T0113900-01	PF00125;	up
OS05T0114800-01		up
OS05T0128200-01	PF00023;PF00642;	up
OS05T0134400-01	PF00141;	up
OS05T0134400-02	PF00141;	up
OS05T0135200-01	PF00141;	up
OS05T0138300-03		down
OS05T0142100-01		down
OS05T0142400-01		down

OS05T0149300-00	PF03171;	up
OS05T0182900-01		down
OS05T0192700-01		up
OS05T0199100-01	PF03168;	up
OS05T0208500-03		up
OS05T0217000-01	PF06376;	up
OS05T0220600-01	PF01694;	down
OS05T0278500-01	PF02458;	down
OS05T0311550-00		up
OS05T0320750-00		up
OS05T0363200-01	PF01370;	up
OS05T0369300-01	PF03081;	down
OS05T0395400-00		up
OS05T0405000-01	PF01326;PF02896;PF00391;	down
OS05T0405000-02	PF01326;PF02896;PF00391;	down
OS05T0407100-01	PF04419;	down
OS05T0408300-01		down
OS05T0423500-01	PF07714;	up
OS05T0427400-00	PF00221;	up
OS05T0428400-00	PF00582;	up
OS05T0429100-01		up
OS05T0429100-02		up
OS05T0438700-01		up
OS05T0439200-01		up
OS05T0447700-01	PF13432;	up
OS05T0456800-01		down
OS05T0456900-01		down
OS05T0460000-01	PF00012;	down
OS05T0462301-00		down
OS05T0462700-01	PF00125;	up
OS05T0466600-01	PF00125;	up
OS05T0472000-00	PF04852;	up
OS05T0474400-01	PF03208;	up
OS05T0477300-03	PF01283;	up
OS05T0493800-01	PF00892;	up
OS05T0494500-02	PF03151;	up
OS05T0499300-01	PF00141;	up
OS05T0510800-01	PF13641;	up
OS05T0512600-01	PF07983;	up
OS05T0514800-00		up
OS05T0526200-01		up
OS05T0531200-01	PF01190;	up
OS05T0531200-02	PF01190;	up
OS05T0533500-01	PF00132;PF06426;	up

OS05T0539800-02	PF05608;	up
OS05T0541750-01		up
OS05T0542500-01	PF02987;	down
OS05T0542500-02	PF02987;	down
OS05T0543700-02	PF14308;PF00226;	down
OS05T0555800-00	PF01247;	up
OS05T0556400-01	PF03351;PF03188;	up
OS05T0556900-02	PF01247;	up
OS05T0560000-01	PF03244;	up
OS05T0562300-01	PF01556;PF00226;	down
OS05T0563550-00	PF02469;	up
OS05T0563600-01	PF02469;	up
OS05T0595200-01	PF00188;	down
OS05T0596200-03		down
OS06T0101600-01	PF00127;	up
OS06T0114500-01	PF06592;	down
OS06T0115300-01	PF00887;	down
OS06T0115700-02		up
OS06T0116800-01	PF01556;PF00684;PF00226;	down
OS06T0125800-01	PF13639;	down
OS06T0127250-00		up
OS06T0129900-02	PF00067;	down
OS06T0131001-00		up
OS06T0131300-00	PF02784;PF00278;	up
OS06T0133200-01		up
OS06T0143100-01	PF12734;	down
OS06T0143700-01	PF00916;PF01740;PF13792;	down
OS06T0143750-00		down
OS06T0154200-02		down
OS06T0156600-01	PF00657;	up
OS06T0156650-00		up
OS06T0157125-00		up
OS06T0159450-00		up
OS06T0159900-01	PF13414;PF04564;	down
OS06T0160000-00		up
OS06T0160100-00	PF00125;	up
OS06T0163300-01	PF03168;	up
OS06T0195800-01	PF00226;	down
OS06T0198500-00	PF06172;	down
OS06T0199400-00		up
OS06T0199402-00	PF07911;	up
OS06T0202400-01		up
OS06T0205600-00	PF00924;	down
OS06T0232300-01	PF03547;	up

OS06T0240001-01		up
OS06T0246000-01	PF06522;	up
OS06T0257450-00	PF00268;	up
OS06T0266800-01	PF02704;	up
OS06T0268800-01	PF11744;	down
OS06T0297800-01	PF02893;PF00168;	down
OS06T0319366-00		up
OS06T0320500-01	PF00504;	up
OS06T0346201-00		up
OS06T0493100-01		down
OS06T0498000-00		up
OS06T0506600-01	PF00179;	down
OS06T0508800-01		up
OS06T0531900-01	PF00657;	up
OS06T0533700-00		down
OS06T0538400-01		down
OS06T0543200-01	PF00249;	up
OS06T0544100-01	PF13855;PF13516;PF07714;	up
OS06T0545900-01	PF10250;	up
OS06T0548000-01	PF00155;	down
OS06T0562200-01	PF10604;	up
OS06T0565100-00	PF00067;	up
OS06T0567433-00		up
OS06T0591400-01		down
OS06T0600700-02	PF05641;PF03735;	down
OS06T0614400-01		up
OS06T0660700-02	PF00179;	down
OS06T0668250-00		down
OS06T0672400-01	PF04852;	up
OS06T0674100-01		down
OS06T0682900-01	PF02679;	down
OS06T0686400-01	PF14368;	up
OS06T0694200-01	PF00657;	down
OS06T0696450-00		up
OS06T0701700-02	PF02386;	down
OS06T0708600-01		up
OS06T0712600-00	PF05142;	up
OS07T0107300-01	PF03018;	up
OS07T0107366-01		up
OS07T0112700-01	PF02298;	up
OS07T0119400-01	PF07731;PF07732;PF00394;	up
OS07T0125201-00	PF00188;	up
OS07T0126100-00	PF00188;	up
OS07T0126500-00	PF00188;	up

OS07T0129700-01	PF03791;PF03790;PF03789;PF05920;	up
OS07T0148900-01	PF01241;	up
OS07T0150700-01	PF03822;PF00069;	down
OS07T0154300-01		up
OS07T0164800-01		down
OS07T0170100-01	PF02780;PF02779;	down
OS07T0170100-02	PF02780;PF02779;	down
OS07T0176400-00		up
OS07T0182000-01	PF12498;PF00170;	down
OS07T0184800-01	PF00538;	up
OS07T0184850-00		up
OS07T0186000-02	PF00085;	down
OS07T0227600-00	PF00847;	up
OS07T0245100-01	PF00383;	down
OS07T0267200-01		down
OS07T0287400-01	PF14368;	up
OS07T0294300-01		up
OS07T0297400-01		up
OS07T0418600-01		down
OS07T0418700-01		down
OS07T0448300-00		up
OS07T0452500-02	PF04061;	down
OS07T0495400-00		up
OS07T0498400-00	PF13855;PF13504;PF12799;PF08263;PF00069;	up
OS07T0529700-01	PF00722;	up
OS07T0542400-01	PF01657;PF07714;	up
OS07T0545300-01	PF00125;	up
OS07T0549900-00	PF00125;	up
OS07T0558400-01	PF00504;	up
OS07T0562700-02	PF00504;	up
OS07T0568700-02	PF12799;PF08263;	up
OS07T0573900-00		up
OS07T0580800-01		up
OS07T0582700-01		up
OS07T0582800-01		up
OS07T0588900-01	PF03360;	up
OS07T0589200-00	PF03514;	up
OS07T0590700-01		up
OS07T0597400-01		up
OS07T0601000-01	PF01370;	up
OS07T0617700-01	PF00646;	up
OS07T0631500-01		up
OS07T0633200-01	PF00076;	down
OS07T0633200-02	PF00076;	down

OS07T0638400-01	PF10417;PF00578;	up
OS07T0638500-01	PF03018;	up
OS07T0639000-01	PF00141;	up
OS07T0658600-00	PF14543;PF14541;	up
OS07T0661600-01	PF13920;	down
OS07T0661600-03	PF00097;	down
OS07T0676900-01	PF00141;	up
OS07T0677300-01	PF00141;	up
OS07T0679300-01	PF02065;	down
OS08T0105400-00	PF00067;	down
OS08T0110100-01	PF00327;	up
OS08T0112900-01	PF00657;	up
OS08T0112950-00		up
OS08T0113150-00		up
OS08T0129300-00	PF00651;	up
OS08T0129900-00	PF14712;	up
OS08T0130100-01	PF06943;	down
OS08T0143700-01	PF05057;	down
OS08T0155550-00		up
OS08T0163500-01	PF06219;	up
OS08T0175200-02		down
OS08T0196700-01	PF02045;	down
OS08T0205800-01		down
OS08T0235800-00	PF03106;PF10533;	up
OS08T0239300-01	PF01738;	up
OS08T0253800-01	PF13641;	up
OS08T0260600-01	PF00335;	up
OS08T0270900-01	PF12874;	down
OS08T0296900-01	PF13414;PF00564;	down
OS08T0296900-02		down
OS08T0296900-03	PF00564;	down
OS08T0300300-01	PF00571;PF00654;	up
OS08T0320400-01	PF00218;	down
OS08T0331000-01		down
OS08T0331800-01		down
OS08T0335600-01	PF04526;	down
OS08T0337800-00	PF03140;	up
OS08T0368000-01	PF01217;	down
OS08T0389700-02	PF01925;	up
OS08T0407600-01	PF04570;	up
OS08T0412700-01	PF06880;	down
OS08T0416000-02	PF02183;PF00046;	up
OS08T0427700-00	PF00125;	up
OS08T0433350-00	PF02532;	down

OS08T0462900-01	PF04819;	up
OS08T0464000-01	PF09229;PF08327;	down
OS08T0468100-01	PF00970;PF00174;PF00175;PF00173;PF03404;	up
OS08T0468200-01		up
OS08T0468801-01		up
OS08T0469600-01		up
OS08T0478000-01		down
OS08T0481400-01	PF02183;PF00046;	up
OS08T0485400-01	PF03060;	down
OS08T0485400-02	PF03060;	down
OS08T0500700-01	PF00183;PF02518;	down
OS08T0500800-01	PF09409;PF08325;	down
OS08T0502400-01	PF02469;	up
OS08T0502700-01	PF00266;	up
OS08T0503000-01		up
OS08T0510700-01	PF00439;PF00249;	up
OS08T0524400-00	PF04526;	up
OS08T0525600-01	PF13414;PF00254;	down
OS08T0530033-00		down
OS08T0532350-00		up
OS08T0546800-01	PF00447;	down
OS08T0554400-02	PF12171;	up
OS08T0557850-00		up
OS08T0561500-01	PF00892;	up
OS09T0109600-01		down
OS09T0293601-01		up
OS09T0300800-01	PF03140;	up
OS09T0319701-01		up
OS09T0346500-04	PF00504;	up
OS09T0362800-01	PF11838;	down
OS09T0365100-01		up
OS09T0367900-02	PF04570;	up
OS09T0369050-01		up
OS09T0369400-01	PF02358;	up
OS09T0379600-00	PF02183;PF00046;	up
OS09T0392666-01		up
OS09T0396900-01	PF01988;	down
OS09T0397700-01	PF04828;	up
OS09T0400600-01		down
OS09T0403300-00	PF00067;	up
OS09T0409950-01		down
OS09T0412700-01		down
OS09T0420300-01	PF03352;	up
OS09T0425200-00		down

OS09T0433600-01	PF00125;	up
OS09T0436500-03		up
OS09T0440851-01		down
OS09T0445600-01		down
OS09T0452700-00	PF00240;PF01020;	up
OS09T0464000-01	PF00484;	down
OS09T0470500-01	PF02183;PF00046;	up
OS09T0481200-01	PF01241;	up
OS09T0483150-00		up
OS09T0483400-01		up
OS09T0484200-01		up
OS09T0485050-00		up
OS09T0498100-01	PF00886;	up
OS09T0526600-02	PF00447;	down
OS09T0526600-03	PF00447;	down
OS09T0528100-01		up
OS09T0535400-00		up
OS09T0548400-01	PF00743;	down
OS09T0553900-01	PF10358;	up
OS09T0555500-01	PF00494;	down
OS09T0563700-01	PF10539;	down
OS09T0570100-01	PF00069;	down
OS10T0114400-01	PF13855;PF00560;PF13516;PF08263;PF00069;	up
OS10T0155100-01		up
OS10T0169200-01		up
OS10T0177200-02	PF13405;	down
OS10T0191300-01	PF00188;	up
OS10T0205200-01	PF08694;	up
OS10T0213800-01		up
OS10T0330000-01		up
OS10T0330400-01	PF02622;	up
OS10T0376200-01	PF12799;PF00560;	up
OS10T0377300-01	PF02183;PF00046;	up
OS10T0408700-02	PF00481;	up
OS10T0411800-01	PF00833;	up
OS10T0412700-01	PF00314;	up
OS10T0413101-01		up
OS10T0415900-01	PF00439;PF00583;	down
OS10T0418000-01	PF00125;	up
OS10T0418300-01		up
OS10T0419300-00	PF00447;	down
OS10T0450900-02		down
OS10T0452500-00		up
OS10T0454200-01		up

OS10T0482900-01	PF04784;PF00462;PF00610;	down
OS10T0492900-01	PF02065;	down
OS10T0493300-01		up
OS10T0506100-01	PF00403;	down
OS10T0524400-01	PF00168;PF12357;PF00614;	down
OS10T0529800-00	PF00043;PF13417;	up
OS10T0534900-01	PF07526;PF05920;	up
OS10T0535800-01	PF04749;	down
OS10T0536700-01	PF00141;	up
OS10T0538200-01	PF14543;PF14541;	down
OS10T0539500-01	PF00125;	up
OS10T0550200-01	PF12274;	up
OS10T0551401-00		up
OS10T0552600-01	PF14547;	up
OS10T0552700-01	PF14547;	up
OS10T0552800-01	PF14547;	up
OS10T0554200-01	PF00854;	up
OS10T0554200-02	PF00854;	up
OS10T0554800-01		up
OS10T0555900-01	PF03330;PF01357;	up
OS10T0556100-01	PF03330;PF01357;	up
OS10T0556100-03	PF03330;	up
OS10T0556200-01	PF00010;	up
OS10T0558700-01	PF14226;	down
OS10T0569400-01		down
OS10T0569800-00		down
OS10T0573700-01	PF00153;	up
OS10T0573700-02	PF00153;	up
OS11T0100300-00	PF13515;	up
OS11T0108800-00	PF00069;	up
OS11T0112200-01	PF00141;	down
OS11T0116300-01	PF02431;	down
OS11T0118300-01	PF03000;	up
OS11T0139900-00	PF02330;PF06094;	up
OS11T0141800-01		up
OS11T0149100-01	PF03479;	up
OS11T0151300-01	PF00467;	up
OS11T0169200-00	PF01490;	up
OS11T0171300-01	PF00274;	up
OS11T0178800-01	PF03018;	up
OS11T0197600-01	PF02362;	down
OS11T0220700-01	PF14303;	up
OS11T0242800-01	PF00504;	up
OS11T0246200-00	PF01344;PF00646;	up

OS11T0247300-01	PF00091;PF03953;	up
OS11T0247350-00		up
OS11T0255500-01	PF00107;	down
OS11T0256900-01	PF03492;	up
OS11T0258900-01		up
OS11T0297000-01		up
OS11T0406945-00	PF02567;	down
OS11T0428800-01	PF02298;	down
OS11T0453900-01	PF00257;	down
OS11T0471200-01		down
OS11T0474100-01		up
OS11T0474566-01		up
OS11T0475500-01		up
OS11T0502700-01		down
OS11T0515000-00		up
OS11T0546750-00		up
OS11T0582300-01	PF05879;	down
OS11T0592200-01	PF00967;	down
OS11T0609600-01	PF00244;	down
OS11T0622800-00	PF00107;PF08240;	down
OS11T0636600-02		up
OS11T0697600-00		down
OS11T0699532-00		down
OS11T0703900-01	PF00012;	up
OS11T0703900-02	PF00012;	up
OS11T0707000-03	PF00004;	up
OS12T0100200-00	PF13515;	up
OS12T0111800-00	PF00141;	down
OS12T0117500-01		up
OS12T0123700-00	PF02365;	down
OS12T0124200-01	PF00380;	up
OS12T0133100-02	PF07690;	down
OS12T0150100-01	PF00467;	up
OS12T0166700-01		up
OS12T0171500-01		up
OS12T0183100-01	PF00676;	down
OS12T0189400-01	PF05479;	up
OS12T0235200-01		up
OS12T0292301-00		up
OS12T0425800-01		up
OS12T0438000-02	PF00125;	up
OS12T0448900-01	PF03098;	down
OS12T0454600-00	PF02298;	up
OS12T0466200-01		up

OS12T0476200-01	PF03083;	down
OS12T0478200-01		down
OS12T0478200-02	PF02893;	down
OS12T0488900-01	PF00806;	down
OS12T0514500-03	PF00183;	down
OS12T0516200-00		down
OS12T0548501-01	PF00280;	up
OS12T0555100-01	PF00407;	up
OS12T0569200-01		up
OS12T0569300-01	PF00314;	up
OS12T0594950-01		down
OS12T0603700-00	PF00069;	up
OS12T0616500-00	PF00999;	up
OS12T0632000-01	PF00076;	up
OS12T0635400-01	PF05678;	down

Figure S9 Protein network of differentially expressed genes

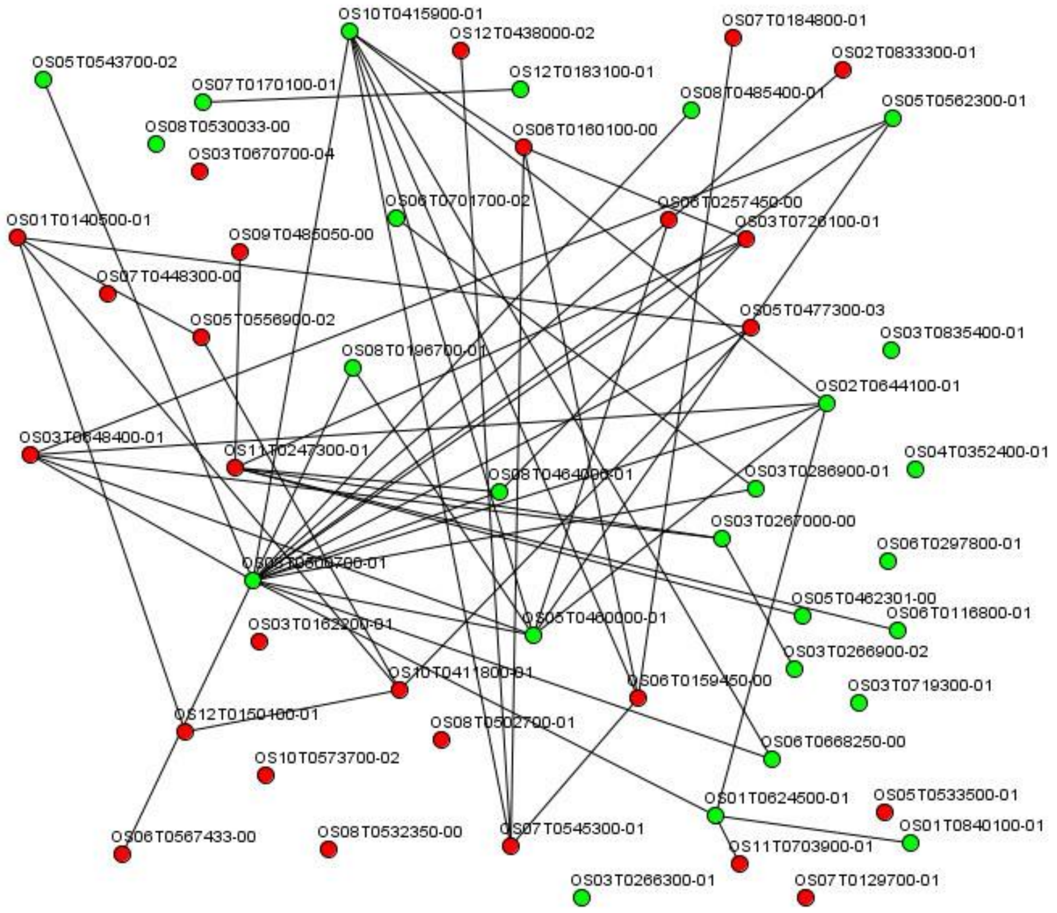

Figure S10 Expression analysis of differentially expressed genes from different tissue using microarray data

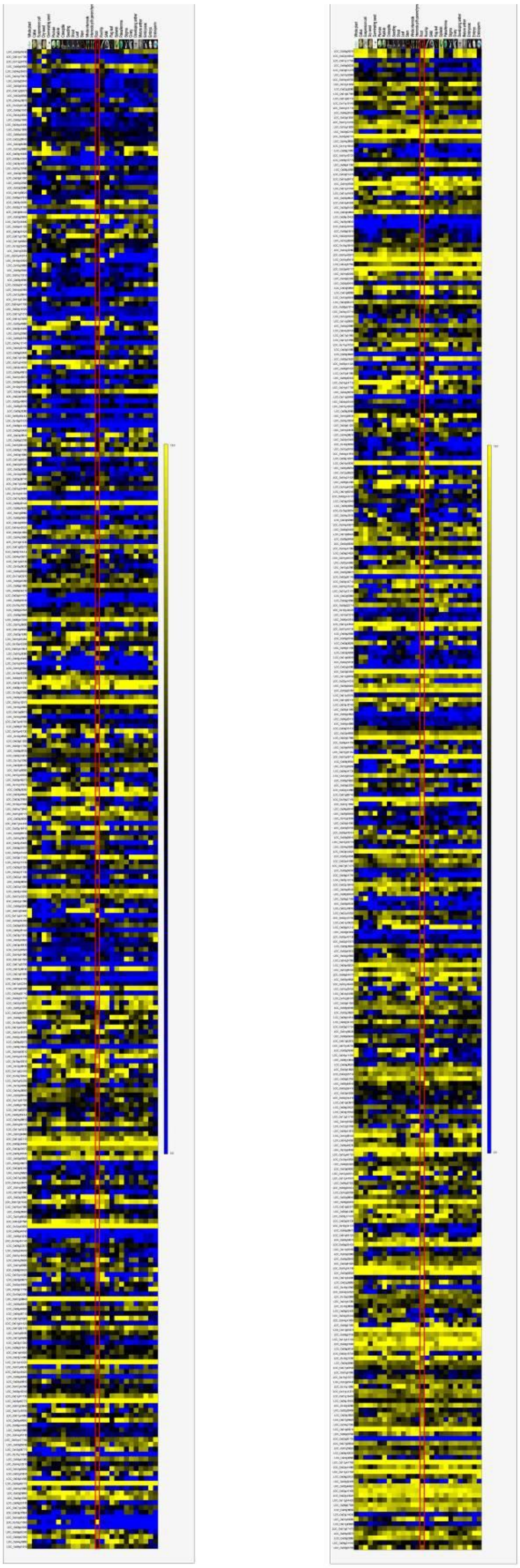

Figure S11 Heat map of differentially expressed genes during different growth stages in rice using microarray data

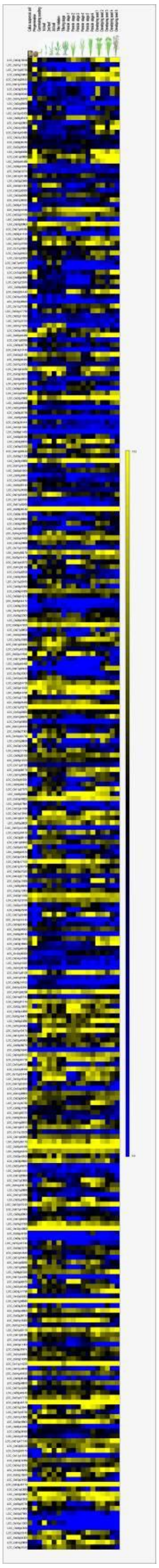

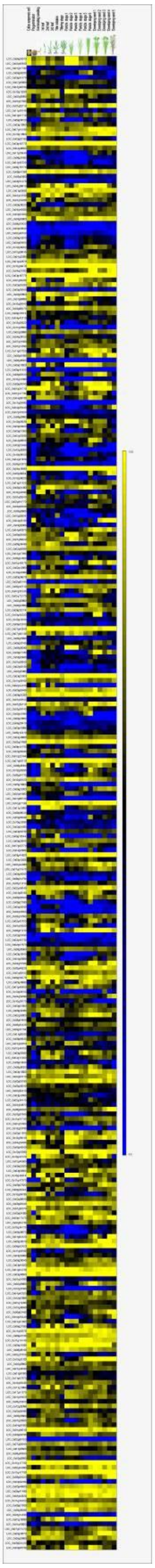

Figure S12 Expression analysis of differentially expressed genes in two particular microarray GSE6901 and GSE7256

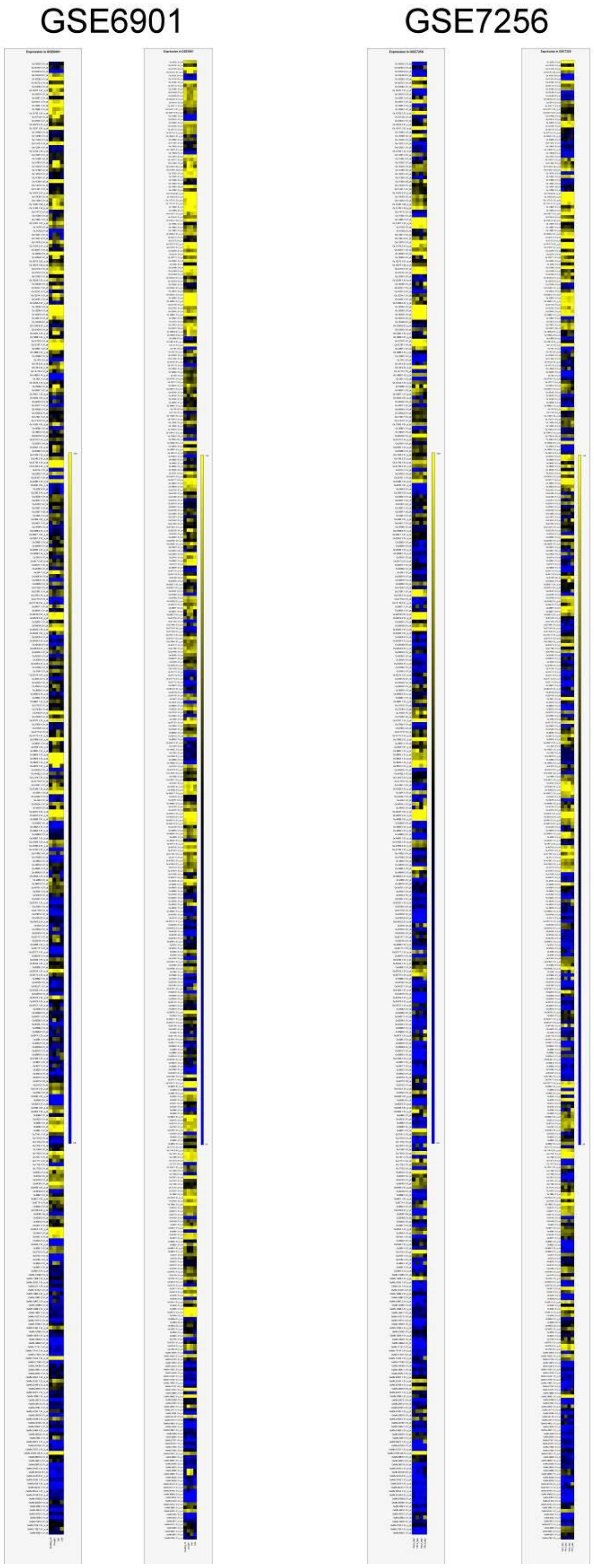

Figure S13 Gene function classification of differentially expressed genes identified in this study

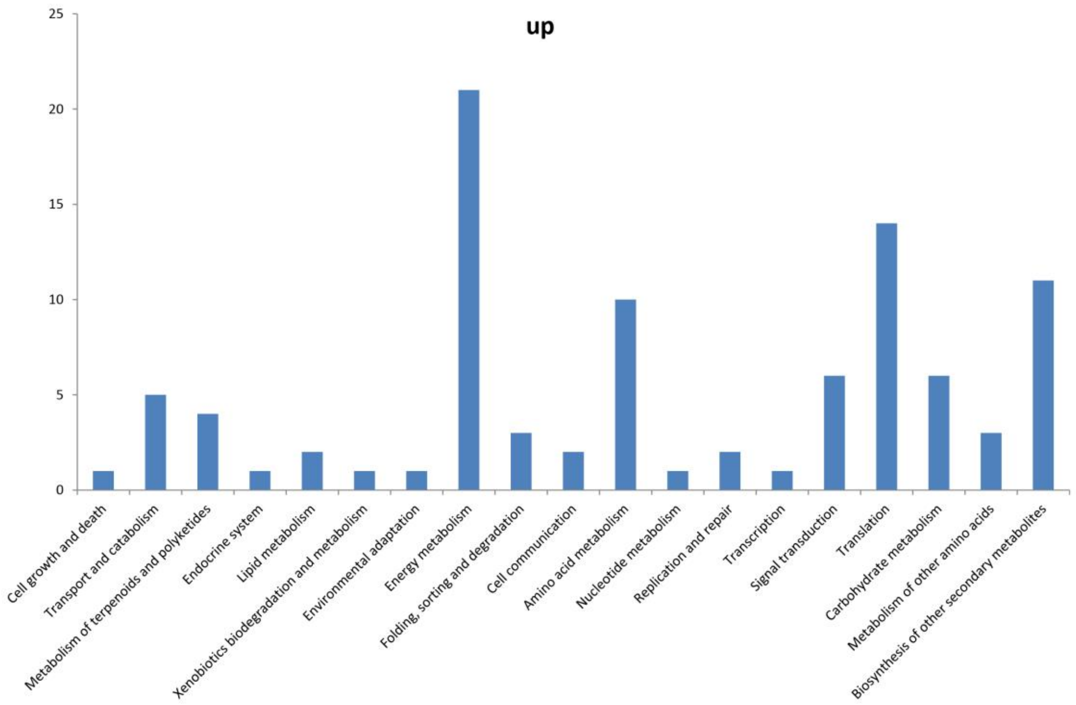

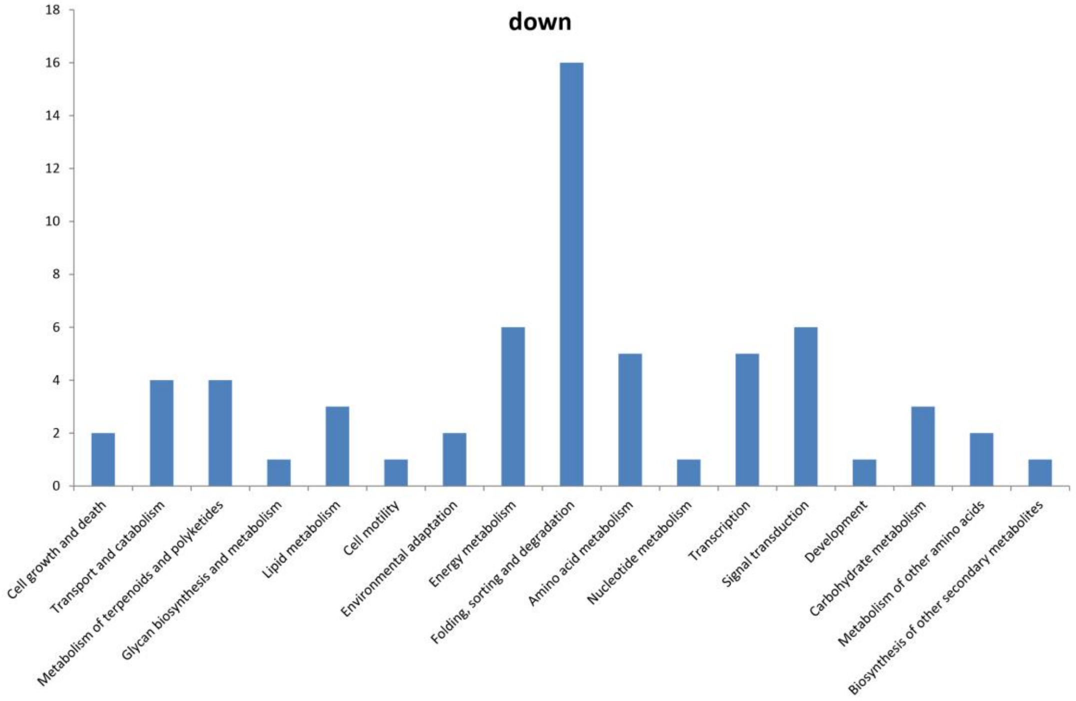

Figure S14 Effect of low K^+ stress at seedling stage on activities of SOD (A), activities of POD (B) and MDA contents (C) of Nipponbare for 24 h, 48 h, and 84 h. Error bars represent ± SE (n=3).

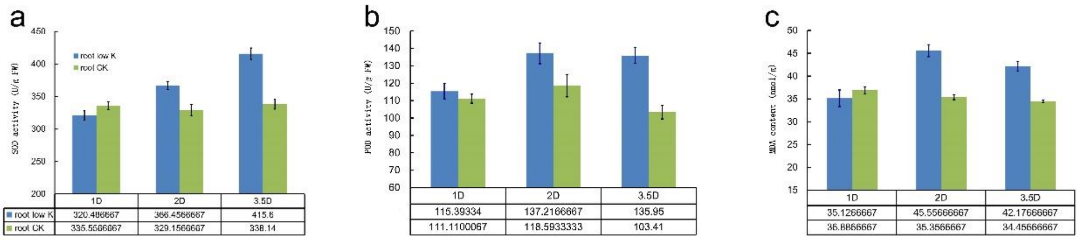

Supplement: Supplementary file 1 — SUPPLEMENTARY MATERIAL [file 41598_2017_5887_MOESM1_ESM.pdf]
